# Supplementary material for: SARS-CoV-2-specific monoclonal antibody AER002 in Long COVID: an exploratory randomized phase 2a mechanistic trial
Source: Nat Commun. 2026 Sep 30;17:11205. doi: 10.1038/s41467-026-77925-y (PMC13720204; doi:10.1038/s41467-026-77925-y)
Supplement: Supplementary file 1 [file 41467_2026_77925_MOESM1_ESM.pdf]

Supplementary Information

Supplement to:

SARS-CoV-2-Specific Monoclonal Antibody AER002 in Long COVID: An Exploratory Randomized Phase 2a Mechanistic Trial

Peluso MJ, Ryder D, Dalhuisen T, et al

Table of Contents:

Supplementary Tables.. 2-18
Supplementary Figures..19-23
Study Protocol.. 24-77
CONSORT Checklist 78-80

Supplementary Table 1. Pre-treatment values for continuous patient reported outcomes, performance measures, and laboratory measurements.

Patient Reported Outcome	AER002*	Placebo*	Mean difference	95% CI	p-value
**PROMIS-29 PHSS** (T-score)	39.7	38.0	1.7	(-5.4, 8.9)	0.62
**PROMIS-29 MHSS** (T-score)	37.5	37.6	-0.1	(-4.9, 4.6)	0.96
**QoL 100-point VAS**	49.1	44.2	4.9	(-9.0, 18.8)	0.48
**QoL EQ-5D-5L Index**	0.48	0.49	-0.01	(-0.21, 0.18)	0.90
**DASI**	36.5	28.6	7.9	(-3.9, 19.7)	0.18
**COMPASS-31**	20.2	28.5	-8.3	(-19.6, 2.9)	0.14
**WHO-DAS 2.0**	17.3	18.8	-1.5	(-7.2, 4.2)	0.60
**ECog-39**	2.29	2.49	-0.20	(-0.72, 0.30)	0.42
**6MWT (meters)**	412	400	12	(-76.9, 101)	0.78
**CNS-VS NCI**	95.6	93.2	2.4	(-4.6, 9.3)	0.49
**Laboratory measurements**	**AER002****	**Placebo****	**Percent difference**	**95% CI**	**p-value**
**CRP** (mg/mL)	0.64	0.69	-7.1	(-58.8, 109.5)	0.86
**ESR** (mm/hr)	9.52	8.50	12.1	(-40.1, 109.8)	0.71
**D-Dimer** (mg/L FEU)	0.29	0.33	-13.2	(-27.7, 4.16)	0.12
**Fibrinogen** (mg/dL)	288	298	-3.2	(-16.7, 12.4)	0.66

*Estimated marginal means **Estimated marginal geometric means

Supplementary Table 2. Related adverse events (AEs).

	AER002				Placebo			
Adverse Event (AE)	Mild (no. AEs)	Moderate (no. AEs)	Participants (n = 24)	%	Mild (no. AEs)	Moderate (no. AEs)	Participants (n = 12)	%
**Cardiovascular**								
Hypotension	0	0	0	0.0	1	0	1	8.3
**Dermatologic**								
Localized rash	0	1	1	4.2	0	0	0	0.0
Localized itchiness	0	0	0	0.0	0	1	1	8.3
**ENT**								
Cold sores	0	1	1	4.2	1	1	2	16.7
Sore throat	0	0	0	0.0	1	0	1	8.3
**Gastrointestinal**								
Constipation	0	0	0	0.0	1	0	1	8.3
Diarrhea	0	0	0	0.0	3	0	3	25.0
**General/systemic**								
Post-exertional malaise	2	1	3	12.5	0	4	4	33.3
Fatigue	2	0	2	8.3	0	0	0	0.0
Chills	1	0	1	4.2	0	0	0	0.0
Subjective fever	1	0	1	4.2	0	0	0	0.0
Pre-syncope	0	0	0	0.0	1	0	1	8.3
**Musculoskeletal**								
Muscle pain/aches	4	1	5	20.8	0	0	0	0.0
Pain at infusion site	1	0	1	4.2	1	0	1	8.3
Muscle twitching	1	0	1	4.2	0	0	0	0.0
**Neurologic**								
Headache	1	1	2	8.3	0	1	1	8.3
Trouble with balance/feeling unsteady	1	0	1	4.2	0	1	1	8.3
Dizziness	0	1	1	4.2	0	0	0	0.0
Head pressure	0	1	1	4.2	0	0	0	0.0
Lightheadedness	0	1	1	4.2	0	0	0	0.0
Burning sensation in feet	0	0	0	0.0	1	0	1	8.3
Tingling	0	0	0	0.0	1	0	1	8.3
**Ocular**								
Blurry vision	0	1	1	4.2	0	0	0	0.0
**Total**	14	9	—	—	11	8	—	—

Supplementary Table 3. Unrelated adverse events (AEs).

	AER002					Placebo				
**Adverse Event (AE)**	**Mild** (no. AEs)	**Moderate** (no. AEs)	**Severe** (no. AEs)	**Participants** (n = 24)	**%**	**Mild** (no. AEs)	**Moderate** (no. AEs)	**Severe** (no. AEs)	**Participants** (n = 12)	**%**
**Cardiovascular**										
Superficial thrombophlebitis	1	0	0	1	4.2	0	0	0	0	0.0
Hypertension	0	0	0	0	0.0	1	0	0	1	8.3
**Dermatologic**										
Bruise	2	0	0	2	8.3	1	0	0	1	8.3
Skin pain	0	1	0	1	4.2	0	0	0	0	0.0
**ENT**										
Congestion	1	0	0	1	4.2	0	0	0	0	0.0
Sinusitis	0	1	0	1	4.2	0	0	0	0	0.0
Bronchitis	0	0	0	0	0.0	0	2	0	1	8.3
Chronic rhinosinusitis	0	0	0	0	0.0	0	1	0	1	8.3
**Gastrointestinal**										
Gastroenteritis	1	2	0	2	8.3	1	0	0	1	8.3
Diarrhea	1	0	0	1	4.2	0	0	0	0	0.0
**General/systemic**										
Pre-syncope	1	1	0	2	8.3	0	0	0	0	0.0
Fatigue	0	1	0	1	4.2	0	0	0	0	0.0
**Genitourinary**										
Dysuria	1	0	0	1	4.2	0	0	0	0	0.0
Kidney stone	0	1	0	1	4.2	0	0	0	0	0.0
Urinary tract infection	0	1	0	1	4.2	0	0	0	0	0.0
Flank pain	0	0	0	0	0.0	1	0	0	1	8.3
**Infectious**										
Non-specific viral infection	5	2	0	7	29.2	2	1	0	2	16.7
SARS-CoV-2 infection - confirmed	1	5	0	6	25.0	2	2	0	4	33.3
SARS-CoV-2 infection - suspected[†]	0	1	0	1	4.2	1	0	0	1	8.3
Skin/soft tissue infection	0	1	0	1	4.2	1	0	0	1	8.3
Rebound COVID-19	0	0	0	0	0.0	0	1	0	1	8.3
Small intestinal bacterial overgrowth	0	0	0	0	0.0	0	1	0	1	8.3
Suspected oral infection	0	0	0	0	0.0	0	1	0	1	8.3
**Laboratory abnormality**										
Decreased fibrinogen	3	0	0	2	8.3	0	0	0	0	0.0
Hypoglycemia	1	0	0	1	4.2	0	0	0	0	0.0
**Musculoskeletal**										
Fibromyalgia	1	0	0	1	4.2	0	0	0	0	0.0
Muscle pain/aches	0	1	0	1	4.2	0	0	0	0	0.0
Pain	0	2	0	1	4.2	0	0	0	0	0.0
Pelvic congestion syndrome	0	1	0	1	4.2	0	0	0	0	0.0
Gout flare	0	0	0	0	0.0	0	1	0	1	8.3
Rotator cuff tear	0	0	0	0	0.0	0	1	0	1	8.3
Torn meniscus	0	0	0	0	0.0	0	1	0	1	8.3
Torn patellar cartilage	0	0	0	0	0.0	0	1	0	1	8.3
Torn pulley ligament	0	0	0	0	0.0	0	1	0	1	8.3
**Neurologic**										
Brain fog	0	1	0	1	4.2	0	0	0	0	0.0
Intractable chronic migraine with aura	0	0	1	1	4.2	0	0	0	0	0.0
Numbness	0	1	0	1	4.2	0	0	0	0	0.0
Abnormal brain MRI	0	0	0	0	0.0	0	1	0	1	8.3
**Ocular**										
Keratoconjunctivitis sicca	0	0	0	0	0.0	1	0	0	1	8.3
Light sensitivity	0	0	0	0	0.0	0	1	0	1	8.3
**Total**	19	23	1	—	—	11	16	0	—	—

[†] Had known exposure to SARS-CoV-2, but subsequently tested negative

Supplementary Table 4. Linear mixed effect model results for continuous patient reported outcomes, performance measures and laboratory measurements for Day 360.

Patient Reported Outcome	Day	AER002*	Placebo*	Mean difference	95% CI	p-value
**PROMIS-29 PHSS** (T-score)	360	42.6	46.6	-4.0	(-8.9, 0.96)	0.11
**PROMIS-29 MHSS** (T-score)	360	43.6	45.4	-1.8	(-7.1, 3.6)	0.52
**QoL 100-point VAS**	360	58.6	62.3	-3.8	(-15.6, 8.1)	0.53
**QoL EQ-5D-5L Index**	360	0.65	0.63	0.02	(-0.15, 0.19)	0.84
**DASI**	360	39.8	43.3	-3.5	(-11.2,4.2)	0.37
**COMPASS-31**	360	17.4	20.7	-3.3	(-11.7, 5.2)	0.44
**WHO-DAS 2.0**	360	12.4	11.7	0.6	(-4.2, 5.5)	0.78
**ECog-39**	360	1.94	1.99	-0.05	(-0.41, 0.30)	0.76
**6MWT** (meters)	360	430	474	-44.0	(-96.0, 8.2)	0.10
**CNS-VS NCI**	360	102.8	102.9	-0.1	(-4.8, 4.6)	0.97
**Laboratory Measurements**	**Day**	**AER002****	**Placebo****	**Percentage Difference**	**95% CI**	**p-value**
**CRP** (mg/mL)	360	0.76	1.50	-49.3	(-77, 14)	0.10
**ESR** (mm/hr)	360	7.4	9.5	-21.5	(-53, 32)	0.36
**D-Dimer** (mg/L FEU)	360	0.31	0.34	-9.8	(-29, 16)	0.42
**Fibrinogen** (mg/dL)	360	289	309	-3.5	(-14.4, 8.7)	0.55

*Estimated marginal means **Estimated marginal geometric means. Linear mixed effect models include visit terms for D360, which was included as part of the safety analysis (most participants were unblinded following D180).

Supplementary Table 5. AER002 population pharmacokinetic (PK) model parameters.

Parameter[1,2]	Estimate	90% C.I.[3]
θ_{CL}	0.0655	0.0530 – 0.0863
$\theta_{V\ Central}$	3.95	2.78 – 5.62
θ_{Q}	13.6	0.392 – 28.1
$\theta_{V\ Peripheral}$	3.18	1.93 – 5.38
$\theta_{Male\ on\ V\ Peripheral}$ [4]	1.96	1.27 – 2.88
Ω_{CL}	0.354	0.049
$\Omega_{V\ Peripheral}$	0.640	0.246 – 1.08
$\sigma_{Proportional\ Error}$	0.228	0.122 – 0.285
$\sigma_{Additive\ Error}$	12.1	5.14 – 20.4

1. θ's are the typical population estimates, Ω's are the estimates for between subject variability and σ's are the estimates for residual unexplained variability.
2. Clearance (CL) in L/day, Volume of Distribution (V) in L/kg, Inter-compartmental CL (Q) in L/day
3. C.I. is the confidence interval from bootstrap
4. Covariate expressed in the multiplicative effect on peripheral volume of distribution

Supplementary Table 6. Per protocol linear mixed effect model results for continuous patient reported outcomes, performance measures and laboratory measurements.

Patient Reported Outcome	Day	AER002*	Placebo*	Mean difference	95% CI	p-value
**PROMIS-29 PHSS** (T-score)	90	40.8	44.1	-3.3	(-8.5, 1.7)	0.18
**PROMIS-29 MHSS** (T-score)	90	41.5	43.1	-1.6	(-6.0, 2.7)	0.45
**QoL 100-point VAS**	90	53.4	58.9	-5.5	(-16.3, 5.3)	0.31
**QoL EQ-5D-5L Index**	90	0.55	0.63	-0.08	(-0.3, 0.1)	0.34
**DASI**	90	36.3	38.8	-2.5	(-10.4, 5.5)	0.53
**COMPASS-31**	90	17.8	15.4	2.4	(-5.9, 10.9)	0.55
**WHO-DAS 2.0**	90	16.9	13.4	3.5	(-0.01, 7.1)	0.05
**ECog-39**	90	2.04	1.94	0.1	(-0.24, 0.45)	0.53
**6MWT**	90	419	453	-34	(-83.4, 17.1)	0.19
**CNS-VS NCI score**	90	101	101.2	-0.2	(-5.3, 4.9)	0.94
**Laboratory measurements**	**Day**	**AER002****	**Placebo****	**Percentage difference**	**95% CI**	**p-value**
**CRP** (mg/mL)	90	0.78	0.63	22.6	(-42, 156)	0.58
**ESR** (mm/hr)	90	8.3	9.1	-8.5	(-43.3, 48.2)	0.71
**D-Dimer** (mg/L FEU)	90	0.29	0.30	-0.9	(-9.7, 8.7)	0.83
**Fibrinogen** (mg/dL)	90	306	288	6.1	(-4.9, 18.4)	0.28

*Estimated marginal means **Estimated marginal geometric means. Per protocol analyses exclude those with either a confirmed or suspected reinfection before the primary endpoint (D90)

Supplementary Table 7. Optional Gut Biopsy RNAScope Results

PID	Treatment	Sex	Age (yrs)	Reinfection between biopsies	Timepoint (baseline vs. post-infusion)	Days since initial infection	Days since most recent infection	Spike RNA (SS)	orf1ab RNA (DS)
**19**	Placebo	Male	70-79	-	Baseline	1315	1315	-	-
					Post-infusion	1434	1434	-	-
**5**	Placebo	Female	50-59	-	Baseline	1163	1163	-	-
					Post-infusion	1254	1254	-	-
**14**	AER002	Male	50-59	+	Baseline	1093	1093	-	-
					Post-infusion	1198	21	+	+
**11**	AER002	Male	50-59	-	Baseline	255	255	-	-
					Post-infusion	365	365	-	-
**10**	AER002	Male	40-49	+	Baseline	515	515	-	-
					Post-infusion	620	28	+	-
**24**	AER002	Female	30-39	-	Baseline	485	485	-	-
					Post-infusion	590	590	-	-
**35**	AER002	Female	40-49	-	Baseline	752	318	-	-
					Post-infusion	871	437	-	-
**28**	AER002	Male	30-39	-	Baseline	721	721	-	-
					Post-infusion	847	847	-	-
**2**	AER002	Male	60-69	-	Baseline	559	559	-	-
					Post-infusion	671	671	-	-
**25**	AER002	Female	40-49	-	Baseline	515	515	-	-
					Post-infusion	627	627	-	-
**6**	AER002	Male	30-39	-	Baseline	448	448	-	-
					Post-infusion	546	546	-	-
**16**	Placebo	Female	40-49	+	Baseline	545	123	-	-
					Post-infusion	650	57	-	-
**42**	Placebo	Male	40-49	-	Baseline	811	811	-	-
					Post-infusion	930	930	-	-
**45**	AER002	Male	40-49	+	Baseline	992	992	-	-
					Post-infusion	1111	21	+	+
**44**	AER002	Male	50-59	-	Baseline	1238	718	+	-
					Post-infusion	1378	858	-	-
**48**	AER002	Female	30-39	-	Baseline	729	396	-	-
					Post-infusion	833	500	-	-
**47**	Placebo	Female	40-49	+	Baseline	1093	1093	-	-
					Post-infusion	1288	18	-	-
**12***	AER002	Female	40-49	-	Baseline	1287	112	-	-

* Declined post biopsy

Supplementary Table 8. Multiple logistic regression parameters of each baseline antibody level and AER002 exposure (AUC: area under the concentration-time curve) on likelihood of self-reported response, as depicted in Figure 9b.

Parameter	Odds ratio (point estimate)	95% confidence interval
**Anti-N Ab**		
Low Ab	1.00 (ref)	N/A
High Ab	13.33	1.39 to 237.45
AER002 AUC (per 500 mg*day/L) in low Ab	2.20	0.69 to 9.87
AER002 AUC (per 500 mg*day/L) in high Ab	0.86	0.07 to 1.72
**Anti-S Ab**		
Low Ab	1.00 (ref)	N/A
High Ab	116.14	5.13 to 27459.08
AER002 AUC (per 500 mg*day/L) in low Ab	5.77	1.38 to 102.11
AER002 AUC (per 500 mg*day/L) in high Ab	0.63	5.07×10^{-3} to 0.76
**Anti-S1 Ab**		
Low Ab	1.00 (ref)	N/A
High Ab	74.41	3.44 to 12212.14
AER002 AUC (per 500 mg*day/L) in low Ab	4.72	1.24 to 60.31
AER002 AUC (per 500 mg*day/L) in high Ab	0.82	0.01 to 1.25
**Anti-RBD Ab**		
Low Ab	1.00 (ref)	N/A
High Ab	130.93	5.78 to 31478.92
AER002 AUC (per 500 mg*day/L) in low Ab	5.59	1.38 to 97.8
AER002 AUC (per 500 mg*day/L) in high Ab	0.51	4.19×10^{-3} to 0.65

Supplementary Table 9. Complete inclusion and exclusion criteria.

**Inclusion Criteria**
1. Male, female, or transgender ≥18 years of age at Screening.
2. Enrolled or willing to enroll and complete at least 1 visit in the UCSF Long-term Impact of Infection with Novel Coronavirus (LIINC) study. Any adult who has been infected with SARS-CoV-2 or has ever received or is eligible to receive a SARS-CoV-2 vaccination, does not have chronic anemia, and who is able to provide written informed consent, is eligible to participate in LIINC.
3. History of confirmed acute SARS-CoV-2 infection, whose initial infection meets criteria as outlined below:<br>a. Report of a positive nucleic acid amplification test (NAAT) and/or a positive SARS-Cov-2 antigen rapid diagnostic test (RDT). Written proof of the test will be requested but is not required as long as the participant attests to the positive test.<br>AND<br>b. Long COVID (see below) attributed to SARS-CoV-2 infection with a variant against which AER002 is known to have neutralizing activity. In cases in which the variant is not known from prior viral sequencing (most cases), the SARS-CoV-2 infection to which Long COVID is attributed should have been dated prior to August 15, 2022.<br><br>*Note: For individuals who may be chronically infected (with persistent viral shedding from nasopharynx), a genotype is required to confirm that they have a AER002-susceptible variant.*
4. Clinical evidence of Long COVID, as confirmed by the investigator's assessment.<br>a. At least two symptoms (see list) that are new or worsened since the time of SARS-CoV-2 infection, not known to be attributable to another cause upon assessment by the PI. At least two symptoms from those listed here must be present: systemic symptoms (e.g., fatigue, chills, post-exertional malaise), neurocognitive symptoms (e.g., trouble with memory/concentration ("brain fog"), headache, dysautonomia/postural orthostatic tachycardia syndrome, dizziness, unsteadiness, neuropathy, sleep disturbance), cardiopulmonary symptoms (e.g., chest pain, palpitations, shortness of breath, cough, fainting spells), musculoskeletal symptoms (e.g., muscle aches, joint pain), gastrointestinal symptoms (e.g., nausea, diarrhea). Although other symptoms (e.g., skin rash, hair loss, mental health symptoms, trouble with smell/taste, genitourinary symptoms) will be recorded and tracked, at least two core symptoms listed above must be present. Note: the two symptoms can be from within the same category (for example, brain fog and headache).<br>AND<br>b. Symptoms must have been present for at least 60 days prior to screening. Symptoms that wax and wane must have been initially present at least 60 days prior to screening<br>AND<br>c. Symptoms must be reported to be at least somewhat bothersome and to have an impact on quality of life and/or everyday functioning.
5. Long COVID attributed to a SARS-CoV-2 infection prior to August 15, 2022. Note: While individuals re-infected with SARS-CoV-2 after August 15, 2022 will not be excluded, the SARS-CoV-2 infection after which Long COVID symptoms began must pre-date August 15, 2022.
6. Not currently hospitalized.
7. Body mass index (BMI) 18 to 50 kilograms/meter squared (kg/m2), inclusive, at the time of screening.

8. In otherwise stable health, as assessed by the investigator within 28 days prior to Screen, based on medical history, physical assessment, laboratory findings, and vital signs.
9. Participants who are of childbearing potential (CBP) and male participants with sexual partner(s) who are females of CBP must agree to use adequate contraception from study consent through 360 days after dosing. Adequate contraception is defined as using hormonal contraceptives or an intrauterine device combined with at least 1 of the following forms of contraception: a diaphragm, a cervical cap, or a condom. Total abstinence from vaginal intercourse, in accordance with the lifestyle of the participant, is also acceptable.
10. Willingness and ability to comply with the study protocol. This includes reliable transportation and sufficient time to attend all visits.
11. Written informed consent (and assent when applicable) obtained from subject or subject's legal representative and ability for subject to comply with the requirements of the study.

**Exclusion Criteria**
1. Current acute SARS-CoV-2 infection at the time of screening.
2. Long COVID attributed to a SARS-CoV-2 infection after August 15, 2022.
3. Previously received treatment or prophylaxis with a SARS-CoV-2-specific mAb, or plan to receive such treatment before exiting the study.
4. Previously received COVID-19 convalescent plasma treatment within 60 days prior to planned Day 0 or plan to receive such treatment before exiting the study.
5. Plans to receive any investigational or approved vaccine or booster for SARS-CoV-2 within 60 days prior to planned Day 0 or before Day 30 following planned Day 0.
6. Active cardiovascular disease, defined as known prior:<br>a. Myocardial infarction within 90 days of screening<br>OR<br>b. Coronary artery bypass within 90 days of screening<br>OR<br>c. Current heart failure with reduced ejection fraction (<45%)<br>OR<br>d. Current pulmonary arterial hypertension.
7. Known stroke within 3 months prior to planned Day 0.
8. Known active bacterial, fungal, viral, or other infection besides SARS-CoV-2 requiring treatment within the 28 days prior to planned Day 0 and meeting criteria for systemic involvement upon review by the PI. Note: Mild or limited infections such as uncomplicated urinary tract or yeast infections, sexually transmitted infections, and mild dermatophyte infections may be reviewed with the medical monitor but are not exclusionary.
9. Major surgery within 6 months prior to planned Day 0 or planned major surgery during the first 180 days following planned Day 0.
10. History of unplanned hospitalization for >24 hours within 28 days prior to Screening.
11. Active Hepatitis B (Hep B) infection (defined as Hep B surface antigen (sAg) positive).<br><br>*Note: A known positive Hep B core antibody (cAb) in the absence of positive sAg is not considered exclusionary.*
12. Active Hepatitis C (Hep C) infection (defined as Hep C Ab positive or indeterminate with detectable Hep C RNA).<br><br>*Note: Those with cured Hep C (Ab positive or indeterminate but negative Hep C RNA) will remain eligible.*

13. HIV infection that is known to be unstable (two or more consecutive plasma HIV RNA values >48 copies/mL in the 6 months prior to screen) or uncontrolled (not on antiretroviral therapy (ART)). In addition, people with HIV who have a current CD4+ T cell count < 200 cells/uL, a history of AIDS defining illness without immune reconstitution, or clinical manifestations of symptomatic HIV will be excluded.
14. Severe coagulopathy that would prevent an infusion (international normalized ratio ((INR) >2.0, history of hemophilia).
15. Severe anemia (hemoglobin <9 grams/deciliter (g/dL)).
16. Moderate or severe immunocompromise, according to the current NIH COVID-19 Treatment Guidelines as of March 6, 2023. This includes the following:<br>a. Receiving active treatment for solid tumor or hematologic malignancy, including use of systemic chemotherapy for treatment of cancer within the year prior to screening,<br>b. Prior solid-organ transplant with active immunosuppressive therapy<br>c. CAR-T cell therapy or hematopoietic cell transplant, on immunosuppressive therapy or transplant within the prior 2 years,<br>d. Primary immunodeficiency syndromes,<br>e. Advanced or untreated HIV infection (see above),<br>f. Or on active high-dose corticosteroids (i.e., >= 20mg prednisone or equivalent daily per day for >= 2 weeks).
17. Known prior diagnosis of myalgic encephalomyelitis/chronic fatigue syndrome (ME/CFS), preceding and not related to SARS-CoV-2 infection and not worsened since SARS-CoV-2 infection.
18. Known prior diagnosis of dysautonomia, preceding and not related to SARS-CoV-2 infection and not worsened since SARS-CoV-2 infection.
19. History of anaphylaxis or hypersensitivity upon receiving IV antibody infusions in the past.
20. Known allergy to any components used in the formulation of the intervention.
21. History of anaphylaxis or similar significant allergic reaction to prescription or non-prescription drugs or food products. Similarly, presence of severe atopic conditions as assessed by the PI represents significant risk for allergic reaction.
22. Pregnant, breastfeeding, or unwilling to practice birth control abide by the contraception requirements outlined in the inclusion criteria.
23. Participation in a clinical trial with receipt of an investigational product within 28 days or 5 half-lives (whichever is longer) prior to planned Day 0. For PET tracers specifically, which have a short half-life and are not biologically active outside this window, elapse of 5 half-lives prior to the planned D0 infusion is sufficient.
24. Current alcohol or illicit drug use as determined by the investigator to preclude participation.
25. Presence of a condition or abnormality that in the opinion of the Investigator would compromise the safety of the patient or the quality of the data.
26. Study site personnel directly affiliated with the study or family of directly involved personnel.
27. History of cytokine release syndrome (CRS) secondary to infection and/or medication.

Supplementary Table 10. Descriptions of pre-specified primary and secondary outcomes.

Primary Outcome	Day	Description
Patient-Reported Outcomes Measurement Information System (PROMIS)-29 Physical Health Summary Score (PROMIS-29 PHSS)	90	This measure will evaluate whether there is a difference between treatment with AER002 versus placebo in baseline adjusted mean PROMIS-29 Physical Health Summary Score at Day 90 post-infusion. PROMIS-29 is a validated scale assessing physical function, anxiety, depression, fatigue, sleep disturbance, ability to participate in social activities, and pain. Each domain is scored on a 5-point scale (without any difficulty, with a little difficulty, with some difficulty, with much difficulty, unable to do). A T-score is calculated from each individual domain. A T score of 50 represents the mean for US general adult population, and 10 is the standard deviation. A lower T score indicates worse physical health.

Category	Secondary Outcome	Day	Description
Physical health and quality of life	Patient-Reported Outcomes Measurement Information System (PROMIS)-29 Physical Health Summary Score (PROMIS-29 PHSS)	30, 180	This measure will evaluate whether there is a difference between treatment with AER002 versus placebo in baseline adjusted mean PROMIS-29 Physical Health Summary Score at Day 30 and Day 180 post-infusion. PROMIS-29 is a validated scale assessing physical function, anxiety, depression, fatigue, sleep disturbance, ability to participate in social activities, and pain. Each domain is scored on a 5-point scale (without any difficulty, with a little difficulty, with some difficulty, with much difficulty, unable to do). A T-score is calculated from each individual domain. A T score of 50 represents the mean for US general adult population, and 10 is the standard deviation. A lower T score indicates worse physical health.
Physical health and quality of life	Patient-Reported Outcomes Measurement Information System (PROMIS)-29 Mental Health Summary	90	This measure will evaluate whether there is a difference between treatment with AER002 versus placebo in baseline adjusted mean PROMIS-29 Mental Health Summary Score at Day 90 post-infusion. PROMIS-29 is a validated scale

	Score (PROMIS-29 MHSS)		assessing physical function, anxiety, depression, fatigue, sleep disturbance, ability to participate in social activities, and pain. Each domain is scored on a 5-point scale (without any difficulty, with a little difficulty, with some difficulty, with much difficulty, unable to do). A T-score is calculated from each individual domain. A T score of 50 represents the mean for US general adult population, and 10 is the standard deviation. A lower T score indicates worse mental health.
Physical health and quality of life	Patient-Reported Outcomes Measurement Information System (PROMIS)-29 Mental Health Summary Score (PROMIS-29 MHSS)	30, 180	This measure will evaluate whether there is a difference between treatment with AER002 versus placebo in baseline adjusted mean PROMIS-29 Mental Health Summary Score at Day 30 and Day 180 post-infusion. PROMIS-29 is a validated scale assessing physical function, anxiety, depression, fatigue, sleep disturbance, ability to participate in social activities, and pain. Each domain is scored on a 5-point scale (without any difficulty, with a little difficulty, with some difficulty, with much difficulty, unable to do). A T-score is calculated from each individual domain. A T score of 50 represents the mean for US general adult population, and 10 is the standard deviation. A lower T score indicates worse mental health.
Physical health and quality of life	Quality of Life (Global Health Score) 100-point Visual-Analogue Scale	90	This measure will evaluate whether there is a difference between treatment with AER002 versus placebo in the baseline adjusted mean Quality of Life 100-point Visual-Analogue-Scale at Day 90 post-infusion. 0 represents the worst health a person can imagine and 100 represents the best health a person can imagine.
Physical health and quality of life	Quality of Life (5-Item EuroQol EQ-5D-5L) Index Value Score	90	This measure will evaluate whether there is a difference between treatment with AER002 versus placebo in baseline adjusted mean Quality of Life (5-Item EuroQol EQ-

			5D-5L) Index Value Score at Day 90 post-infusion. 5-Item EuroQol EQ-5D-5L questions assess pain/difficulty in day-to-day activities over the past week. The 5-Item EuroQol EQ-5D-5L produces a score that typically ranges from 0 - 1, with a higher score indicating better quality of life.
Physical health and quality of life	World Health Organization Disability Assessment Schedule 2.0 (WHO-DAS 2.0)	90	This measure will evaluate whether there is a difference between treatment with AER002 versus placebo in baseline adjusted mean WHO-DAS 2.0 score at Day 90 post-infusion. The World Health Organization Disability Assessment Schedule 2.0 questionnaire asks about difficulties due to health conditions. Health conditions include diseases or illnesses, other health problems that may be short or long lasting, injuries, mental or emotional problems, and problems with alcohol or drugs. The range is scored from 0-48, with a higher score indicating a higher level of disability.
Overall impression of change	Patient Global Impression of Change (PGIC) Scale	90	This measure will evaluate whether there is a difference between treatment with AER002 versus placebo on the Patient Global Impression of Change (PGIC) scale at Day 90 post-infusion. The self-reported PGIC reflects a patient's belief about the efficacy of treatment. We used a modified PGIC scale which has been used to study pain syndromes and has been employed in other Long COVID clinical trials. It is a common data element developed by the National Institutes of Mental Health. The PGIC ranges from 0 (Much better) to 10 (Much Worse). A score of 5 indicates no change.
Cardiopulmonary symptoms	Duke Activity Status Index (DASI)	90	This measure will evaluate whether there is a difference between treatment with AER002 versus placebo in baseline adjusted mean DASI at Day 90 post-infusion. The Duke Activity Status Index is a patient-reported estimate of functional capacity, maximal oxygen

			consumption (VO2 max) and maximum metabolic equivalent of tasks (METs). The DASI questionnaire produces a score between 0 and 58.2 points, which is linearly correlated with a patient's VO2 max and METs, as measured from cardiopulmonary exercise testing (CPET). It inquires about a person's ability to perform self-care, walk, climb stairs, run, do house and yard work, engage in sexual intercourse, and perform moderate recreational activities. A higher score indicates higher functional capacity.
Cardiopulmonary symptoms	6 Minute Walking Test (6MWT)	90	This measure will evaluate whether there is a difference between treatment with AER002 versus placebo in baseline adjusted mean distance walked on the 6MWT at Day 90 post-infusion. The 6MWT requires an individual to walk at their normal pace for 6 minutes on a marked track (for example, a hallway). Vital signs are assessed, and the total distance covered (in meters) is the primary outcome of interest.
Neurocognitive symptoms and performance	Everyday Cognition Form (ECog-39)	90	This measure will evaluate whether there is a difference between treatment with AER002 versus placebo in baseline adjusted mean ECog-39 score at Day 90 post-infusion. The ECog-39 is an instrument that measures the decline in everyday cognitive and functional abilities that map to six cognitive domains, adapted specifically to describe change in abilities since having COVID. A summary ECog-39 score is calculated scored with a range of 1-4, with a higher score indicating greater cognitive impairment.
Neurocognitive symptoms and performance	Neurocognition Index (NCI) standard score from the CNS-VS	90	This measure will evaluate whether there is a difference between treatment with AER002 versus placebo in baseline adjusted mean NCI standard score from the CNS-VS at Day 90 post-infusion. The CNS Vital Signs is a computer-based

			neurocognitive assessment comprised of seven tests: verbal and visual memory, finger tapping, symbol digit coding, the Stroop Test, a test of shifting attention and the continuous performance test. The battery gives a summary neurocognition index (NCI) score averaging five domain scores (Composite Memory, Psychomotor Speed, Reaction Time, Complex Attention, and Cognitive Flexibility) and representing a global score of neurocognition. NCI scores are normalized scores (mean 100, standard deviation 15) that are age matched relative to other people in a normative sample. A higher score indicates better cognitive function.
Autonomic symptoms and performance	Active Stand Test	90	The active standing test is a non-invasive tool to assess orthostatic hypotension (OH) and postural orthostatic tachycardia syndrome (POTS). In short, blood pressure and heart rate measurements were obtained after 5 minutes of resting supine and 1, 3, 5, and 10 minutes of continuous standing. Abnormal active standing test results were defined as those with a decline of >20 mmHg in systolic or > 10 mmHg in diastolic blood pressure in at least two consecutive measurements, or those with an increase in heart rate > 30 bpm on two consecutive measurements.
Autonomic symptoms and performance	Composite Autonomic Symptom Score-31 (COMPASS-31)	90	This measure will evaluate whether there is a difference between treatment with AER002 versus placebo in baseline adjusted mean COMPASS-31 score at Day 90 post-infusion. COMPASS-31 asks 31 questions related to autonomic dysfunction. The answer to each question generates a numeric score for the question, which is then summed at the end of the questionnaire. A total score out of 100 is generated summarizing orthostatic intolerance, vasomotor, secretomotor, gastrointestinal,

			urinary, pupillomotor, temperature intolerance, and sexual impairment. The total score ranges from 0 to 100 and a higher score indicates more severe autonomic dysfunction.
Laboratory measurements	C-Reactive Protein (CRP)	90	This measure will evaluate whether there is a difference between treatment with AER002 versus placebo in baseline adjusted mean CRP concentration (mg/L) at Day 90 post-infusion.
Laboratory measurements	Erythrocyte Sedimentation Rate (ESR)	90	This measure will evaluate whether there is a difference between treatment with AER002 versus placebo in baseline adjusted mean ESR at Day 90 post-infusion.
Laboratory measurements	D-Dimer	90	This measure will evaluate whether there is a difference between treatment with AER002 versus placebo in baseline adjusted mean D-Dimer at Day 90 post-infusion.
Laboratory measurements	Fibrinogen	90	This measure will evaluate whether there is a difference between treatment with AER002 versus placebo in baseline adjusted mean fibrinogen concentration (mg/dL) at Day 90 post-infusion.

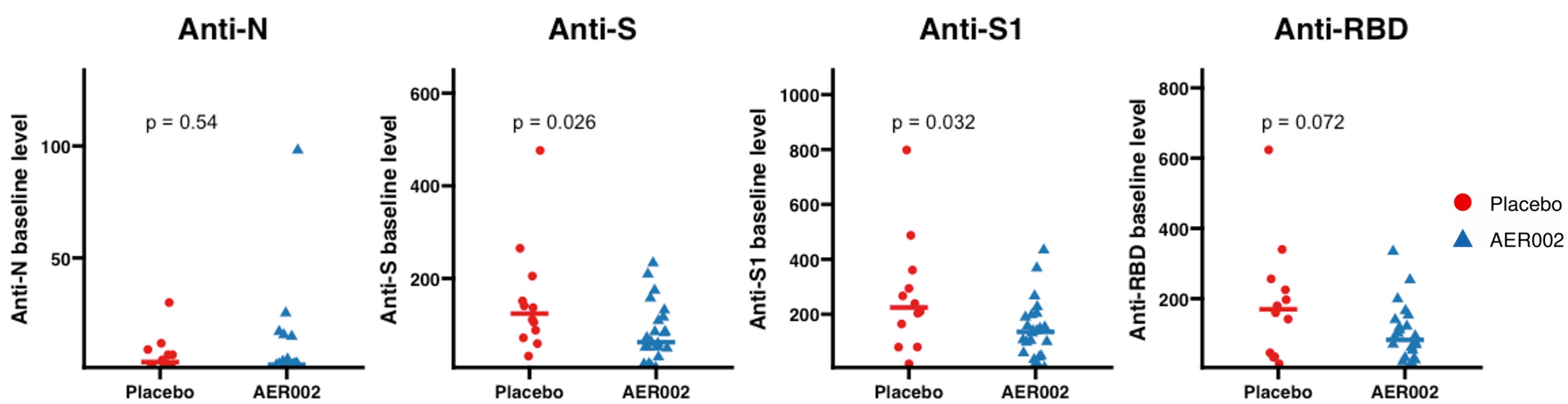

Supplementary Figure 1. Baseline levels of SARS-CoV-2 antibodies AER002 (n = 24) versus placebo (n = 12). From left to right: anti-nucleocapsid (N); anti full-length spike (S); anti-S1 subunit (S1); anti-receptor binding domain (RBD). P values are from two-sided Wilcoxon rank-sum tests, and median values (horizontal line) are shown for each group.

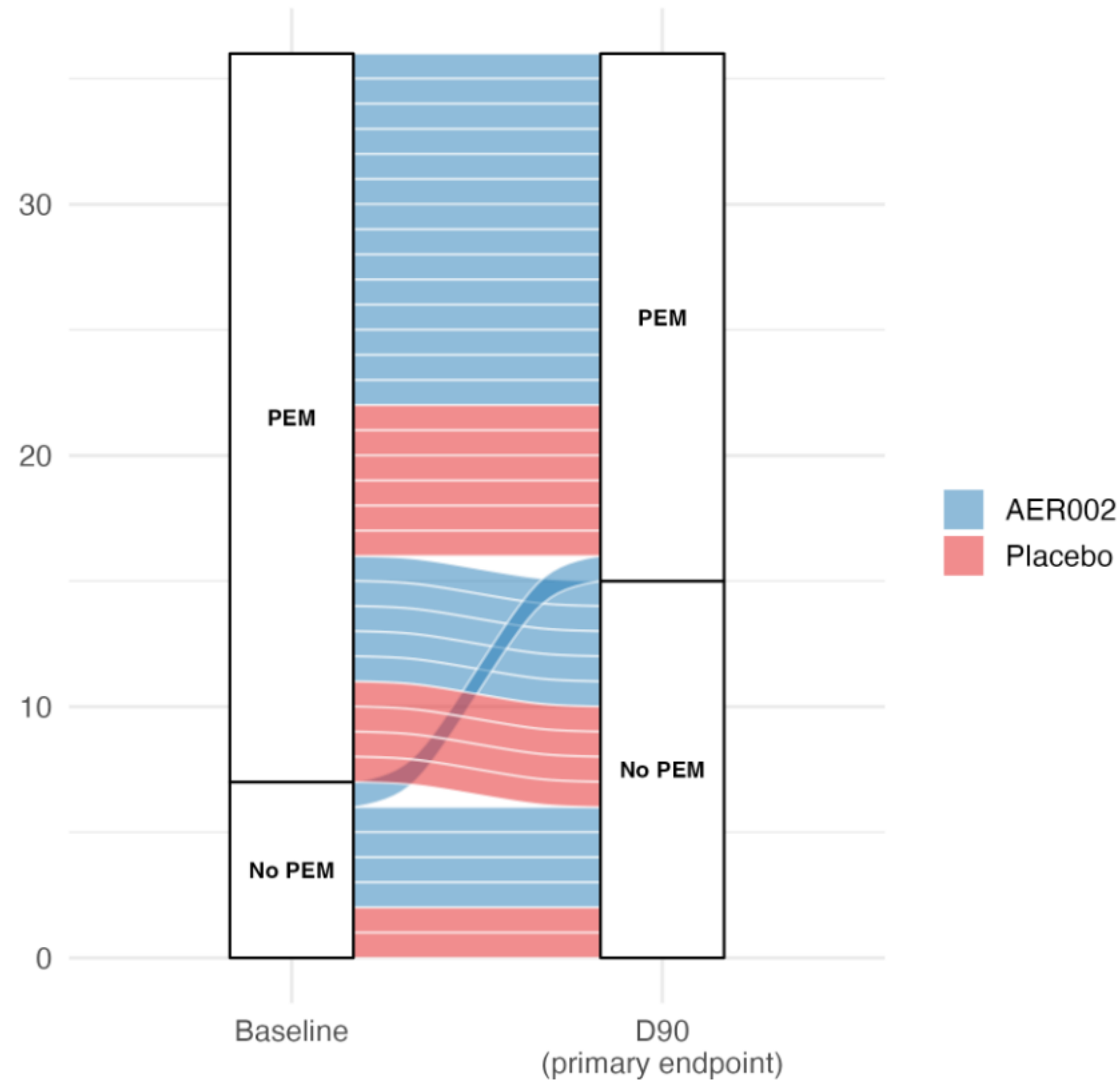

Supplementary Figure 2. Post-exertional malaise (PEM) trends from baseline to D90 AER002 versus placebo. AER002 (n=24); placebo (n=12). Each flow represents one participant.

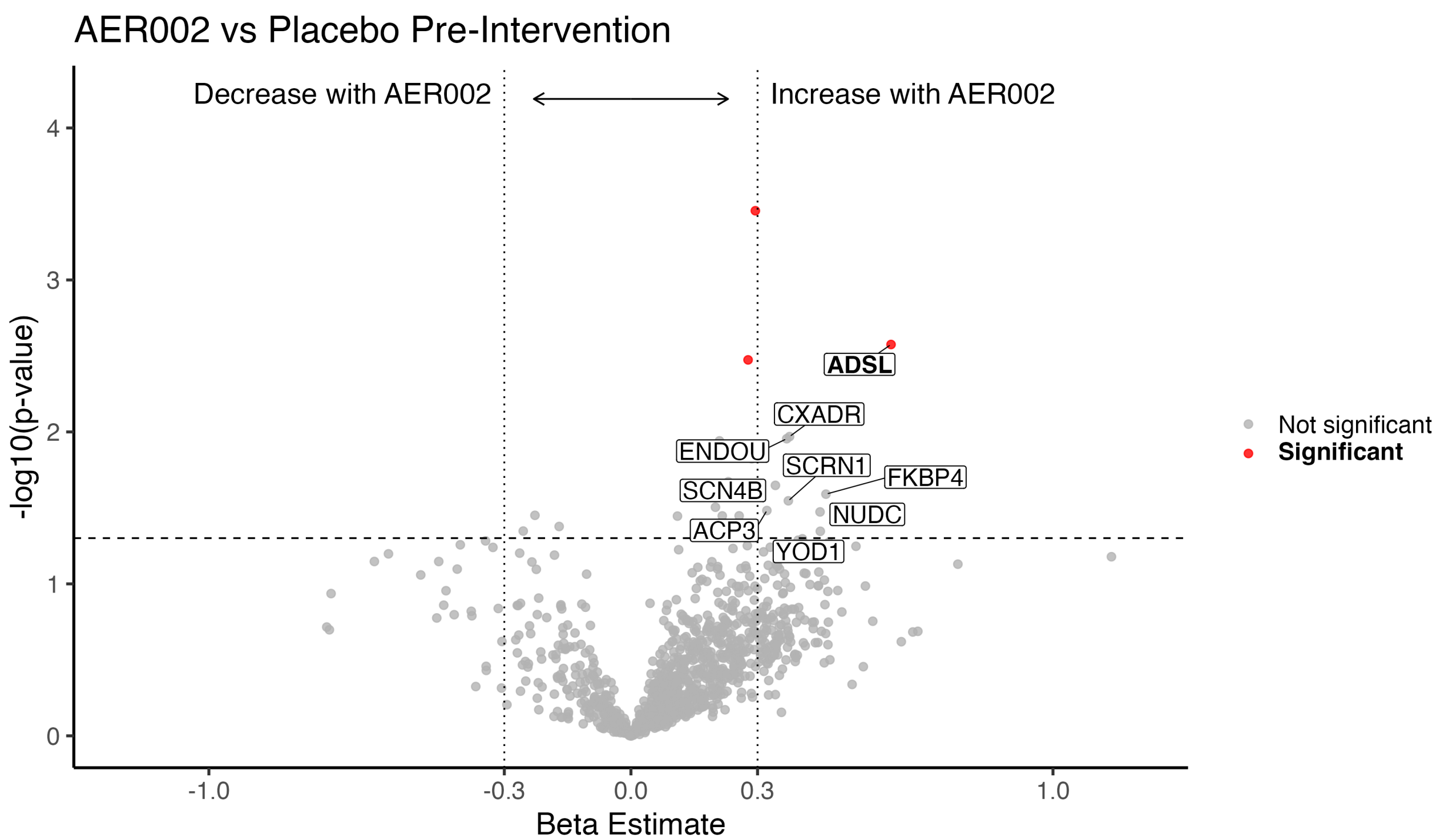

Supplementary Figure 3. Plasma proteomics AER002 versus placebo pre-intervention. Volcano plots showing differential plasma protein expression for AER002 (n=24) versus placebo (n=12) pre-intervention. Differential expression was assessed using protein-specific linear mixed-effects models. All tests were two-sided, and P values were adjusted for multiple comparisons using the Storey q-value method. Significant proteins with q-value < 0.05 are shown in bold red. The horizontal dashed line indicates the unadjusted P-value threshold of 0.05. Vertical dashed lines indicate beta estimates of −0.3 and 0.3.

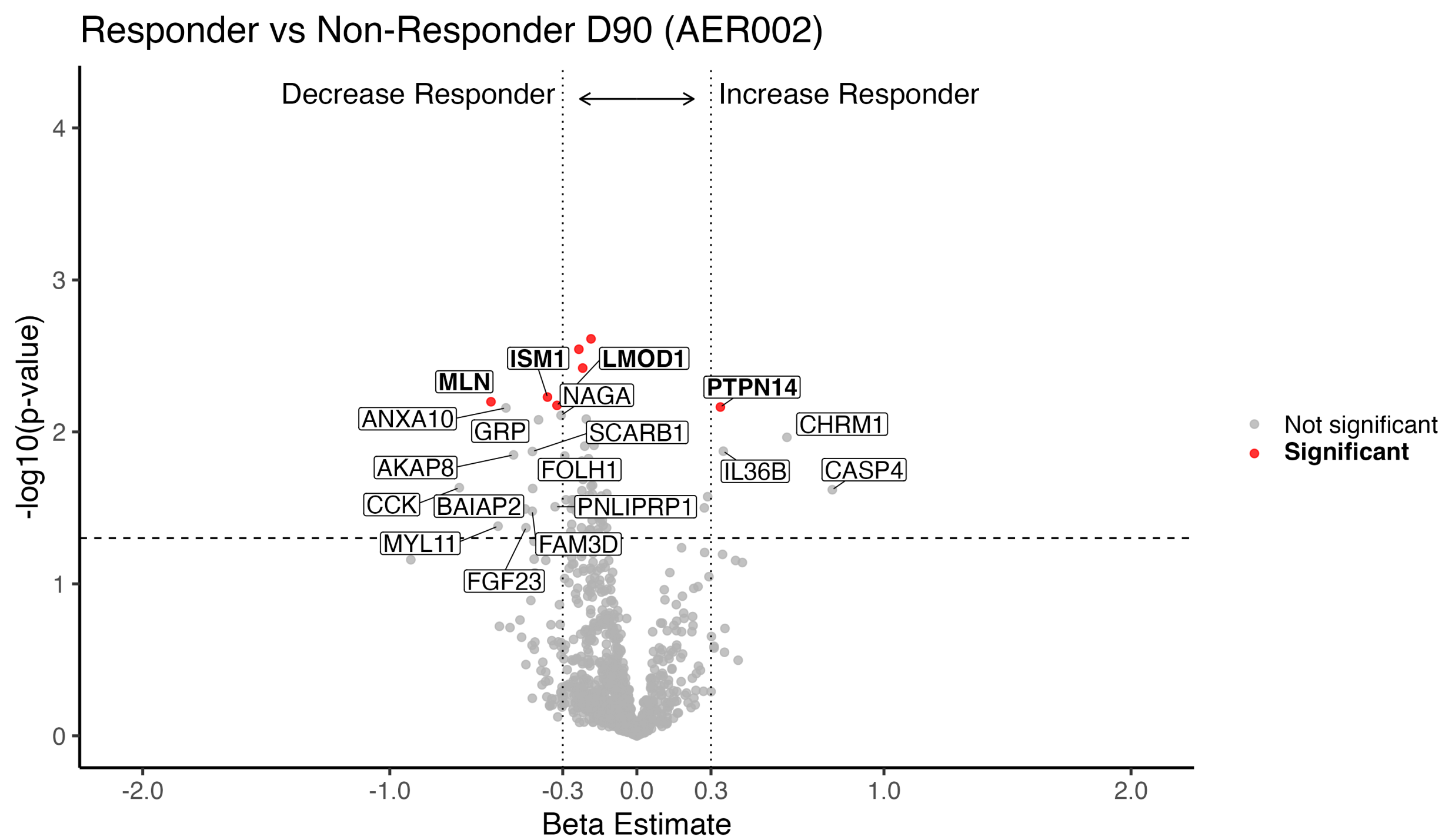

Supplementary Figure 4. Plasma proteomics AER002 self-identified responder versus non-responder at D90. Volcano plots showing differential plasma protein expression for AER002 self-identified responders (n=10) versus non-responders (n=14) at D90. Differential expression was assessed using protein-specific linear mixed-effects models. All tests were two-sided, and P values were adjusted for multiple comparisons using the Storey q-value method. Significant proteins with q-value < 0.05 are shown in bold red. The horizontal dashed line indicates the unadjusted P-value threshold of 0.05. Vertical dashed lines indicate beta estimates of −0.3 and 0.3.

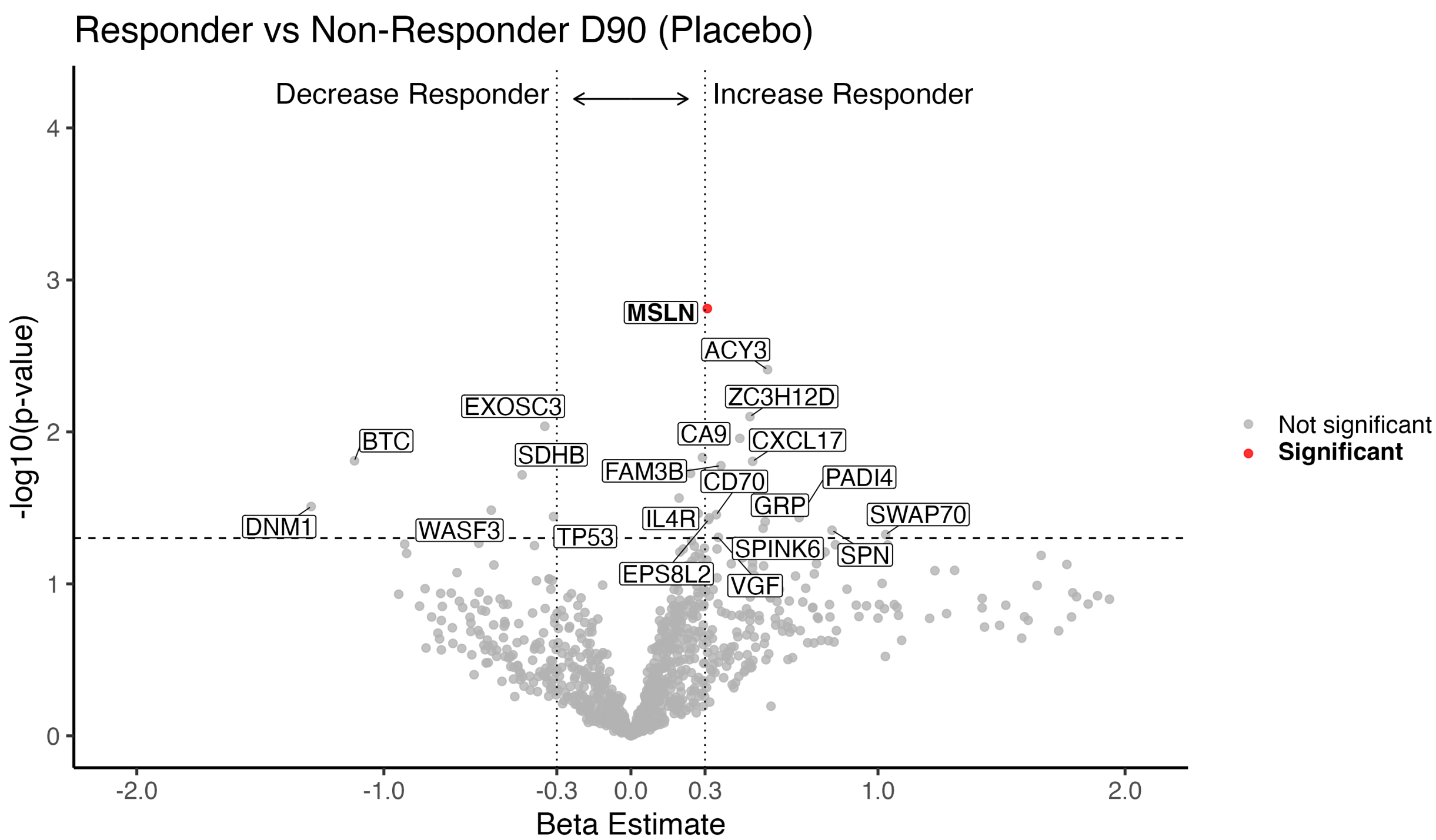

Supplementary Figure 5. Plasma proteomics placebo self-identified responder versus non-responder at D90. Volcano plots showing differential plasma protein expression for placebo self-identified responders (n=6) versus non-responders (n=6) at D90. Differential expression was assessed using protein-specific linear mixed-effects models. All tests were two-sided, and P values were adjusted for multiple comparisons using the Storey q-value method. Significant proteins with q-value < 0.05 are shown in bold red. The horizontal dashed line indicates the unadjusted P-value threshold of 0.05. Vertical dashed lines indicate beta estimates of −0.3 and 0.3.

UNIVERSITY OF CALIFORNIA, SAN FRANCISCO

Clinical Research Protocol

AN EXPLORATORY, RANDOMIZED, DOUBLE-BLIND PLACEBO-CONTROLLED STUDY TO ASSESS THE SAFETY OF AN ANTI-SARS-COV-2 MONOCLONAL ANTIBODY AND RESPONSE TO TREATMENT IN INDIVIDUALS WITH LONG COVID (OUTSMART-LC) (COVID-19)

Protocol Number:	23-38629
Version Date:	2023-NOV-16
Investigational Product:	AER002
IND Number:	[redacted]
Development Phase:	Exploratory
Sponsor:	University of California, San Francisco
Funding Organization:	University of California, San Francisco<br>Aerium Therapeutics
Principal Investigator:	Name: Michael Peluso, MD<br>Telephone: [redacted]<br>E-mail: michael.peluso@ucsf.edu
Safety Monitoring Committee Chair:	Name: Vincent (Vince) Marconi, MD<br>Telephone: [redacted]<br>E-mail: [redacted]

Approval:

PI or Sponsor Signature (Name and Title) *Date*

This confidential information about an investigational product is provided for the exclusive use of investigators of this product and is subject to recall at any time. The information in this document may not be disclosed unless federal or state law or regulations require such disclosure. Subject to the foregoing, this information may be disclosed only to those persons involved in the study who have a need to know, with the obligation not to further disseminate this information.

PROTOCOL AGREEMENT

I have read the protocol specified below. In my formal capacity as Investigator, my duties include ensuring the safety of the study subjects enrolled under my supervision and providing University of California, San Francisco with complete and timely information, as outlined in the protocol. It is understood that all information pertaining to the study will be held strictly confidential and that this confidentiality requirement applies to all study staff at this site. Furthermore, on behalf of the study staff and myself, I agree to maintain the procedures required to carry out the study in accordance with accepted GCP principles and to abide by the terms of this protocol.

Protocol Number: 23-38629

Protocol Title: An Exploratory, Randomized, Double-Blind Placebo-Controlled Study to Assess the Safety of an Anti-SARS-CoV-2 Monoclonal Antibody and Response to Treatment in Individuals with Long COVID (outSMART-LC) (COVID-19)

Protocol Date: 2023-NOV-16

____ __________________

Investigator Signature *Date*

____

Print Name and Title

Site # ____________

Site Name ____

Address ____

____

____

____

Phone Number ____

TABLE OF CONTENTS

1 BACKGROUND 13
1.1 Overview of Non-Clinical Studies 13
1.2 Overview of Clinical Studies 13
2 STUDY RATIONALE 14
2.1 Hypothesis 15
2.2 Program Overview (LIINC Cohort) 15
2.3 Risk / Benefit Assessment 16
3 STUDY OBJECTIVES 18
3.1 Primary Objective 18
3.2 Secondary Objectives 18
4 STUDY DESIGN 18
4.1 Study Overview 18
5 CRITERIA FOR EVALUATION 18
5.1 Efficacy Endpoints 18
5.2 Safety Evaluations 19
5.3 Other Evaluations 19
6 SUBJECT SELECTION 19
6.1 Study Population 19
6.2 Inclusion Criteria 19
6.3 Exclusion Criteria 21
7 CONCURRENT MEDICATIONS 22
7.1 Allowed Medications and Treatments 22
8 STUDY TREATMENTS 23
8.1 Method of Assigning Subjects to Treatment Groups 23
8.2 Blinding 23
8.3 Formulation of Test and Control Products 23
8.4 Supply of Study Drug at the Site 23
8.5 Supply of Study Drug at the Site 24
8.6 Study Drug Accountability 24
8.7 Measures of Treatment Compliance 24
9 STUDY PROCEDURES AND GUIDELINES 24
9.1 Clinical Assessments 25
9.2 Clinical Laboratory Measurements 29
9.3 Research Laboratory Measurements 30
9.4 Biospecimens 31
10 EVALUATIONS BY VISIT 31
10.1 Screening Visit (D-28) 31
10.2 Baseline Evaluation (D-7) 32
10.3 Intervention (D0) 32

10.4 Telephone Follow-up (Phone Follow-Up, D1) 33
10.5 Follow-up Visits (D15, D30, D90, D180, D360) 33
10.6 Unscheduled (Interim) Visits 33
10.7 Study Discontinuation Visit 33
11 ADVERSE EXPERIENCE REPORTING AND DOCUMENTATION 33
11.1 Adverse Events 33
11.2 Serious Adverse Experiences (SAE) or Serious SAR 35
11.3 Safety Monitoring Committee Chair / Medical Monitoring 36
12 DISCONTINUATION AND REPLACEMENT OF SUBJECTS 36
12.1 Early Discontinuation 36
12.2 Withdrawal of Subjects from the Study 37
12.3 Replacement of Subjects 37
12.4 Lost to Follow Up 37
13 PROTOCOL VIOLATIONS 37
14 DATA SAFETY MONITORING 38
15 STATISTICAL METHODS AND CONSIDERATIONS 38
15.1 Data Sets Analyzed 38
15.2 Demographic and Baseline Characteristics 39
15.3 Analysis of Primary Endpoint 39
15.4 Analysis of Secondary Endpoints 39
15.5 Interim Analysis 39
15.6 Sample Size and Randomization 39
16 DATA COLLECTION, RETENTION AND MONITORING 40
16.1 Data Collection Instruments 40
16.2 Data Management Procedures 40
16.3 Data Quality Control and Reporting 40
16.4 Archival of Data 40
16.5 Availability and Retention of Investigational Records 40
16.6 Monitoring 41
16.7 Subject Confidentiality 41
17 ADMINISTRATIVE, ETHICAL, REGULATORY CONSIDERATIONS 41
17.1 Protocol Amendments 41
17.2 Institutional Review Boards and Independent Ethics Committees 41
17.3 Informed Consent Form 42
17.4 Publications 42
17.5 Investigator Responsibilities 42
18 REFERENCES 43

LIST OF ABBREVIATIONS

ADA	Anti-drug antibody
AE	Adverse Event
ALT	Alanine Aminotransferase
AST	Aspartate Aminotransferase
BUN	Blood Urea Nitrogen
CFR	Code Of Federal Regulations
COVID-19	Coronavirus Disease 2019
CRF	Case Report Form
CRP	C-Reactive Protein
DMC	Data Monitoring Committee
DSMB	Data Safety Monitoring Board
ESR	Erythrocyte Sedimentation Rate
FDA	Food And Drug Administration
GCP	Good Clinical Practice
HIPAA	Health Insurance Portability and Accountability Act Of 1996
ICF	Informed Consent Form
ICH	International Conference on Harmonisation
IEC	Independent Ethics Committee
IRB	Institutional Review Board
IV	Intravenous
LIINC	Long-term Impact of Infection with Novel Coronavirus
mAb	Monoclonal Antibody
mEq	Milliequivalent
mITT	Modified Intention-to-Treat
PD	Pharmacodynamic
PI	Principal Investigator
PK	Pharmacokinetic
PRN	Pro Re Nata
SAE	Serious Adverse Experience
SAP	Statistical Analysis Plan
SAR	Suspected Adverse Reaction
SARS-CoV-2	Severe Acute Respiratory Syndrome Coronavirus 2
SD	Standard Deviation
SMC	Safety Monitoring Committee

PROTOCOL SYNOPSIS

**TITLE**	AN EXPLORATORY, RANDOMIZED, DOUBLE-BLIND PLACEBO-CONTROLLED STUDY TO ASSESS THE SAFETY OF AN ANTI-SARS-COV-2 MONOCLONAL ANTIBODY AND RESPONSE TO TREATMENT IN INDIVIDUALS WITH LONG COVID (OUTSMART-LC) (COVID-19)
**SPONSOR**	University of California, San Francisco
**FUNDING ORGANIZATION**	University of California, San Francisco<br>Aerium Therapeutics
**NUMBER OF SITES**	1
**RATIONALE**	There are currently no widely accepted therapies for Long COVID. Viral antigen persistence is one of the key mechanisms that might drive this condition. Clearance of viral antigen or infected cells with a SARS-CoV-2 monoclonal antibody may lead to symptomatic improvement in people with Long COVID.
**STUDY DESIGN**	Randomized, double-blind, placebo-controlled study.
**PRIMARY OBJECTIVE**	To evaluate the safety and tolerability of AER002 in individuals who are experiencing Long COVID.
**SECONDARY OBJECTIVES**	1. Describe the ability of AER002 to affect virologic markers of chronic SARS-CoV-2 infection.<br>2. Describe the ability of AER002 to improve PASC outcomes.<br>3. Describe the ability of AER002 to reduce post-COVID inflammation.
**NUMBER OF SUBJECTS**	30
**SUBJECT SELECTION CRITERIA**	Inclusion Criteria:<br>1. Male, female, or transgender ≥18 years of age at Screening.<br>2. Enrolled or willing to enroll and complete at least 1 visit in the UCSF Long-term Impact of Infection with Novel Coronavirus (LIINC) study. Any adult who has been infected with SARS-CoV-2 or has ever received or is eligible to receive a SARS-CoV-2 vaccination, does not have chronic anemia, and who is able to provide written informed consent, is eligible to participate in LIINC.<br>3. History of confirmed acute SARS-CoV-2 infection, whose initial infection meets criteria as outlined below:<br>a. Report of a positive nucleic acid amplification test (NAAT) and/or a positive SARS-Cov-2 antigen rapid diagnostic test (RDT). Written proof of the test will be

	requested but is not required as long as the participant attests to the positive test.<br><br>AND<br><br>b. Long COVID (see below) attributed to SARS-CoV-2 infection with a variant against which AER002 is known to have neutralizing activity. In cases in which the variant is not known from prior viral sequencing (most cases), the SARS-CoV-2 infection to which Long COVID is attributed should have been dated prior to August 15, 2022.<br><br>Note: For individuals who may be chronically infected (with persistent viral shedding from nasopharynx), a genotype is required to confirm that they have a AER002-susceptible variant.<br><br>4. Clinical evidence of Long COVID, as confirmed by the investigator's assessment.<br><br>a. At least two symptoms (see list) that are new or worsened since the time of SARS-CoV-2 infection, not known to be attributable to another cause upon assessment by the PI. At least two symptoms from those listed here must be present: systemic symptoms (e.g., fatigue, chills, post-exertional malaise), neurocognitive symptoms (e.g., trouble with memory/concentration ("brain fog"), headache, dysautonomia/postural orthostatic tachycardia syndrome, dizziness, unsteadiness, neuropathy, sleep disturbance), cardiopulmonary symptoms (e.g., chest pain, palpitations, shortness of breath, cough, fainting spells), musculoskeletal symptoms (e.g., muscle aches, joint pain), gastrointestinal symptoms (e.g., nausea, diarrhea). Although other symptoms (e.g., skin rash, hair loss, mental health symptoms, trouble with smell/taste, genitourinary symptoms) will be recorded and tracked, at least two core symptoms listed above must be present. Note: the two symptoms can be from within the same category (for example, brain fog and headache).<br><br>AND<br><br>b. Symptoms must have been present for at least 60 days prior to screening. Symptoms that wax and wane must have been initially present at least 60 days prior to screening.<br><br>AND<br><br>c. Symptoms must be reported to be at least somewhat bothersome and to have an impact on quality of life and/or everyday functioning.

	5. Long COVID attributed to a SARS-CoV-2 infection prior to August 15, 2022. Note: While individuals re-infected with SARS-CoV-2 after August 15, 2022 will not be excluded, the SARS-CoV-2 infection after which Long COVID symptoms began must pre-date August 15, 2022.<br>6. Not currently hospitalized.<br>7. Body mass index (BMI) 18 to 50 kilograms/meter squared (kg/m2), inclusive, at the time of screening.<br>8. In otherwise stable health, as assessed by the investigator within 28 days prior to Screen, based on medical history, physical assessment, laboratory findings, and vital signs.<br>9. Participants who are of childbearing potential (CBP) and male participants with sexual partner(s) who are females of CBP must agree to use adequate contraception from study consent through 360 days after dosing. Adequate contraception is defined as using hormonal contraceptives or an intrauterine device combined with at least 1 of the following forms of contraception: a diaphragm, a cervical cap, or a condom. Total abstinence from vaginal intercourse, in accordance with the lifestyle of the participant, is also acceptable.<br>10. Willingness and ability to comply with the study protocol. This includes reliable transportation and sufficient time to attend all visits.<br>11. Written informed consent (and assent when applicable) obtained from subject or subject's legal representative and ability for subject to comply with the requirements of the study.<br><u>Exclusion Criteria</u>:<br>1. Current acute SARS-CoV-2 infection at the time of screening.<br>2. Long COVID attributed to a SARS-CoV-2 infection after August 15, 2022.<br>3. Previously received treatment or prophylaxis with a SARS-CoV-2-specific mAb, or plan to receive such treatment before exiting the study.<br>4. Previously received COVID-19 convalescent plasma treatment within 60 days prior to planned Day 0 or plan to receive such treatment before exiting the study.<br>5. Plans to receive any investigational or approved vaccine or booster for SARS-CoV-2 within 60 days prior to planned Day 0 or before Day 30 following planned Day 0.<br>6. Active cardiovascular disease, defined as known prior:<br>a. Myocardial infarction within 90 days of screening<br>OR<br>b. Coronary artery bypass within 90 days of screening

	OR<br>c. Current heart failure with reduced ejection fraction (<45%)<br>OR<br>d. Current pulmonary arterial hypertension.<br>7. Known stroke within 3 months prior to planned Day 0.<br>8. Known active bacterial, fungal, viral, or other infection besides SARS-CoV-2 requiring treatment within the 28 days prior to planned Day 0 and meeting criteria for systemic involvement upon review by the PI. Note: Mild or limited infections such as uncomplicated urinary tract or yeast infections, sexually transmitted infections, and mild dermatophyte infections may be reviewed with the medical monitor but are not exclusionary.<br>9. Major surgery within 6 months prior to planned Day 0 or planned major surgery during the first 180 days following planned Day 0.<br>10. History of unplanned hospitalization for >24 hours within 28 days prior to Screening.<br>11. Active Hepatitis B (Hep B) infection (defined as Hep B surface antigen (sAg) positive). Note: A known positive Hep B core antibody (cAb) in the absence of positive sAg is not considered exclusionary.<br>12. Active Hepatitis C (Hep C) infection (defined as Hep C Ab positive or indeterminate with detectable Hep C RNA). Note: Those with cured Hep C (Ab positive or indeterminate but negative Hep C RNA) will remain eligible.<br>13. HIV infection that is known to be unstable (two or more consecutive plasma HIV RNA values >48 copies/mL in the 6 months prior to screen) or uncontrolled (not on antiretroviral therapy (ART)). In addition, people with HIV who have a current CD4+ T cell count < 200 cells/uL, a history of AIDS defining illness without immune reconstitution, or clinical manifestations of symptomatic HIV will be excluded.<br>14. Severe coagulopathy that would prevent an infusion (international normalized ratio ((INR) >2.0, history of hemophilia).<br>15. Severe anemia (hemoglobin <9 grams/deciliter (g/dL)).<br>16. Moderate or severe immunocompromise, according to the current NIH COVID-19 Treatment Guidelines as of March 6, 2023. The detailed list is in Appendix 2, and includes the following: (a) receiving active treatment for solid tumor or hematologic malignancy, including use of systemic chemotherapy for treatment of cancer within the year prior to screening, (b) prior solid-organ transplant with active immunosuppressive therapy, (c) CAR-T cell therapy or hematopoietic cell transplant, on immunosuppressive therapy or

	transplant within the prior 2 years, (d) primary immunodeficiency syndromes, (e) advanced or untreated HIV infection (see above), (f) on active high-dose corticosteroids (i.e., >= 20mg prednisone or equivalent daily per day for >= 2 weeks).<br>17. Known prior diagnosis of myalgic encephalomyelitis/chronic fatigue syndrome (ME/CFS), preceding and not related to SARS-CoV-2 infection and not worsened since SARS-CoV-2 infection.<br>18. Known prior diagnosis of dysautonomia, preceding and not related to SARS-CoV-2 infection and not worsened since SARS-CoV-2 infection.<br>19. History of anaphylaxis or hypersensitivity upon receiving IV antibody infusions in the past.<br>20. Known allergy to any components used in the formulation of the intervention.<br>21. History of anaphylaxis or similar significant allergic reaction to prescription or non-prescription drugs or food products. Similarly, presence of severe atopic conditions as assessed by the PI represents significant risk for allergic reaction.<br>22. Pregnant, breastfeeding, or unwilling to practice birth control abide by the contraception requirements outlined in the inclusion criteria.<br>23. Participation in a clinical trial with receipt of an investigational product within 28 days or 5 half-lives (whichever is longer) prior to planned Day 0. For PET tracers specifically, which have a short half-life and are not biologically active outside this window, elapse of 5 half-lives prior to the planned D0 infusion is sufficient.<br>24. Current alcohol or illicit drug use as determined by the investigator to preclude participation.<br>25. Presence of a condition or abnormality that in the opinion of the Investigator would compromise the safety of the patient or the quality of the data.<br>26. Study site personnel directly affiliated with the study or family of directly involved personnel.<br>27. History of cytokine release syndrome (CRS) secondary to infection and/or medication.
**TEST PRODUCT, DOSE, AND ROUTE OF ADMINISTRATION**	AER002 1200mg, IV once
**CONTROL PRODUCT, DOSE AND ROUTE OF ADMINISTRATION**	Placebo, IV once

**DURATION OF SUBJECT PARTICIPATION AND DURATION OF STUDY**	Subjects will be on study for up to 1.25 years<br>Screening: up to 28 days<br>Treatment: 1 day<br>Follow-up: 360 days
**CONCOMMITANT MEDICATIONS**	All concomitant medication and concurrent therapies taken within 28 days prior to Screening will be documented. Dose, route, unit frequency of administration, and indication for administration and actual or estimated dates of medication will be captured.<br>Details regarding all SARS-CoV-2 vaccinations and/or boosters, as well as COVID-specific therapies, will be collected.
**EFFICACY EVALUATIONS**	• The change in patient-reported outcomes (PROs) PROMIS-29 score from Baseline to D90.
PRIMARY ENDPOINT	• Safety and tolerability.
SECONDARY ENDPOINTS	• Change in other assessments (EuroQoL Quality of Life, neurocognitive assessment (e.g., NIH Toolbox), DASI, dysautonomia assessment (e.g., COMPASS-31), DSQ-PEM, 6MWT performance) between Baseline and at D30, D90, D180, and D360.
**OTHER EVALUATIONS**	• Percentage of participants with no detection of SARS-CoV-2 plasma remnants (i.e., viral detection by reverse transcriptase-polymerase chain reaction ((RT-PCR) and Spike protein fragments) compared to baseline from 1 month post administration through 6 months post administration.<br>• Proportion with reduction in inflammatory markers (e.g., interleukin-6 (IL-6), tumor necrosis factor alpha (TNF-alpha) at Baseline and at D30, D90, D180, and D360.<br>• PK and PD analyses.
**SAFETY EVALUATIONS**	Number of participants with adverse events (AE)s (Treatment Emergent Adverse Events (TEAE)s, Serious Adverse Events (SAE)s, and Adverse Events of Special Interest (AESI)). The primary outcome will be the number of individuals in each group experiencing a severe (Grade 3 or greater) AE thought to be possibly, probably, or definitely related to study treatment throughout the study duration.
**PLANNED INTERIM ANALYSES**	Approximately 4 months after enrollment of the first participant and then every 4 months thereafter, the SMC will meet and review accrual (including screening and enrollment), AE summaries, including all reported Grade ≥3 AEs, retention of participants including off-study rates.<br>Serious adverse events will be monitored by the committee on an ongoing basis throughout the study.

**STATISTICS**<br>**Primary Analysis Plan**	Eligible patients who are randomized into the study and receive the study drug will comprise the modified intent to treat (mITT) population and will be the primary analysis population for all analyses.<br><br>Safety and tolerability data will be summarized by treatment group.<br><br>Adverse event rates will be coded by body system. Adverse events will be tabulated by treatment group and will include the number of patients for whom the event occurred, the rate of occurrence, and the severity and relationship to study drug. Adverse events between groups will be summarized by proportions with associated 95% Clopper-Pearson confidence intervals and will be compared using a two-sided 0.05 level Fisher exact test.
**Rationale for Number of Subjects**	This is an exploratory study, and the primary results will be descriptive. We will use AER002 as a probe to better understand LC biology and determine whether a signal is present to warrant further study.<br><br>For PROMIS-29, assuming a standard deviation (SD) of the within-person difference of 10, then the planned sample size will have 80% power to detect a difference of 11.2 on a two-sided 0.05 level test.<br><br>For biomarkers, such as IL-6, we will be able to detect a difference from a mean value of 2.5 pg/mL to 1.1 times the within-person standard deviation with 80% power.

1 BACKGROUND

Coronavirus disease 2019 (COVID-19) is caused by Severe Acute Respiratory Coronavirus type 2 (SARS-CoV-2), a ribonucleic acid (RNA) virus which emerged in Wuhan, China, in December 2019 and spread across the world at an unprecedented pace through human-to-human transmission. The spike protein of the 2019 novel coronavirus is a surface protein which binds to angiotensin-converting enzyme-2 (ACE-2) on human cells. The S1 subunit catalyzes attachment to ACE-2 and the S2 subunit allows fusion with cell membranes and subsequent entry into the cell. As a result, the spike protein is a relevant target for drug development. Moreover, antibodies (Abs) against the spike protein are able to neutralize the virus, prevent infection, and reduce the severity of disease.

The COVID-19 pandemic has resulted in a growing population of individuals recovering from SARS-CoV-2 infection. Approximately one in five American adults who have had COVID-19 still have symptoms of Long COVID (LC), a type of post-acute sequelae of SARS-CoV-2 infection (PASC).[1] Some aspects of this recovery may be unique to COVID-19, but many appear to be similar to recovery from other viral illnesses, critical illness, and/or sepsis.[2,3] LC is comprised of a broad range of symptoms that develop during or after COVID-19, continue for ≥2 months (i.e., three months from the onset of illness), have an impact on the patient's life, and are not explained by an alternative diagnosis. Consensus around the clinical definition of LC has progressed significantly, and as of October 1, 2021, there has been an International Classification of Diseases, Tenth Revision, Clinical Modification, (ICD-10), for unspecified post-COVID conditions (U09.9). The World Health Organization (WHO) has also created a global COVID-19 clinical platform CRF for clinicians and patients to collect and report information.[4] The United States (US) Department of Health and Human Services (HHS) and the Department of Justice (DoJ) released a guidance statement on LC as a disability under the Americans with Disabilities Act, the Rehabilitation Act of 1973, and the Patient Protection and Affordable Care Act.

Persistent viral infection with viral reservoirs and detection of circulating S protein after the initial acute illness is one potential pathogenic mechanism for Long COVID.[5] This mechanism may be able to be targeted by monoclonal antibodies (mAbs).[6] Therefore, this trial will study the safety and efficacy of AER002 to treat individuals with persistent infection and LC in an adult population.

1.1 Overview of Non-Clinical Studies

[redacted]

[redacted]

1.2 Overview of Clinical Studies

[redacted]

[redacted]

2 STUDY RATIONALE

Early in the pandemic, the common assumption was that SARS-CoV-2 would prove to be a transient infection, as is the case with coronaviruses in general. This assumption was challenged by early reports that viral nucleic acid and proteins could be detected in the gut mucosa months after infection.[8] Case reports emerged indicating that immunocompromised individuals including those with advanced malignancy, Human immunodeficiency virus (HIV)/Acquired Immunodeficiency Syndrome (AIDS), or on immunosuppression for autoimmune conditions, can harbor active replicating virus for many months.[9–11] More recently, similar observations have been made in immunocompetent people.[12–15] Autopsy studies of people post-COVID dying from related or unrelated reasons also began to report presence of viral nucleic acid or protein in various tissues months after the infection was apparently cleared.[16] Another provocative study demonstrated SARS-CoV-2 in neonatal stool following remote maternal COVID-19.[17] Taken together these studies provide growing support for SARS-CoV-2 persistence.

SARS-CoV-2 can infect several cell types and in theory any infected cell is at risk of harboring persistent virus. The precise localization of SARS-CoV-2 persistence is unknown, but it is widely assumed that this occurs in tissues. This may or may not include immune-privileged sites as has been observed with other ribonucleic acid (RNA) viruses (e.g. Ebola).[18] Because tissue studies are generally impractical due to invasiveness of these procedures, there are now efforts to develop less-invasive measures to assess this. Some studies identified detectable SARS-CoV-2 RNA in plasma and stool during the early post-acute phase.[19] One recent study found that a large proportion of individuals with LC had at least intermittently detectable circulating antigen in the plasma for up to a year post-infection.[20] However, it remains unclear whether antigen can also persist in asymptomatic individuals, whether what is being detected represents remnants of a long extinguished infection or ongoing virus production from a long-lived reservoir or ongoing replication.

The presence of viral RNA and proteins in the post-acute phase does not necessarily represent ongoing viral replication and viral RNA can persist even in the absence of culturable virus. Viral persistence represents a spectrum which includes the persistence of viral proteins, degraded or partial RNA fragments, and full-length replication-competent RNA. While each of these components could

stimulate immune responses, the optimal biological target is dependent on which components are driving the disease process. For example, if the presence of SARS-CoV-2 antigen represents active viral replication, targeting this with antivirals such as protease or RNA polymerase inhibition would be an effective approach. However, these agents have several disadvantages including complex administration, drug-drug interactions, and unattractive adverse effect profiles. Furthermore, if no active viral replication is occurring and only sub genomic RNA or protein is present, these therapies would not be effective. Instead, mAbs which act by neutralizing the spike protein of SARS-CoV-2 might be more attractive. By binding spike, which exists on the cell surface and can sometimes be detected in plasma of those with LC, these Abs could have beneficial effects through neutralization of proteins and virions, opsonization of virions and infected cells, and potentially the induction of Ab- or complement-mediated cellular cytotoxicity if viral proteins are expressed on the cell surface.[21] These agents typically have more favorable dosing and side effect profiles, and have the added benefit of clearing infected cells.

The mechanism of Long COVID remains incompletely understood, but multiple studies have suggested that the root cause is an inadequate immune response to SARS-CoV-2 resulting in virus persistence and either direct tissue damage or the indirect effects of chronic virus-mediated chronic inflammation. A corollary of this model is that those who mount an effective response will clear virus and return to normal health. Our primary hypothesis, therefore, is that the immune response to the virus in people with Long COVID needs to be addressed therapeutically. A neutralizing or non-neutralizing SARS-CoV-2 antibody response is almost certainly central to ensuring that the virus is cleared. A number of studies have suggested that those with Long COVID may be less likely to have mounted or maintained a humoral immune response; even in circumstances in which an individual has a robust level of circulating antibodies, the quality and activity of these antibodies could be inadequate to clear antigen. For these reasons, we believe that there is enough equipoise and data suggestive of potential benefit for monoclonal antibodies to warrant further study.

2.1 Hypothesis

We hypothesize that tissue viral persistence contributes to LC pathophysiology, and that administration of AER002 to individuals experiencing LC will result in symptom reduction as well as improvement in biomarkers of inflammation in comparison to those receiving placebo.

2.2 Program Overview (LIINC Cohort)

This study will occur within the Long-term Impact of Infection with Novel Coronavirus (LIINC) cohort,[22] an observational study of post-COVID conditions that has been ongoing at UCSF since April 2020. Since that time, LIINC has been characterizing the natural history and biology of LC. The cohort has recruited over 700 individuals, of whom over 350 report mild-to-severe LC symptoms. The retention rate at 1 year is 89%. Individuals are evaluated at scheduled visits and biological specimens (plasma, serum, peripheral blood mononuclear cells (PBMCs)) are collected and stored. A subset of the cohort is referred for additional studies including advanced cardiopulmonary (echocardiogram, cardiopulmonary exercise testing, cardiac magnetic resonance imaging (MRI), pulmonary function tests, and tilt table tests) neurologic (neuropsychiatric testing, lumbar puncture), imaging (fluorodeoxyglucose-positron emission tomography/computed tomography (FDG-PET/CT), and tissue assessment (gut biopsy, lymph node fine needle aspiration). To date, the LIINC team has conducted over 3000 participant-encounters and have enrolled over 200 individuals into the more intensive protocols. LIINC has also described the relationship between LC and adaptive immune responses,[23] inflammation,[24–27] autoimmunity,[28,29] microbial translocation,[30] neurocognitive changes,[31] and cardiopulmonary physiology.[26] The LIINC team has for decades conducted investigator-initiated clinical trials as part of the associated HIV program (SCOPE) and has the capacity to implement the study described in this protocol using established procedures.

Potential participants will be required to enroll in LIINC and to complete at least one visit to assess LC symptoms. As described below, we will take into account blood volumes from LIINC and outSMART-LC to stay within Red Cross guidelines. The trial participants will be recruited directly from LIINC if they have had LC subsequent to an acute COVID episode that occurred prior to August 15th, 2022. This cut-off is based upon the period of time when variants susceptible to AER002 were in predominant circulation in California (https://covariants.org/per-country). Subsequent to August 15th, 2022, Omicron lineages emerged (BQ.1.1 and XBB sub-lineages) which would not be susceptible to AER002 and which would therefore confound the interpretability of this proof-of-concept trial.

2.3 Risk / Benefit Assessment

The potential benefits to participants and society are likely to outweigh the minimal risks from participation in the study.

2.3.1 Potential Risks

Confidentiality. Participation in research may involve loss of privacy. Participants' records will be handled as confidentially as possible. All research records will be coded with a four-digit study identification (ID) code. Only the study investigators and their staff will have access to study records and test results. Study charts will be kept in a locked file cabinet in a locked office. Electronic data will be protected with a password and kept on a secure network. All collaborators will receive specimens only identified by the four-digit study ID. No individual identities will be used in any reports or publications resulting from this study.

Phlebotomy. Drawing blood from a vein may cause some discomfort, bleeding, or bruising where the needle enters the skin, and rarely, fainting or infection may occur. No more than 500 mL (2 cups) of blood will be drawn over any two-month period. This is within Red Cross guidelines. The total amount of blood allowable will take into account blood drawn as part of both LIINC and outSMART-LC. Risks of blood collection include anemia (low blood counts). Symptoms of anemia include tiredness, weakness, and dizziness. Participants will be checked for signs and symptoms of anemia at each visit, and CBC/diff will be checked as outlined in the schedule of events. If the investigator feels that a participant is at significant risk for anemia, the amount of blood collected will be reduced.

Nasal or oral swab. These collections may cause temporary discomfort.

Viral testing. Testing for viruses such as SARS-CoV-2, HIV and viral hepatitis and testing may be performed as part of this study. Being tested for these infections may cause anxiety, regardless of the test results. Receiving a positive test result may cause a lot of anxiety. If the test result is negative, there is still the possibility that a participant could be infected and test positive at some time in the future. In addition, there is always the rare possibility that the test results could be wrong. Newly positive test results for SARS-CoV-2, HIV, hepatitis B, hepatitis C, and Tuberculosis (TB) infections are required to be reported to the Department of Public Health.

Drug side effects. Possible side effects may include:

1) Allergic reactions. Allergic/hypersensitivity reactions can happen during and after infusion. Participants may experience the following signs and symptoms of allergic reactions: fever, chills, nausea, headache, shortness of breath (SOB), low or high blood pressure (BP), rapid or slow heart rate (HR), chest discomfort or pain, weakness, confusion, feeling tired, wheezing, swelling of lips, face, or throat, rash including hives, itching, muscle aches, feeling faint, dizziness and sweating. These reactions may be severe or life threatening. For this reason, participants will be monitored for at least 120 minutes after the delivery of AER002 vs placebo on Day 0.

2) Cytokine Release Syndrome (CRS). CRS is an acute systemic hyperinflammatory syndrome that can occur during and after infusion. Participants may experience the following symptoms:

fever, chills, nausea, vomiting, fatigue, body aches, headache, cough, low BP, skin rash, confusion, delirium, edema, SOB, rapid or slow HR, chest pain or discomfort, weakness, difficulty swallowing, diarrhea, dizziness, and joint pain. These reactions may be severe or life threatening. For this reason, participants will be monitored for at least 120 minutes after the delivery of AER002 vs placebo on Day 0.

3) Worsening symptoms after treatment. Participants may experience new or worsening symptoms after infusion, including fever, difficulty breathing, rapid or slow heart rate, tiredness, weakness, or confusion.

4) Local infusion-related site reactions. The side effects of getting any medicine by vein (IV infusion) may include brief pain, erythema, burning, itching, tingling, stinging, bleeding, bruising of the skin, soreness, swelling, skin necrosis, and possible infection at the infusion site.

5) Unanticipated AEs. These are not all the possible side effects.

2.3.2 Protection Against Risk

Confidentiality. Participants' records will be handled as confidentially as possible. Participants will be assigned a unique four-digit study ID code that will appear on all specimens and in our database. All biologic specimens and clinical data obtained from this study will be linked to this code and not to personal identifying information (e.g., name, social security number, medical record number). A key which will link the four-digit code to the personal information will be maintained on a secure server only accessible to designated study staff and maintained by the PI. Lab personnel and database programmers will have access only to the coded number and the participant's date of birth; no other personal identification information will be available to them. Collaborators will receive specimens only identified by the four-digit study ID. No individual identities will be used in any reports or publications resulting from this study.

Phlebotomy. Relevant personnel are trained and certified phlebotomists. We will stay within Red Cross Guidelines of less than 500 mL every two months. Participants will be checked for signs and symptoms of anemia at each visit, and CBC/diff will be checked as outlined in the schedule of events. If the investigator feels that an individual is at significant risk for anemia, the amount of blood collected will be reduced. If the study participant's hemoglobin falls below 9 grams/deciliter (g/dl) or HCT falls below 27%, we will draw 5 mL of blood to check safety labs such as Hgb and HCT. Other than the blood required to check safety labs, the participant will not have more blood drawn until the Hgb rises above 9 g/dl or the HCT rises above 27%.

2.3.3 Benefits to Participants

There is currently no accepted treatment for LC. Participants should not anticipate a personal benefit from participation in the study. However, it is possible that if the therapeutic intervention has the hypothesized effects, participants could experience relief from LC symptoms. Furthermore, it is possible that participants might benefit in general from engagement in research.

2.3.4 Benefits to Society

The identification of a treatment for LC symptoms could benefit the likely millions of individuals suffering from this condition.

3 STUDY OBJECTIVES

3.1 Primary Objective

The primary objective of the study is to evaluate the safety and tolerability of AER002 in individuals who are experiencing Long COVID.

3.2 Secondary Objectives

Secondary objectives include the following:

- Describe the ability of AER002 to affect virologic markers of chronic SARS-CoV-2 infection.
- Describe the ability of AER002 to improve PASC outcomes.
- Describe the ability of AER002 to reduce post-COVID inflammation.

4 STUDY DESIGN

4.1 Study Overview

This is an exploratory, 2:1 randomized, double-blind, placebo-controlled study to assess the safety and efficacy of AER002 to treat LC.

The study will enroll approximately 30 participants who meet the WHO LC criteria. Participants will be enrolled at a single center and randomized 2:1 to receive AER002 1200mg or placebo. Randomization will be stratified by duration of infection (<6 months and ≥6 months) at Day 0. Evaluations will take place at baseline and at timepoints up to 1-year post-infusion.

Screening data will be reviewed to determine subject eligibility. Subjects who meet all inclusion criteria and none of the exclusion criteria will be entered into the study.

The following treatment regimens will be used:

- AER002 1200mg
- Placebo

The total duration of subject participation will be approximately 12 months.

5 CRITERIA FOR EVALUATION

5.1 Efficacy Endpoints

- The change in patient-reported outcomes (PROs) PROMIS-29 score from Baseline to D90.
- Change PROMIS-29 score in those with symptoms at Baseline and at D30, D90, D180, and D360.
- Change in other assessments (EuroQoL Quality of Life, neurocognitive assessment (e.g., NIH Toolbox), DASI, dysautonomia (e.g., COMPASS-31 or similar assessment), DSQ-PEM, 6MWT performance) between Baseline and at D30, D90, D180, and D360.
- Percentage of participants with no detection of SARS-CoV-2 plasma remnants (i.e., viral detection by reverse transcriptase-polymerase chain reaction (RT-PCR) and Spike protein fragments) compared to baseline from 1 month post administration through 6 months post administration.
- Proportion with reduction in inflammatory markers (e.g., interleukin-6 (IL-6), tumor necrosis factor alpha (TNF-alpha) at Baseline and at D30, D90, D180, and D360.

5.2 Safety Evaluations

- Number of participants with adverse events (AE)s (Treatment Emergent Adverse Events (TEAE)s, Serious Adverse Events (SAE)s, and Adverse Events of Special Interest (AESI)). The primary outcome will be the number of individuals in each group experiencing a severe (Grade 3 or greater) AE thought to be possibly, probably, or definitely related to study treatment throughout the study duration. Specific AESI and their associated symptoms experienced with IV infusions include the following:
    - Immune-mediated AEs include Type I – Type IV hypersensitivity reactions. Types I-III occur within the first 24 hours of exposure and are considered immediate reactions. Type IV are considered delayed. They can occur up to 72 hours post exposure.
    - Type I hypersensitivity reactions manifest as anaphylaxis, urticaria, and angioedema.
    - Type II hypersensitivity reactions involve cell depletion and/or destruction without inflammation.
    - Type III hypersensitivity reactions involve immune complexes causing serum sickness type reactions.
    - Type IV hypersensitivity reactions are cell mediated inflammatory responses.
    - Cytokine release syndrome – may mimic anaphylaxis and present with hypotension, urticaria, and an overall hyperinflammatory response.

NOTE: None of the AESI have occurred with the study drug as part of the ongoing Phase 1 Clinical Trial COV-2022-001.

5.3 Other Evaluations

- Pharmacokinetic and pharmacodynamic analyses.

6 SUBJECT SELECTION

6.1 Study Population

Subjects with a history of SARS-CoV-2 infection who meet the case definition for Long COVID, and who meet the inclusion and exclusion criteria will be eligible for participation in this study.

6.2 Inclusion Criteria

1. Male, female, or transgender ≥18 years of age at Screening.
2. Enrolled or willing to enroll and complete at least 1 visit in the UCSF Long-term Impact of Infection with Novel Coronavirus (LIINC) study. Any adult who has been infected with SARS-CoV-2 or has ever received or is eligible to receive a SARS-CoV-2 vaccination, does not have chronic anemia, and who is able to provide written informed consent, is eligible to participate in LIINC.
3. History of confirmed acute SARS-CoV-2 infection, whose initial infection meets criteria as outlined below:
    a. Report of a positive nucleic acid amplification test (NAAT) and/or a positive SARS-Cov-2 antigen rapid diagnostic test (RDT). Written proof of the test will be requested but is not required as long as the participant attests to the positive test.

       AND

    b. Long COVID (see below) attributed to SARS-CoV-2 infection with a variant against which AER002 is known to have neutralizing activity. In cases in which the variant is not

known from prior viral sequencing (most cases), the SARS-CoV-2 infection to which Long COVID is attributed should have been dated prior to August 15, 2022.

Note: For individuals who may be chronically infected, a genotype is required to confirm that they have a AER002-susceptible variant.

4. Clinical evidence of Long COVID, as confirmed by the investigator's assessment.
    a. At least two symptoms (see list) that are new or worsened since the time of SARS-CoV-2 infection, not known to be attributable to another cause upon assessment by the PI. At least two symptoms from those listed here must be present: systemic symptoms (e.g., fatigue, chills, post-exertional malaise), neurocognitive symptoms (e.g., trouble with memory/concentration ("brain fog"), headache, dysautonomia/postural orthostatic tachycardia syndrome, dizziness, unsteadiness, neuropathy, sleep disturbance), cardiopulmonary symptoms (e.g., chest pain, palpitations, shortness of breath, cough, fainting spells), musculoskeletal symptoms (e.g., muscle aches, joint pain), gastrointestinal symptoms (e.g., nausea, diarrhea). Although other symptoms (e.g., skin rash, hair loss, mental health symptoms, trouble with smell/taste, genitourinary symptoms) will be recorded and tracked, at least two core symptoms listed above must be present. Note: the two symptoms can be from within the same category (for example, brain fog and headache).

       AND

    b. Symptoms must have been present for at least 60 days prior to screening. Symptoms that wax and wane must have been initially present at least 60 days prior to screening.

       AND

    c. Symptoms must be reported to be at least somewhat bothersome and to have an impact on quality of life and/or everyday functioning.
5. Long COVID attributed to a SARS-CoV-2 infection prior to August 15, 2022. Note: While individuals re-infected with SARS-CoV-2 after August 15, 2022 will not be excluded, the SARS-CoV-2 infection after which Long COVID symptoms began must pre-date August 15, 2022.
6. Not currently hospitalized.
7. Body mass index (BMI) 18 to 50 kilograms/meter squared (kg/m2), inclusive, at the time of screening.
8. In otherwise stable health, as assessed by the investigator within 28 days prior to Screen, based on medical history, physical assessment, laboratory findings, and vital signs.
9. Participants who are of childbearing potential (CBP) and male participants with sexual partner(s) who are females of CBP must agree to use adequate contraception from study consent through 360 days after dosing. Adequate contraception is defined as using hormonal contraceptives or an intrauterine device combined with at least 1 of the following forms of contraception: a diaphragm, a cervical cap, or a condom. Total abstinence from vaginal intercourse, in accordance with the lifestyle of the participant, is also acceptable.
10. Willingness and ability to comply with the study protocol. This includes reliable transportation and sufficient time to attend all visits.
11. Written informed consent (and assent when applicable) obtained from subject or subject's legal representative and ability for subject to comply with the requirements of the study.

6.3 Exclusion Criteria

1. Current acute SARS-CoV-2 infection at the time of screening.
2. Long COVID attributed to a SARS-CoV-2 infection after August 15, 2022.
3. Previously received treatment or prophylaxis with a SARS-CoV-2-specific mAb, or plan to receive such treatment before exiting the study.
4. Previously received COVID-19 convalescent plasma treatment within 60 days prior to planned Day 0 or plan to receive such treatment before exiting the study.
5. Plans to receive any investigational or approved vaccine or booster for SARS-CoV-2 within 60 days prior to planned Day 0 or before Day 30 following planned Day 0.
6. Active cardiovascular disease, defined as known prior:
    a. Myocardial infarction within 90 days of screening,

       OR
    b. Coronary artery bypass within 90 days of screening,

       OR
    c. Current heart failure with reduced ejection fraction (<45%)

       OR
    d. Current pulmonary arterial hypertension.
7. Known stroke within 3 months prior to planned Day 0.
8. Known active bacterial, fungal, viral, or other infection besides SARS-CoV-2 requiring treatment within the 28 days prior to planned Day 0 and meeting criteria for systemic involvement upon review by the PI. Note: Mild or limited infections such as uncomplicated urinary tract or yeast infections, sexually transmitted infections, and mild dermatophyte infections may be reviewed with the medical monitor but are not exclusionary.
9. Major surgery within 6 months prior to planned Day 0 or planned major surgery during the first 180 days following planned Day 0.
10. History of unplanned hospitalization for >24 hours within 28 days prior to Screening.
11. Active Hepatitis B (Hep B) infection (defined as Hep B surface antigen (sAg) positive). Note: A known positive Hep B core antibody (cAb) in the absence of positive sAg is not considered exclusionary.
12. Active Hepatitis C (Hep C) infection (defined as Hep C Ab positive or indeterminate with detectable Hep C RNA). Note: Those with cured Hep C (Ab positive or indeterminate but negative Hep C RNA) will remain eligible.
13. HIV infection that is known to be unstable (two or more consecutive plasma HIV RNA values >48 copies/mL in the 6 months prior to screen) or uncontrolled (not on antiretroviral therapy (ART)). In addition, people with HIV who have a current CD4+ T cell count < 200 cells/uL, a history of AIDS defining illness without immune reconstitution, or clinical manifestations of symptomatic HIV will be excluded.
14. Severe coagulopathy that would prevent an infusion (international normalized ratio ((INR) >2.0, history of hemophilia).
15. Severe anemia (hemoglobin <9 grams/deciliter (g/dL)).

16. Moderate or severe immunocompromise, according to the current NIH COVID-19 Treatment Guidelines as of March 6, 2023. The detailed list is in Appendix 2, and includes the following: (a) receiving active treatment for solid tumor or hematologic malignancy, including use of systemic chemotherapy for treatment of cancer within the year prior to screening, (b) prior solid-organ transplant with active immunosuppressive therapy, (c) CAR-T cell therapy or hematopoietic cell transplant, on immunosuppressive therapy or transplant within the prior 2 years, (d) primary immunodeficiency syndromes, (e) advanced or untreated HIV infection (see above), (f) on active high-dose corticosteroids (i.e., >= 20mg prednisone or equivalent daily per day for >= 2 weeks).
17. Known prior diagnosis of myalgic encephalomyelitis/chronic fatigue syndrome (ME/CFS), preceding and not related to SARS-CoV-2 infection and not worsened since SARS-CoV-2 infection.
18. Known prior diagnosis of dysautonomia, preceding and not related to SARS-CoV-2 infection and not worsened since SARS-CoV-2 infection.
19. History of anaphylaxis or hypersensitivity upon receiving IV antibody infusions in the past.
20. Known allergy to any components used in the formulation of the intervention.
21. History of anaphylaxis or similar significant allergic reaction to prescription or non-prescription drugs or food products. Similarly, presence of severe atopic conditions as assessed by the PI represents significant risk for allergic reaction.
22. Pregnant, breastfeeding, or unwilling to practice birth control abide by the contraception requirements outlined in the inclusion criteria.
23. Participation in a clinical trial with receipt of an investigational product within 28 days or 5 half-lives (whichever is longer) prior to planned Day 0. For PET tracers specifically, which have a short half-life and are not biologically active outside this window, elapse of 5 half-lives prior to the planned D0 infusion is sufficient.
24. Current alcohol or illicit drug use as determined by the investigator to preclude participation.
25. Presence of a condition or abnormality that in the opinion of the Investigator would compromise the safety of the patient or the quality of the data.
26. Study site personnel directly affiliated with the study or family of directly involved personnel.
27. History of cytokine release syndrome (CRS) secondary to infection and/or medication.

7 CONCURRENT MEDICATIONS

All subjects should be maintained on the same medications throughout the entire study period, as medically feasible, with no introduction of new chronic therapies unless deemed necessary by a medical provider. Initiation of over-the-counter supplements will be discouraged.

7.1 Allowed Medications and Treatments

Participation in the study will not interfere in any manner with the participant's standard of care. Given the potential immune-modifying activities of AER002, other immunomodulatory drugs (e.g., cytokines, systemic corticosteroids, biologics) will be discouraged if medically feasible. Routine or standard of care vaccinations (such as influenza, pneumococcus, etc.) are allowed and will be documented in the CRFs; wherever possible these will be spaced greater than or equal to two weeks from study visits.

8 STUDY TREATMENTS

8.1 Method of Assigning Subjects to Treatment Groups

The 30 participants will be randomized, 20 to AER002 and 10 to placebo. The randomization will be stratified by duration of COVID-19 related symptoms (< 6 months vs. ≥ 6 months). Within the strata, randomization will use permuted blocks of 3 and 6 enrollees with equal likelihood.

8.2 Blinding

Treatment assignments will be blinded to the investigator, subjects, and all clinical and research staff for the entire study, except for designated pharmacy staff or delegate who will remain unblinded to prepare the study drug or placebo.

Unblinding can occur at any time for a medical emergency or any other significant medical event and when a treatment decision is contingent on knowing the subject's treatment assignment. When possible, the investigator or delegate should discuss with Aerium Therapeutics prior to unblinding. The date and reason for unblinding must be recorded and Aerium Therapeutics will be notified.

The Safety Monitoring Committee (SMC) may review any unblinded clinical analysis during the study and provide the recommendations to the Investigator. Subject level unblinded data will not be shared with the Investigator until the end of the trial.

8.3 Formulation of Test and Control Products

[redacted]

[redacted]

[redacted]

[redacted]

8.3.1 Packaging and Labeling

[redacted]

8.4 Supply of Study Drug at the Site

The manufacturer (or designee) will ship AER002 and placebo to the investigational site's pharmacy. The initial AER002 and placebo shipment will be shipped after site activation (i.e., all required regulatory documentation has been received by Aerium Therapeutics and a contract has been executed). Subsequent AER002 and/or placebo shipments will be made after site request for resupply.

8.4.1 Dosage/Dosage Regimen

AER002 is administered as a one-time 1200 mg IV dose. Placebo is also administered via IV. Details regarding the preparation and administration of the study drugs are provided in the pharmacy manual.

There is no adjustment for weight, age, meals, or other factors.

8.4.2 Dispensing

To ensure treatment compliance, all doses will be administered under the supervision of an investigator (or delegate).

8.4.3 Administration Instructions

[redacted]

[redacted]

[redacted]

[redacted]

8.5 Supply of Study Drug at the Site

The investigator will delegate responsibility for drug receipt, storage, accountability, and disposition to the clinical site's research pharmacist. The clinical site's pharmacist will maintain an inventory record of the study drugs received, stored (in a secure restricted area), and dispensed. Study drugs will be provided to study participants only.

8.5.1 Storage

[redacted]

8.6 Study Drug Accountability

An accurate and current accounting of the dispensing for each subject will be maintained on an ongoing basis by the research pharmacist. The number of study drug dispensed will be recorded on the Investigational Drug Accountability Record. The study monitor will verify these documents throughout the course of the study.

8.7 Measures of Treatment Compliance

Since this study involves a single intravenous intervention, additional measures of treatment compliance will not be needed.

9 STUDY PROCEDURES AND GUIDELINES

A Schedule of Events representing the required testing procedures to be performed for the duration of the study is diagrammed in Appendix 1. The Schedule of Events will serve as the final resource for guiding study activities.

Prior to conducting any study-related activities, written informed consent and the Health Insurance Portability and Accountability Act (HIPAA) authorization must be signed and dated by the subject or subject's legal representative. If appropriate, assent must also be obtained prior to conducting any study-related activities.

9.1 Clinical Assessments

9.1.1 Concomitant Medications

All concomitant medication and concurrent therapies taken within 28 days prior to Screening will be documented. Dose, route, unit frequency of administration, and indication for administration and actual or estimated dates of medication will be captured.

Details regarding all SARS-CoV-2 vaccinations and/or boosters, as well as COVID-specific therapies, will be collected.

9.1.2 Demographics

Demographic information (date of birth, gender, race) will be recorded at Screening.

9.1.3 Medical History

Relevant medical history, including history of current disease, other pertinent respiratory history, and information regarding underlying diseases will be recorded at Screening. This may include assessments for conditions thought to intersect with Long COVID including myalgic encephalomyelitis/chronic fatigue syndrome (ME/CFS), postural orthostatic tachycardia syndrome (POTS), hypermobility syndromes, endometriosis, and mast cell activation syndrome.

9.1.4 Physical Examination

A complete physical examination at Screening will be performed by a qualified study clinician (physician, nurse, or physician assistant). Qualified staff (MD, DO, NP, RN, or PA) may also complete the abbreviated (targeted) physical exam at all other visits. New abnormal physical exam findings must be documented and will be followed by a physician or other qualified staff at the next scheduled visit.

9.1.5 Vital Signs and Oximetry

Weight, body temperature, blood pressure, pulse, respirations, and oximetry on room air will be performed after resting for 5 minutes or more as indicated in the Schedule of Events (SOE). Vital signs in the lying, seated, and standing positions may also be performed.

9.1.6 Questionnaire-based Measurements

Medical History Questionnaires. As part of the medical history at the Screening Visit, participants may be assessed for several medical conditions thought to be related to Long COVID. These may include questions or checklists about chronic fatigue symptoms, hypermobility symptoms, endometriosis symptoms (ENDOPAIN-4D scale) and mast cell activation syndrome (Mast Cell Activation Questionnaire).

Long COVID Symptom Assessment. Signs and symptoms related to Long COVID will be reviewed at all visits, using the LIINC CRFs. This will include the presence and absence of Long COVID symptoms within the prior 30 days and the prior 2 days, as well as information about how bothersome the symptoms are. Long COVID symptoms are one of the primary outcomes of the study.

Other Symptom Assessment (AE Review). In addition to Long COVID symptoms, at D0 and all subsequent visits, all grades of signs and symptoms that are newly developed or changed since the previous visit will be recorded. Symptoms not previously experienced as part of Long COVID, or which have worsened beyond the degree previously experienced by the participant, will be considered to represent adverse events and recorded as such. For these symptoms, duration (start and stop dates), severity/grade, outcome, treatment, and relation to study drug will be recorded on the case report forms. All clinical events and new diagnoses or changes in diagnoses should be recorded.

Quality of Life. Quality of life (QoL) will be obtained using a scale such as the EQ-5D-5L scale which is part of the LIINC CRFs. This will include the 100-point visual analogue scale (VAS).

PROMIS-29. The PROMIS-29 form will be completed as outlined in the schedule of events. This scale measures self-reported health, using a collection of short forms assessing fatigue, physical function, anxiety, depression, pain, sleep disturbance, and ability to participate in social roles and activities. The questionnaire uses a computer interface over approximately 5 minutes. It is available in English and Spanish. This instrument has been used for ME/CFS and Long COVID.

Patient Global Impression of Change (PGIC). The self-report measure Patient Global Impression of Change (PGIC) reflects a patient's belief about the efficacy of treatment. We will use a modified PGIC scale which has been used to study pain syndromes and has been employed in other Long COVID clinical trials. It is a common data element developed by the National Institutes of Mental Health.

Neurocognitive Assessment. A neurocognitive assessment such as the NIH Toolbox or similar assessment will be administered as outlined in the schedule of events. Neurocognitive assessments can assess motor, emotional, sensory, and cognitive function. It is available in English and Spanish and is administered using an iPad or computer.

Duke Activity Status Index. The Duke Activity Status Index is a patient-reported estimate of functional capacity, maximal oxygen consumption (VO2 max) and maximum metabolic equivalent of tasks (METs). The DASI questionnaire produces a score between 0 and 58.2 points, which is linearly correlated with a patient's VO2 max and METs, as measured from cardiopulmonary exercise testing (CPET). It inquires about a person's ability to perform self-care, walk, climb stairs, run, do house and yard work, engage in sexual intercourse, and perform moderate recreational activities.

Post-COVID Functional Status Scale. This is an ordinal tool used to measure the full spectrum of functional outcomes and tracking functional status over time. It assesses a person's ability to live alone, conduct household activities, determines the presence of pain and mental health symptoms, and the impact of these symptoms on regular activities.

Dysautonomia Assessment (e.g., COMPASS-31 or similar Scale). The Composite Autonomic Symptom Score (COMPASS)-31 is an assessment of autonomic dysfunction. This abbreviated questionnaire provides a quantitative measure of autonomic symptoms that otherwise might not be adequately recorded using the above instruments. It includes measures of orthostatic intolerance, vasomotor and secretomotor symptoms, GI and urinary symptoms, syncope, and genitourinary symptoms. We will utilize this questionnaire or a similar instrument to determine the impact of treatment on post-COVID autonomic dysfunction.

DSQ-PEM (Short Form). The DePaul Symptom Questionnaire (DSQ) post-exertional malaise (PEM) questionnaire will be used to assess post-exertional malaise at baseline, and again at the primary endpoint as specified in the schedule of events.

WHO-DAS (Short Form). The World Health Organization Disability Assessment Schedule 2.0 questionnaire asks about difficulties due to health conditions. Health conditions include diseases or illnesses, other health problems that may be short or long lasting, injuries, mental or emotional problems, and problems with alcohol or drugs. We will utilize this questionnaire at baseline, and again at the primary endpoint as specified in the schedule of events.

6 Minute Walk Test. A 6MWT will be performed by the research team.

9.1.7 Adverse Events

Information regarding occurrence of adverse events will be captured throughout the study. Duration (start and stop dates), severity/grade outcome, treatment and relation to study drug will be recorded on the case report form (CRF).

This is a study of Long COVID symptoms. As a result, symptoms experienced prior to receipt of the study intervention will be documented, and a symptom will only be considered to represent an AE if its quality or severity differs from or exceeds that which was previously experienced by the participant.

9.1.8 Infusion-related Site Reactions (IRSRs)

The following system will be used to grade IRSRs:

Infusion reaction to IV IP	None (0)	Mild (Grade 1)	Moderate (Grade 2)	Severe (Grade 3)	Potentially Life Threatening (Grade 4)
Pain (Note: Pain is perceived by the subject without touching.)	No pain	Does not interfere with activity	Repeated use of non-narcotic pain reliever > 24 hours OR interferes with activity	Any use of narcotic pain reliever OR prevents daily activity	Emergency room (ER) visit or hospitalization
Tenderness (Note: Tenderness is pain or discomfort when an affected body part is palpated or moved. It is possible to have both pain & tenderness.	No tenderness	Mild discomfort to touch	Discomfort with movement	Significant discomfort (guarding) at rest<br><br>Note: Subject may avoid moving OR limits movement of affected area	ER visit or hospitalization
Erythema (Redness)	No erythema	2.5 – 5 cm	5.1 – 10 cm	> 10 cm	Necrosis or exfoliative dermatitis
Induration*	No induration	2.5 – 5 cm and does not interfere with activity	5.1 – 10 cm or interferes with activity	> 10 cm or prevents daily activity	Necrosis

Edema**	No edema	2.5 – 5 cm and does not interfere with activity	5.1 – 10 cm or interferes with activity	> 10 cm or prevents daily activity	Necrosis
Pruritus (itch)	No pruritus	Mild pruritus. Does not interfere with activity	Repeated use of anti-pruritic medication > 24 hours OR interferes with activity	Prevents daily activity	ER visit or hospitalization
Accompanying sensory symptoms: Burning, tingling, and/or stinging	None	Mild sensory symptoms. Does not interfere with activity	Interferes with activity	Prevents daily activity	ER visit or hospitalization

*Skin induration is a deep thickening of the skin that can result from edema, infiltration, or inflammation. Diagnosis of skin induration is made by palpation and assessing whether the affected area has a hard resistant feeling.

**Edema is an abnormal buildup of fluid in the body; swelling

Reference: Table modified from Guidance for Industry. Toxicity Grading Scale for Healthy Adult and Adolescent Volunteers Enrolled in Preventive Vaccine Clinical Trials. September 2007. US Department of Health and Human Services (HHS), Food and Drug Administration (FDA), Center for Biologics Evaluation and Research (CBER).

9.1.9 Participants with Positive COVID Tests During the Study

Due to the ongoing pandemic, it is anticipated that some individuals may test positive for COVID-19 during the course of the study.

If an individual tests positive for SARS-CoV-2 infection on a clinical swab during the screening procedures, they will not be eligible to participate unless viral S gene sequencing is performed and confirmed to demonstrate a variant susceptible to AER002.

If an individual exhibits acute COVID-19 symptoms during the study, we will arrange for or navigate to testing. If an individual tests positive for SARS-CoV-2 during the course of the study, we will leverage existing testing and treatment programs in our hospital system and/or geographic region to navigate the participant to care. In addition to navigation for clinical care, all participants who test positive during the study will be referred to a home-based research collection protocol for more frequent sampling (see Appendix 3).

9.2 Clinical Laboratory Measurements

9.2.1 Hematology

Blood will be obtained and sent to each site's clinical hematology lab for a complete blood count (hemoglobin, hematocrit, red blood cell count, white blood cell count, white blood cell differential, and platelet count), erythrocyte sedimentation rate (ESR), and serum C-reactive protein (CRP) determinations for assessment of systemic evidence for infection and/or inflammation. These measurements will be performed as outlined in the Schedule of Events.

9.2.2 Blood Chemistry Profile

Blood will be obtained and sent to each site's clinical chemistry lab for determination of serum sodium, potassium, chloride, bicarbonate, random glucose, BUN, creatinine, aspartate aminotransferase (AST/SGOT), alanine aminotransferase (ALT/SGPT), alkaline phosphatase, total bilirubin, direct bilirubin, and albumin. These measurements will be performed as outlined in the Schedule of Events.

9.2.3 Coagulation Studies

Coagulation studies will be performed at Screening, unless a PT-INR and PTT are available within the prior 6 months. Additional coagulation studies, including D-Dimer and Fibrinogen levels, will be performed as part of the study outcome measurements.

9.2.4 Pregnancy Testing

A urine or serum pregnancy test will be obtained from female subjects who are of childbearing age prior to their participation in the study. These measurements will be performed as outlined in the Schedule of Events.

9.2.5 Hepatitis B Testing

Participants will undergo Hep B surface antigen testing at Screening, if they have not had such screening within the preceding 6 months. Individuals with active Hep B will be excluded. This is defined as detectable Hep B surface antigen. Individuals without detectable Hep B surface antigen will remain eligible, regardless of their vaccination or antibody status (e.g., a positive Hep B cAb or negative Hep B sAb is not exclusionary).

9.2.6 Hepatitis C Testing

Participants will undergo Hep C antibody testing at Screening, if they have not had such screening within the preceding 6 months. Individuals with active Hep C, defined as positive or indeterminate antibody with the presence of Hep C RNA, will be excluded. Positive or indeterminate Hep C Ab in the absence of Hep C RNA is not exclusionary.

9.2.7 HIV Testing

HIV antibody testing will not be required prior to participation in the study but can be performed at the discretion of the PI if requested by a study volunteer. A history of HIV infection on its own is not exclusionary. Participants with controlled HIV infection will remain eligible for the study. If a potential participant is HIV positive, up to 6 months of medical records preceding the Screening visit will be reviewed to evaluate for virus control. Individuals with HIV who are viremic and not on ART will be excluded. Furthermore, those with 2 or more consecutive, quantifiable plasma HIV RNA levels >48 copies/mL in the preceding 6 months, even if on ART, will be excluded. Finally, a plasma HIV RNA test will be performed as part of the Screening procedures for any participant known or found to be HIV positive, if such a test is not available in the 60 days preceding the Screening date.

9.2.8 SARS-CoV-2 PCR or Antigen Testing

Swabs of the anterior nares and/or nasopharynx will be performed to assess for SARS-CoV-2 shedding as outlined in the Schedule of Events.

9.3 Research Laboratory Measurements

9.3.1 Plasma Measures of Viral Persistence

Plasma levels of SARS-CoV-2 antigens (Spike, nucleocapsid, etc.) may be performed in collaboration with research laboratories to evaluate the effect of mAb therapy on measures of viral persistence.

9.3.2 Immunophenotyping

Single- or multi-plex platforms will be used to measure targeted biomarkers previously shown to be elevated in Long COVID, which may include interleukin-6, tumor necrosis factor-alpha, monocyte chemoattractant protein-1, and interferon-gamma induced protein 10. In addition, we will use high-throughput platforms such as Olink proteomics to conduct within-person and/or between-group comparisons in order to identify pathways that are altered with treatment, or that differ between those with and without improvement during the study period. Additional measures of cellular and humoral immunity may also be performed.

9.3.3 Pharmacokinetic (PK) analyses)

Blood samples for determination of serum concentrations of AER002 will be collected at following timepoints:

- Pre-dose
- Post dose:
  - 1 hour (acceptable +/- 10 min)
  - D15 (acceptable +/- 3 Days)
  - D30 (acceptable +/- 5 Days)
  - D90 (acceptable +/- 14 Days)
  - D180 (acceptable +/- 14 Days)
  - D360 (acceptable +/- 28 Days)

9.3.4 Immunogenicity analyses (ADA)

Blood samples for determination of Anti-drug antibodies (ADA)s of AER002 will be collected at following timepoints:

- Pre-dose
- Post dose:
  - 1 hour (acceptable +/- 10 min)
  - D15 (acceptable +/- 3 Days)
  - D30 (acceptable +/- 5 Days)
  - D90 (acceptable +/- 14 Days)
  - D180 (acceptable +/- 14 Days)
  - D360 (acceptable +/- 28 Days)

If no ADA are detected through Day 90, the D180 and D360 analyses will not be performed.

9.3.5 SARS-CoV-2 Genotyping

Genotyping may be performed on any samples collected during the study in which SARS-CoV-2 virus is recovered.

9.4 Biospecimens

Within the UCSF LIINC infrastructure, initial sample processing is performed on peripheral blood units which are then transported by a staff member or courier to a biorepository, which include the Molecular and Cellular Core (formerly Core Immunology Laboratory) and the UCSF Specimen Processing and Banking Subcore (formerly AIDS Specimen Bank). All samples are tracked through laboratory information systems and managed with sample storage inventories, monthly reports, and institutionally managed data storage and back-up systems. All biospecimens will eventually be stored long-term via the UCSF Specimen Processing and Banking Subcore (formerly AIDS Specimen Bank), where the existing 50,000-specimen LIINC repository is currently housed and managed. Specimens are stored in ultra-low temperature freezers (including liquid nitrogen) with back-up power systems, in which temperature is monitored by a programmable scanning alarm system wired to the university's telephone system. After completion of the primary analyses, samples may be stored indefinitely via the LIINC biorepository for use on other COVID-related pathogenesis studies (as addressed in the consent form), at the discretion of the PI.

10 EVALUATIONS BY VISIT

The details of the visit schedule are outlined below. The Schedule of Events will be the final guide for what events are to occur at each specific study visit.

10.1 Screening Visit (D-28)

Following the informed consent discussion and signed informed consent form, the Screening Assessment will be performed. The inclusion and exclusion criteria will be reviewed with the participant. Screening labs, medical history (including assessment for conditions that intersect with Long COVID), and the details of concomitant medications will be obtained. Details of the participant's COVID-19 history will be reviewed and/or confirmed.

The Screening Assessment will include a physical assessment by a study clinician (physician, nurse, or physician assistant). A symptom assessment, PROMIS-29, and Duke Activity Status Index will be performed as part of the Screening procedures.

Laboratory tests as outlined in the Schedule of Events will be performed. Laboratory tests or other assessments performed for a clinical indication (not exclusively to determine study eligibility) may be used for screening values even if the studies were performed before informed consent was obtained. An individual who recently had laboratory tests performed as part of routine clinical care need not repeat these tests if they are within the window. For example, someone with recent Hepatitis B or C or HIV testing may not need to have the test repeated, as indicated in the Schedule of Events. However, these measurements may be repeated at the discretion of the PI.

Screening evaluations should be completed within 28 days of D0. If more than 28 days lapse, screening procedures may be repeated to re-confirm eligibility prior to D0. At a minimum, this will include the metabolic panel, complete blood count, and pregnancy testing. Laboratory tests that are not expected to change (such as coagulation studies or viral hepatitis status) are not required to be repeated but may be at the discretion of the PI.

If needed to reduce participant burden (at the request of the participant or investigator team) the assessments from the Screening Visit may be conducted over two visits during the screening period, but all Screening assessments must be completed to confirm eligibility prior to the conduct of the procedures from the Baseline visit.

Rescreening: A participant who does not meet all eligibility criteria at the time of Screening may be given the opportunity to rescreen for the study at a later date.

10.2 Baseline Evaluation (D-7)

An additional visit will be performed following Screening, at least 7 days after the screening visit and prior to D0. The target date for this visit is within 7 days prior to the scheduled date of study product administration, although up to 21 days is acceptable. The purpose of this visit is to make additional assessments prior to the intervention, to establish baseline symptomatology and measurements. This will include a symptom assessment and various clinical assessments as outlined in the Schedule of Events. Certain real-time laboratory tests will be obtained to establish a baseline.

If needed to reduce participant burden (at the request of the participant or investigator team) the assessments from the Baseline Visit may be conducted over two visits during the baseline period, but all Baseline assessments must be completed prior to the day of the infusion visit.

10.3 Intervention (D0)

Participants who screen into the study and complete baseline measurements will be randomized to receive either AER002 or placebo. They will complete a symptom assessment. During the D0 visit prior to infusion or at the reminder call prior to that visit, a team member will confirm that there have been no major changes to the participant's health since the baseline visit that would affect eligibility. If there have been major changes, this will be discussed with a study clinician prior to infusion to confirm whether or not it is acceptable to proceed.

Vital signs will be performed as follows:

(1) Within approximately 1 hour prior to administration of the study product.
(2) Within approximately 20 minutes of completion of the administration of the study product.
(3) Prior to departure from the unit at which the infusion is administered.
(4) As needed at any other point during the visit, at the discretion of the staff or the PI or in the case of concerning signs or symptoms experienced by the participant.

The PI and/or study nurse should be notified for further guidance if any of the following vital sign abnormalities are confirmed on repeat measurement:

(1) Temperature >38 degrees Celsius.
(2) Resting heart rate >120 or <40 beats per minute.
(3) Resting systolic blood pressure >180 or <85 mmHg.
(4) Resting respiratory rate >30 breaths per minute.
(5) Resting oxygen saturation <90%.

In such a case, the PI will provide guidance on how to proceed. In general, the above abnormalities must resolve prior to administration of the study product and prior to participant discharge from the unit.

Participants will be observed for at least 120 minutes following the product administration and provided with return precautions should they develop any concerning signs or symptoms over the subsequent 24 hours.

10.4 Telephone Follow-up (Phone Follow-Up, D1)

The participant will be contacted by telephone to check on the overall response to the infusion. This will include the DSQ-PEM instrument administered over the phone to assess post-exertional symptoms.

10.5 Follow-up Visits (D15, D30, D90, D180, D360)

In-person follow-up visits will occur as outlined in the Schedule of Events. At each visit, vital signs will be collected, and the participant will undergo a targeted physical assessment as needed. They will complete clinical assessments as outlined in the Schedule of Events. Laboratory tests will be ordered and biospecimens collected and stored for later testing as per the Schedule of Events.

10.6 Unscheduled (Interim) Visits

An unscheduled (or interim) visit is defined as any visit not pre-specified in the study protocol. An unscheduled visit may be scheduled in order to assess a participant in person, repeat a laboratory test, or to collect additional biospecimens at the discretion of the PI.

10.7 Study Discontinuation Visit

If a subject withdraws or discontinues from the study, they should complete a Study Discontinuation Visit. If D0 has occurred, this visit should at a minimum include AE assessment and documentation of the reason for discontinuation or withdrawal. Whenever possible, the study events for the Study Discontinuation Visit should be as follows:

- Prior to D0: No further assessment needed.
- Between D0 and D15: Complete D15 assessments.
- After D15: Complete D90 assessments (even if D90 has elapsed).

11 ADVERSE EXPERIENCE REPORTING AND DOCUMENTATION

11.1 Adverse Events

An adverse event (AE) is any untoward medical occurrence in a clinical investigation of a patient administered a pharmaceutical product, which does not necessarily have a causal relationship with the treatment. An AE is therefore any unfavorable and unintended sign (including an abnormal laboratory finding), symptom or disease temporally associated with the administration of an investigational product, whether or not related to that investigational product. An unexpected AE is one of a type not identified in nature, severity, or frequency in the current Investigator’s Brochure or of greater severity or frequency than expected based on the information in the Investigator’s Brochure.

Suspected Adverse Reaction (SAR) - any AE for which there is a reasonable possibility that the drug caused it. It implies a lesser degree of certainty about causality than **adverse reaction** (any AE caused by a drug).

An unexpected AE or unexpected SAR is one of a type not identified in nature, severity, or frequency in the current Investigator's Brochure or of greater severity or frequency than expected based on the information in the Investigator's Brochure.

The Investigator will probe, via discussion with the subject, for the occurrence of AEs during each subject visit and record the information in the site's source documents. Adverse events will be recorded in the patient CRF. Adverse events will be described by duration (start and stop dates and times), severity, outcome, treatment and relation to study drug, or if unrelated, the cause.

11.1.1 AE Severity

Except for infusion-related site reactions, the National Institutes of Health Division of AIDS (DAIDS) Table for Grading the Severity of Adult and Pediatric Adverse Events, Corrected Version 2.1 (July 2017) should be used to assess and grade AE severity, including laboratory abnormalities judged to be clinically significant. The modified criteria can be found in the study manual. If the experience is not covered in the modified criteria, the guidelines shown in Table 2 below should be used to grade severity. It should be pointed out that the term "severe" is a measure of intensity and that a severe AE is not necessarily serious. Infusion-related site reactions will be graded according to protocol section 9.1.8.

Table 2. AE Severity Grading

Severity (Toxicity Grade)	Description
Mild (1)	Transient or mild discomfort; no limitation in activity; no medical intervention or therapy required. The subject may be aware of the sign or symptom but tolerates it reasonably well.
Moderate (2)	Mild to moderate limitation in activity, no or minimal medical intervention/therapy required.
Severe (3)	Marked limitation in activity, medical intervention/therapy required, hospitalizations possible.
Life-threatening (4)	The subject is at risk of death due to the adverse experience as it occurred. This does not refer to an experience that hypothetically might have caused death if it were more severe.

11.1.2 AE Relationship to Study Drug

The relationship of an AE to the study drug should be assessed using the following the guidelines in Table 3 below.

Table 3. AE Relationship to Study Drug

Relationship to Drug	Comment
Definitely	Previously known toxicity of agent; or an event that follows a reasonable temporal sequence from administration of the drug; that follows a known or expected response pattern to the suspected drug; that is confirmed by stopping or reducing the dosage of the drug; and that is not explained by any other reasonable hypothesis.

Probably	An event that follows a reasonable temporal sequence from administration of the drug; that follows a known or expected response pattern to the suspected drug; that is confirmed by stopping or reducing the dosage of the drug; and that is unlikely to be explained by the known characteristics of the subject's clinical state or by other interventions.
Possibly	An event that follows a reasonable temporal sequence from administration of the drug; that follows a known or expected response pattern to that suspected drug; but that could readily have been produced by a number of other factors.
Unrelated	An event that can be determined with certainty to have no relationship to the study drug.

11.2 Serious Adverse Experiences (SAE) or Serious SAR

An SAE is defined as any AE occurring at any dose that results in any of the following outcomes:

- death
- a life-threatening adverse experience
- inpatient hospitalization or prolongation of existing hospitalization
- a persistent or significant disability/incapacity
- a congenital anomaly/birth defect

Other important medical events may also be considered an SAE when, based on appropriate medical judgment, they jeopardize the subject or require intervention to prevent one of the outcomes listed.

A **life-threatening AE or life-threatening SAR** is an occurrence that places the patient or subject at immediate risk of death. It does NOT include an AE or SAR that, had it occurred in a more severe form, might have caused death.

SUSARS are fatal or life threatening serious unexpected SARs. The team must notify the FDA as soon as possible, but no later than 7 calendar days after the initial receipt of information.

11.2.1 Serious Adverse Experience Reporting

Study sites will document all SAEs that occur (whether or not related to study drug) per UCSF CHR Guidelines. The collection period for all SAEs will begin after informed consent is obtained and end after procedures for the final study visit have been completed.

In accordance with the standard operating procedures and policies of the local Institutional Review Board (IRB)/Independent Ethics Committee (IEC), the site investigator will report SAEs to the IRB/IEC.

Aerium Therapeutics, the manufacturer of the IP, shall be notified within 24 hours of PI awareness of an SAE. In addition, an AE master list shall be sent to Aerium Therapeutics monthly.

FDA IND Expedited Safety reporting Requirements

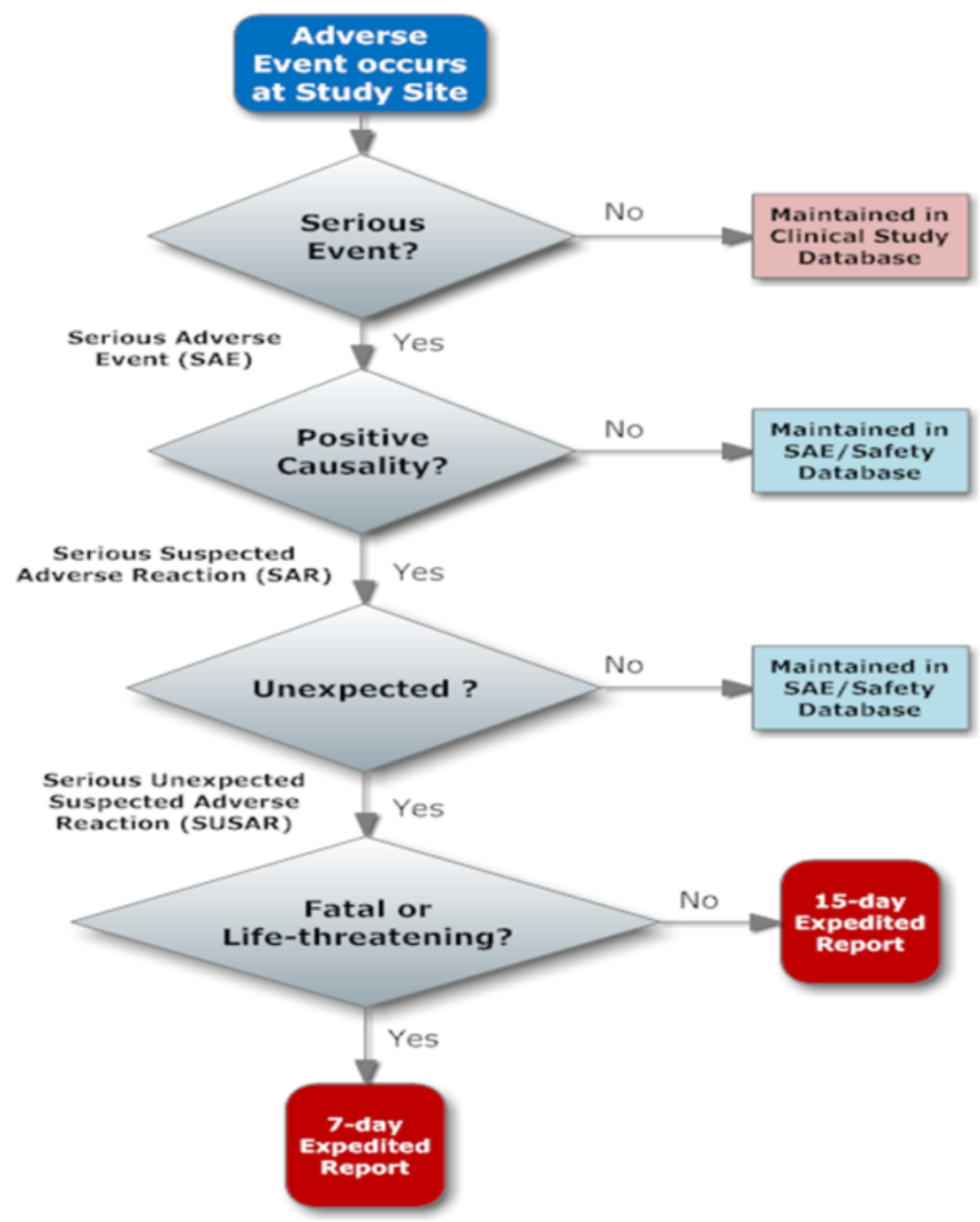

Additional FDA IND 15-day expedited reporting includes new safety findings from other clinical trials, findings from in vitro and animal testing, and increased rate of occurrence of serious SARS.

11.3 Safety Monitoring Committee Chair / Medical Monitoring

Dr. Vincent (Vince) Marconi should be contacted directly at this number to report medical concerns or questions regarding safety.

Phone: 617-501-8462

Email: vcmarco@emory.edu

12 DISCONTINUATION AND REPLACEMENT OF SUBJECTS

12.1 Early Discontinuation

A subject may be discontinued from study treatment at any time if the subject or the investigator feels that it is not in the subject's best interest to continue. The following is a list of possible reasons for study treatment discontinuation:

- Subject withdrawal of consent
- Subject is not compliant with study procedures
- Adverse event that in the opinion of the investigator would be in the best interest of the subject to discontinue study treatment
- Protocol violation requiring discontinuation of study treatment
- Lost to follow-up
- Manufacturer request for early termination of study

If a subject is withdrawn from treatment due to an adverse event, the subject will be followed and treated by the Investigator until the abnormal parameter or symptom has resolved or stabilized.

All subjects who discontinue study treatment should come in for an early discontinuation visit (as outlined above) as soon as possible and then should be encouraged to complete all remaining scheduled visits and procedures.

All subjects are free to withdraw from participation at any time, for any reason, specified or unspecified, and without prejudice.

Reasonable attempts will be made by the investigator to provide a reason for subject withdrawals. The reason for the subject's withdrawal from the study will be specified in the subject's source documents Refer to Section 10 for study discontinuation procedures.

12.2 Withdrawal of Subjects from the Study

A subject may be withdrawn from the study at any time if the subject or the investigator feels that it is not in the subject's best interest to continue.

All subjects are free to withdraw from participation at any time, for any reason, specified or unspecified, and without prejudice.

Reasonable attempts will be made by the investigator to provide a reason for subject withdrawals. The reason for the subject's withdrawal from the study will be specified in the subject's source documents. As noted above, subjects who discontinue study treatment early (i.e., they withdraw prior to D360) should have an early discontinuation visit. Refer to Section 10 for early termination procedures.

12.3 Replacement of Subjects

Subjects who withdraw from the study prior will be replaced at the discretion of the investigator.

12.4 Lost to Follow Up

A subject will be considered LTFU if s/he repeatedly fails to return for scheduled visits and is unable to be contacted by the study site. The following actions must be taken if a subject fails to return to the clinic for a required study visit:

- The site must attempt to contact the subject and reschedule the missed visit as soon as possible, counsel the subject on the importance of maintaining the assigned visit schedule and ascertain whether the subject wishes to and/or should continue in the study.
- Before a subject is deemed LTFU, the investigator or designee must make every effort to regain contact with the subject (at a minimum, 2 telephone calls and, if necessary, a certified letter to the subject's last known mailing address or local equivalent methods). These contact attempts should be documented in the subject's source documents.

Should the subject continue to be unreachable, s/he will be considered to have withdrawn from the study.

13 PROTOCOL VIOLATIONS

A protocol violation occurs when the subject or investigator fails to adhere to significant protocol requirements affecting the inclusion, exclusion, subject safety, and primary endpoint criteria. Protocol violations for this study include, but are not limited to, the following:

- Failure to meet inclusion/exclusion criteria
- Use of a prohibited concomitant medication

Failure to comply with Good Clinical Practice (GCP) guidelines will also result in a protocol violation. The Investigator will determine if a protocol violation results in withdrawal of a subject.

When a protocol violation occurs, it will be discussed with the investigator and a Protocol Violation Form detailing the violation will be generated. This form will be signed by the Investigator or delegate. A copy of the form will be filed in the site's regulatory binder and in the Investigator's files.

14 DATA SAFETY MONITORING

The Investigator will establish a Safety Monitoring Committee (SMC) to review data relating to safety, and to ensure the continued scientific validity and merit of the study, according to SMC Charter to be established for this protocol.

Scheduled SMC meetings will occur as outlined in the SMC Charter, approximately 3 times per year (generally about 4 months after the first participant is enrolled, and about every 4 months thereafter). The SMC will meet and review accrual (including screening and enrollment), AE summaries, including all reported Grade ≥3 AEs, retention of participants including off-study rates.

In addition, after 10 individuals have been dosed, the study will pause for further dosing (follow up will still occur) for a blinded SMC review of the 2-week safety data on the first 10 enrollees to ensure that no safety concerns warranting altering or stopping the study are identified; this may take place as part of a regular SMC meeting or an ad hoc meeting depending on timing.

In addition to the regularly scheduled reviews, a safety review will be conducted by the SMC for any of the following criteria:

- Two or more participants experience an SAE that is deemed possibly, probably, or definitely related to the study treatment.
- One or more participants experience a Grade 4 AE that is deemed possibly, probably, or definitely related to the study treatment.

If the above criteria are met, the PI will request a review by the SMC (or the SMC chair if other SMC members cannot be convened), to be held within 3 business days of learning of the event.

Whenever any of the events above occurs, enrollment into the study will be paused until the SMC review has taken place and a determination has been made that enrollment can resume. In addition, administration of study products will be paused until a course of action is recommended by the SMC. Follow-up visits (Baseline Assessments, D1, W2, M1, M3, M6, and M12, and unscheduled visits) can continue during this period of review as these are not expected to affect the safety of participants who have already received the study IP.

The SMC will recommend, based on the results of the review, whether the study can proceed as planned, proceed with modifications, or should be discontinued.

The SMC will review progress towards pre-specified benchmarks of enrollment and retention of participants, completion of study procedures, and collection of viable samples. If progress towards any benchmark is not adequate, as determined by the SMC, the SMC will recommend protocol modification if necessary.

15 STATISTICAL METHODS AND CONSIDERATIONS

Prior to the analysis of the final study data, a detailed Statistical Analysis Plan (SAP) will be written describing all analyses that will be performed. The SAP will contain any modifications to the analysis plan described below.

15.1 Data Sets Analyzed

Eligible patients who are randomized into the study and receive the study drug will comprise the modified intent to treat (mITT) population and will be the primary analysis population for all analyses.

Per protocol analysis will be based on description of the results per study group excluding anyone who has received a non-study COVID-neutralizing mAb during the trial period, anyone with resistant SARS-CoV-2 infections during the trial or receipt of COVID antivirals during the trial.

15.2 Demographic and Baseline Characteristics

The following demographic variables at screening will be summarized by dose level: race, gender, age, height, and weight.

15.3 Analysis of Primary Endpoint

Safety and tolerability data will be summarized by treatment group.

Adverse event rates will be coded by body system. Adverse events will be tabulated by treatment group and will include the number of patients for whom the event occurred, the rate of occurrence, and the severity and relationship to study drug. Adverse events between groups will be summarized by proportions with associated 95% Clopper-Pearson confidence intervals and will be compared using a two-sided 0.05 level Fisher exact test.

15.4 Analysis of Secondary Endpoints

The key secondary outcome will be the changed in the PROMIS-29 score from baseline to D90. This will be analyzed from the mITT population using a linear regression model for follow-up PROMIS-29 with terms for the baseline value, randomized treatment and randomization strata (<6 months v. >= 6 months of symptoms) – an analysis of covariance model (ANCOVA) and tested using a two-sided 0.05 level test.

Continuous secondary events (e.g., IL-6) will be compared using the ANCOVA approach. Antigen detection will be summarized by proportion with 95% Clopper-Pearson confidence intervals and will be compared using a two-sided 0.05 level Fisher exact test.

As above, eligibility is determined by Long COVID attributed to a SARS-CoV-2 infection prior to August 15, 2022. All participants will be required to have Long COVID attributed to an infection prior to this date. A sub-analysis will address individuals with and without a known history of SARS-CoV-2 re-infection after August 15, 2022.

15.5 Interim Analysis

Interim analysis of group level results without subject level unblinding will be conducted for all subjects reaching 3M.

15.6 Sample Size and Randomization

This is an exploratory study, and the primary results will be descriptive. We will use AER002 as a probe to better understand LC biology and determine whether a signal is present to warrant further study.

The study will enroll 30 participants who meet the WHO LC criteria. Participants will be enrolled at a single center and randomized 2:1 to receive AER002 1200mg or placebo. Randomization will be stratified by duration of infection (<6 months and ≥6 months) at Day 0.

For PROMIS-29, assuming a standard deviation (SD) of the within-person difference of 10, then the planned sample size will have 80% power to detect a difference of 11.2 on a two-sided 0.05 level test.

For biomarkers, such as IL-6, we will be able to detect a difference from a mean value of 2.5 pg/mL to 1.1 times the within-person standard deviation with 80% power.

We assume that 25% of participants will have positive antigen at entry, we will have 62% power to detect a reduction to < 1% with positive antigen on the treatment arm.

16 DATA COLLECTION, RETENTION AND MONITORING

16.1 Data Collection Instruments

The Investigator will prepare and maintain adequate and accurate source documents designed to record all observations and other pertinent data for each subject treated with the study drug.

Study personnel at each site will enter data from source documents corresponding to a subject's visit into the protocol-specific electronic Case Report Form (eCRF) or paper CRF when the information corresponding to that visit is available. Subjects will not be identified by name in the study database or on any study documents to be collected by the Investigator (or designee) but will be identified by their unique PID.

For eCRFs: If a correction is required for an eCRF, the time and date stamps track the person entering or updating eCRF data and creates an electronic audit trail. For paper CRFs: If a correction is made on a CRF, the study staff member will line through the incorrect data, write in the correct data and initial and date the change.

The Investigator is responsible for all information collected on subjects enrolled in this study. All data collected during the course of this study must be reviewed and verified for completeness and accuracy by the Investigator.

16.2 Data Management Procedures

The data will be entered into a validated database (REDCap). The Data Management group will be responsible for data processing, in accordance with procedural documentation.

All procedures for the handling and analysis of data will be conducted using good computing practices meeting FDA guidelines for the handling and analysis of data for clinical trials.

16.3 Data Quality Control and Reporting

After data have been entered into the study database, a system of computerized data validation checks will be implemented and applied to the database on a regular basis. Query reports (Data Clarification Requests) pertaining to data omissions and discrepancies will be forwarded to the study team for resolution. The study database will be updated in accordance with the resolved queries.

16.4 Archival of Data

The database is safeguarded against unauthorized access by established security procedures; appropriate backup copies of the database and related software files will be maintained. Databases are backed up by the database administrator in conjunction with any updates or changes to the database.

At critical junctures of the protocol (e.g., production of interim reports and final reports), data is cleaned per established procedures.

16.5 Availability and Retention of Investigational Records

The Investigator must make study data accessible to the monitor, IRB/IEC, and Regulatory Agency (e.g., FDA) inspectors upon request. A file for each subject must be maintained that includes the signed Informed Consent, HIPAA Authorization and Assent Form and copies of all source documentation related to that subject. The Investigator must ensure the reliability and availability of source documents from which the information on the CRF was derived.

All study documents (patient files, signed informed consent forms, copies of CRFs, Study File Notebook, etc.) must be kept secured for a period of two years following marketing of the investigational product or for two years after centers have been notified that the IND has been discontinued. There may be other circumstances for which the Investigator is required to maintain study records and, therefore, the Investigator should be contacted prior to removing study records for any reason.

As outlined above, after completion of the primary analyses, samples may be stored indefinitely via the LIINC biorepository for use on other COVID-related pathogenesis studies (as addressed in the consent form), at the discretion of the PI.

16.6 Monitoring

Monitoring visits will be conducted by representatives of the Investigator according to the U.S. CFR Title 21 Parts 50, 56, and 312 and ICH Guidelines for GCP (E6). By signing this protocol, the Investigator grants permission to the monitor, and appropriate regulatory authorities to conduct on-site monitoring and/or auditing of all appropriate study documentation.

16.7 Subject Confidentiality

In order to maintain subject confidentiality, only a subject PID will identify all study subjects on CRFs and other documentation. Additional subject confidentiality issues (if applicable) are covered in the Clinical Study Agreement.

17 ADMINISTRATIVE, ETHICAL, REGULATORY CONSIDERATIONS

The study will be conducted according to the Declaration of Helsinki, Protection of Human Volunteers (21 CFR 50), Institutional Review Boards (21 CFR 56), and Obligations of Clinical Investigators (21 CFR 312).

To maintain confidentiality, all laboratory specimens, evaluation forms, reports and other records will be identified by a coded number and initials only. All study records will be kept in a locked file cabinet and code sheets linking a patient’s name to a patient identification number will be stored separately in another locked file cabinet. Clinical information will not be released without written permission of the subject, except as necessary for monitoring by the FDA. The Investigator must also comply with all applicable privacy regulations (e.g., Health Insurance Portability and Accountability Act of 1996, EU Data Protection Directive 95/46/EC).

17.1 Protocol Amendments

Any amendment to the protocol will be written by the Investigator or their designee. Protocol amendments cannot be implemented without prior written IRB/IEC approval except as necessary to eliminate immediate safety hazards to patients. A protocol amendment intended to eliminate an apparent immediate hazard to patients may be implemented immediately, provided the IRBs are notified within five working days.

17.2 Institutional Review Boards and Independent Ethics Committees

The protocol and consent form will be reviewed and approved by the IRB/IEC of each participating center prior to study initiation. Serious adverse experiences regardless of causality will be reported to the IRB/IEC in accordance with the standard operating procedures and policies of the IRB/IEC, and the Investigator will keep the IRB/IEC informed as to the progress of the study. The Investigator will obtain assurance of IRB/IEC compliance with regulations.

Any documents that the IRB/IEC may need to fulfill its responsibilities (such as protocol, protocol amendments, Investigator's Brochure, consent forms, information concerning patient recruitment, payment or compensation procedures, or other pertinent information) will be submitted to the IRB/IEC. The IRB/IECs written unconditional approval of the study protocol and the informed consent form will be in the possession of the Investigator before the study is initiated. The IRB/IECs approval statement will be transmitted by the Investigator to Aerium Therapeutics. This approval must refer to the study by exact protocol title and number and should identify the documents reviewed and the date of review.

Protocol and/or informed consent modifications or changes may not be initiated without prior written IRB/IEC approval except when necessary to eliminate immediate hazards to the patients or when the change(s) involves only logistical or administrative aspects of the study. Such modifications will be submitted to the IRB/IEC and written verification that the modification was submitted and subsequently approved should be obtained.

The IRB/IEC must be informed of revisions to other documents originally submitted for review; serious and/or unexpected adverse experiences occurring during the study in accordance with the standard operating procedures and policies of the IRB; new information that may affect adversely the safety of the patients of the conduct of the study; an annual update and/or request for re-approval; and when the study has been completed.

17.3 Informed Consent Form

Informed consent will be obtained in accordance with the Declaration of Helsinki, ICH GCP, US Code of Federal Regulations for Protection of Human Subjects (21 CFR 50.25[a,b], CFR 50.27, and CFR Part 56, Subpart A), the Health Insurance Portability and Accountability Act (HIPAA, if applicable), and local regulations.

The Investigator will prepare the informed consent form and HIPAA authorization for submission to the IRB/IEC. The consent form generated by the Investigator must be approved by the IRB/IEC. The written consent document will embody the elements of informed consent as described in the International Conference on Harmonisation and will also comply with local regulations. The Investigator will store an IRB/IEC-approved copy of the Informed Consent Form for the study file.

A properly executed, written, informed consent will be obtained from each subject prior to entering the subject into the trial. Information should be given in both oral and written form and subjects (or their legal representatives) must be given ample opportunity to inquire about details of the study. If appropriate and required by the local IRB/IEC, assent from the subject will also be obtained. If a subject is unable to sign the informed consent form (ICF) and the HIPAA authorization, a legal representative may sign for the subject. A copy of the signed consent form will be offered to the subject or legal representative of the subject and the original will be maintained with the subject's records.

17.4 Publications

The preparation and submittal for publication of manuscripts containing the study results shall be in accordance with a process determined by mutual written agreement among the study Investigator and participating institutions. The publication or presentation of any study results shall comply with all applicable privacy laws, including, but not limited to, the Health Insurance Portability and Accountability Act of 1996.

17.5 Investigator Responsibilities

By signing the Agreement of Investigator form, the Investigator agrees to:

1. Conduct the study in accordance with the protocol and only make changes after notifying Aerium Therapeutics, except when to protect the safety, rights or welfare of subjects.

2. Personally conduct or supervise the study (or investigation).
3. Ensure that the requirements relating to obtaining informed consent and IRB review and approval meet federal guidelines, as stated in § 21 CFR, parts 50 and 56.
4. Report to Aerium Therapeutics or designee any AEs that occur in the course of the study, in accordance with §21 CFR 312.64.
5. Ensure that all associates, colleagues and employees assisting in the conduct of the study are informed about their obligations in meeting the above commitments.
6. Maintain adequate and accurate records in accordance with §21 CFR 312.62 and to make those records available for inspection with the Aerium Therapeutics (or designee).
7. Ensure that an IRB that complies with the requirements of §21 CFR part 56 will be responsible for initial and continuing review and approval of the clinical study.
8. Promptly report to the IRB and Aerium Therapeutics (or designee) all changes in the research activity and all unanticipated problems involving risks to subjects or others (to include amendments and IND safety reports).
9. Seek IRB approval before any changes are made in the research study, except when necessary to eliminate hazards to the patients/subjects.
10. Comply with all other requirements regarding the obligations of clinical investigators and all other pertinent requirements listed in § 21 CFR part 312.

18 REFERENCES

1. Nearly one in five American adults who have had COVID-19 still have “long COVID” [Internet]. 2022 [cited 2023 Jan 24];Available from: https://www.cdc.gov/nchs/pressroom/nchs_press_releases/2022/20220622.htm

2. Nalbandian A, Sehgal K, Gupta A, et al. Post-acute COVID-19 syndrome. Nat Med [Internet] 2021;Available from: http://dx.doi.org/10.1038/s41591-021-01283-z

3. Prescott HC, Angus DC. Enhancing Recovery From Sepsis: A Review. JAMA 2018;319(1):62–75.

4. Soriano JB, Murthy S, Marshall JC, Relan P, Diaz JV, WHO Clinical Case Definition Working Group on Post-COVID-19 Condition. A clinical case definition of post-COVID-19 condition by a Delphi consensus. Lancet Infect Dis 2022;22(4):e102–7.

5. Davis HE, McCorkell L, Vogel JM, Topol EJ. Long COVID: major findings, mechanisms and recommendations. Nat Rev Microbiol 2023;1–14.

6. Ballotta L, Simonetti O, D’Agaro P, et al. Case report: Long-lasting SARS-CoV-2 infection with post-COVID-19 condition in two patients with chronic lymphocytic leukemia: The emerging therapeutic role of casirivimab/imdevimab. Front Oncol [Internet] 2022;12. Available from: https://www.frontiersin.org/articles/10.3389/fonc.2022.945060

7. Fenwick C, Turelli P, Ni D, et al. SARS-CoV-2 Omicron potently neutralized by a novel antibody with unique Spike binding properties [Internet]. Available from: http://dx.doi.org/10.1101/2022.03.18.484873

8. Gaebler C, Wang Z, Lorenzi JCC, et al. Evolution of antibody immunity to SARS-CoV-2. Nature [Internet] 2021;Available from: http://dx.doi.org/10.1038/s41586-021-03207-w

9. Aydillo T, Gonzalez-Reiche AS, Aslam S, et al. Shedding of Viable SARS-CoV-2 after Immunosuppressive Therapy for Cancer. N Engl J Med 2020;383(26):2586–8.

10. Choi B, Choudhary MC, Regan J, et al. Persistence and Evolution of SARS-CoV-2 in an Immunocompromised Host. N Engl J Med 2020;383(23):2291–3.

11. Zollner A, Koch R, Jukic A, et al. Postacute COVID-19 is Characterized by Gut Viral Antigen Persistence in Inflammatory Bowel Diseases. Gastroenterology 2022;163(2):495–506.e8.

12. Natarajan A, Zlitni S, Brooks EF, et al. Gastrointestinal symptoms and fecal shedding of SARS-CoV-2 RNA suggest prolonged gastrointestinal infection. Med (N Y) [Internet] 2022;Available from: http://dx.doi.org/10.1016/j.medj.2022.04.001

13. Xu Q, Milanez-Almeida P, Martins AJ, et al. Adaptive immune responses to SARS-CoV-2 persist in the pharyngeal lymphoid tissue of children. Nat Immunol 2023;24(1):186–99.

14. Cheung CCL, Goh D, Lim X, et al. Residual SARS-CoV-2 viral antigens detected in GI and hepatic tissues from five recovered patients with COVID-19. Gut. 2022;71(1):226–9.

15. Goh D, Lim JCT, Fernaíndez SB, et al. Case report: Persistence of residual antigen and RNA of the SARS-CoV-2 virus in tissues of two patients with long COVID. Front Immunol 2022;13:939989.

16. Stein SR, Ramelli SC, Grazioli A, et al. SARS-CoV-2 infection and persistence in the human body and brain at autopsy. Nature 2022;612(7941):758–63.

17. Jin JC, Ananthanarayanan A, Brown JA, et al. SARS CoV-2 detected in neonatal stool remote from maternal COVID-19 during pregnancy. Pediatr Res 2022;1–8.

18. Racine T, Kobinger GP. Viral pathogenesis: Unlocking Ebola persistence. Nat Microbiol. 2017;2:17124.

19. Tejerina F, Catalan P, Rodriguez-Grande C, et al. Post-COVID-19 syndrome. SARS-CoV-2 RNA detection in plasma, stool, and urine in patients with persistent symptoms after COVID-19. BMC Infect Dis 2022;22(1):211.

20. Swank Z, Senussi Y, Manickas-Hill Z, et al. Persistent circulating SARS-CoV-2 spike is associated with post-acute COVID-19 sequelae. Clin Infect Dis [Internet] 2022;Available from: http://dx.doi.org/10.1093/cid/ciac722

21. Taylor PC, Adams AC, Hufford MM, de la Torre I, Winthrop K, Gottlieb RL. Neutralizing monoclonal antibodies for treatment of COVID-19. Nat Rev Immunol 2021;21(6):382–93.

22. Peluso MJ, Kelly JD, Lu S, et al. Persistence, magnitude, and patterns of postacute symptoms and quality of life following onset of SARS-CoV-2 infection: cohort description and approaches for measurement. In: Open forum infectious diseases. Oxford University Press US; 2022. p. ofab640.

23. Peluso MJ, Deitchman AN, Torres L, et al. Long-term SARS-CoV-2-specific immune and inflammatory responses in individuals recovering from COVID-19 with and without post-acute symptoms. Cell Rep 2021;109518.

24. Peluso MJ, Lu S, Tang AF, et al. Markers of Immune Activation and Inflammation in Individuals With Postacute Sequelae of Severe Acute Respiratory Syndrome Coronavirus 2 Infection. J Infect Dis [Internet] 2021;Available from: http://dx.doi.org/10.1093/infdis/jiab490

25. Peluso MJ, Sans HM, Forman CA, et al. Plasma Markers of Neurologic Injury and Inflammation in People With Self-Reported Neurologic Postacute Sequelae of SARS-CoV-2 Infection. Neurol Neuroimmunol Neuroinflamm [Internet] 2022;9(5). Available from: http://dx.doi.org/10.1212/NXI.0000000000200003

26. Durstenfeld MS, Peluso MJ, Kelly JD, et al. Role of antibodies, inflammatory markers, and echocardiographic findings in postacute cardiopulmonary symptoms after SARS-CoV-2 infection. JCI Insight [Internet] 2022;7(10). Available from: http://dx.doi.org/10.1172/jci.insight.157053

27. Peluso MJ, Spinelli MA, Deveau T-M, et al. Postacute sequelae and adaptive immune responses in people with HIV recovering from SARS-COV-2 infection. AIDS 2022;36(12):F7–16.

28. Peluso MJ, Thomas IJ, Munter SE, Deeks SG, Henrich TJ. Lack of Antinuclear Antibodies in Convalescent Coronavirus Disease 2019 Patients With Persistent Symptoms. Clin. Infect. Dis. 2022;74(11):2083–4.

29. Peluso MJ, Mitchell A, Wang CY, et al. Low Prevalence of Interferon-α Autoantibodies in People Experiencing Long COVID Symptoms. J Infect Dis [Internet] 2022;Available from: http://dx.doi.org/10.1093/infdis/jiac372

30. Giron LB, Peluso MJ, Ding J, et al. Markers of fungal translocation are elevated during post-acute sequelae of SARS-CoV-2 and induce NF-κB signaling. JCI Insight [Internet] 2022;7(18). Available from: http://dx.doi.org/10.1172/jci.insight.164813

31. Apple AC, Oddi A, Peluso MJ, et al. Risk factors and abnormal cerebrospinal fluid associate with cognitive symptoms after mild COVID-19. Ann Clin Transl Neurol 2022;9(2):221–6.

APPENDIX 1. SCHEDULE OF EVENTS

Target Day	-28	-7	0	1	15	30	90	180	360
**Visit Name**	**Screen**	**Baseline**	**Intervention**	**Phone FU**	**2W**	**1M**	**3M**	**6M**	**12M**
**Visit Window**	**-28 to -8**	**-21 to -1**	**N/A**	**+1**	**+/-3**	**+/-5**	**+/-14**	**+/-14**	**+/-28**
Screening Procedures									
Informed Consent	x								
Eligibility Review	x								
Screening Labs	x								
Demographics	x								
Medical History (inc. ME/CFS, hEDS, EM, MCAS assess.)	x								
COVID-19 History	x								
Concomitant Medications	x	x	x	x	x	x	x	x	x
Study Product Administration									
Randomization			x						
AER002 vs Placebo			x						
Clinical Assessments									
Vital Signs	x	x	x[1]		x	x	x	x	x
Physical Assessment	x	PRN	PRN		PRN	PRN	PRN	PRN	PRN
Long COVID Symptom Assessment (LIINC Quest.)	x	x	x		x	x	x	x	x
Quality of Life, (e.g., EuroQoL)	x	x			x	x	x	x	x
PROMIS-29	x	x			x	x	x	x	x
PGIC Scale							x	x	x
Duke Activity Status Index	x	x			x	x	x	x	x
Post-COVID Functional Scale	x	x			x	x	x	x	x
Dysautonomia Assessment (e.g., COMPASS-31)		x					x	x	x

**Target Day**	**-28**	**-7**	**0**	**1**	**15**	**30**	**90**	**180**	**360**
**Visit Name**	**Screen**	**Baseline**	**Intervention**	**Phone FU**	**2W**	**1M**	**3M**	**6M**	**12M**
**Visit Window**	**-28 to -8**	**-21 to -1**	**N/A**	**+1**	**+/-3**	**+/-5**	**+/-14**	**+/-14**	**+/-28**
DSQ-PEM		x		x	x	x	x	x	X
WHO-DAS		x					X	x	x
Neurocognitive assessment (e.g., NIH Toolbox)		x					x	x	x
6MWT		x			x	x	x	x	x
Safety Outcomes									
AE Review	x	x	x	x	x	x	x	x	x
Safety Labs			x		x				
Real-time laboratory tests									
Pregnancy testing	x		x				x		
Complete metabolic panel	x		x		x				
CBC/diff	x		x		x				
Coagulation studies *Within 6 months of Screen*	x								
Hep B surface antigen *Within 6 months of Screen*	x								
Hep C antibody *Within 6 months of Screen*	x								
Hep C RNA (if Ab positive) *Within 6 months of Screen*	x								
Plasma HIV RNA (if HIV+) *Within 2 months of Screen*	x								
C-reactive protein		x	x		x	x	x	x	x
Erythrocyte sedimentation rate		x	x		x	x	x	x	x
D-dimer		x	x		x	x	x	x	x
Fibrinogen		x	x		x	x	x	x	x
SARS-CoV-2 PCR or Ag test	x	PRN	PRN		PRN	PRN	PRN	PRN	PRN
Stool collection (optional)		x				x		x	x

**Target Day**	**-28**	**-7**	**0**	**1**	**15**	**30**	**90**	**180**	**360**
**Visit Name**	**Screen**	**Baseline**	**Intervention**	**Phone FU**	**2W**	**1M**	**3M**	**6M**	**12M**
**Visit Window**	**-28 to -8**	**-21 to -1**	**N/A**	**+1**	**+/-3**	**+/-5**	**+/-14**	**+/-14**	**+/-28**
Post-hoc laboratory tests									
SARS-CoV-2 antibody testing		x							
Biospecimen storage	x	x	x		x	x	x	x	x
Markers of viral persistence	x	x	x		x	x	x	x	x
Markers of inflammation		x	x		x	x	x	x	x
PK analyses [4]			x[2]		x	x	x	x	x
ADA Immunogenicity [4]			x		x	x	x	x[3]	x[3]
SARS-CoV-2 PCR/ Genotyping	x		x		x	x	x	x	x

[1] *Performed pre-dose and post-dose*

[2] *Collected pre-dose and 1 hour +/- 10 minutes post dose*

[3] *Continue to collect if ADA is detected at previous visit*

[4] *PK and ADA post dose sample collection windows:*

- *D15 (acceptable +/- 3 Days)*
- *D30 (acceptable +/- 5 Days)*
- *D90 (acceptable +/- 14 Days)*
- *D180 (acceptable +/- 14 Days)*
- *D360 (acceptable +/- 28 Days)*

Abbreviations: myalgic encephalomyelitis/chronic fatigue syndrome, ME/CFS; hypermobile Ehler-Danlos Syndrome, hEDS; endometriosis, EM; mast cell activation syndrome, MCAS; DePaul Symptom Questionnaire-Post-Exertional Malaise, DSQ-PEM; postural orthostatic tachycardia syndrome, POTS; pharmacokinetic, PK; anti-drug antibody, ADA.

APPENDIX 2. NIH COVID-19 TREATMENT GUIDELINES DEFINITION OF MODERATE OR SEVERE IMMUNOCOMPROMISE, MARCH 6, 2023

- Are receiving active treatment for solid tumor and hematologic malignancies.
- Have hematologic malignancies (e.g., chronic lymphocytic lymphoma, non-Hodgkin lymphoma, multiple myeloma, acute leukemia) and are known to have poor responses to COVID-19 vaccines or an increased risk of severe COVID-19, regardless of the treatment status for the hematologic malignancy.
- Received a solid-organ or islet transplant and are receiving immunosuppressive therapy.
- Received chimeric antigen receptor T cell (CAR T-cell) therapy or a hematopoietic cell transplant (HCT) and are within 2 years of transplantation or are receiving immunosuppressive therapy.
- Have a moderate or severe primary immunodeficiency (e.g., severe combined immunodeficiency, DiGeorge syndrome, Wiskott-Aldrich syndrome, common variable immunodeficiency disease).
- Have advanced or untreated HIV infection (defined as people with HIV and CD4 T lymphocyte [CD4] cell counts <200 cells/mm^3, a history of an AIDS-defining illness without immune reconstitution, or clinical manifestations of symptomatic HIV).
- Are receiving active treatment with high-dose corticosteroids (i.e., ≥20 mg prednisone or equivalent per day when administered for ≥2 weeks), alkylating agents, antimetabolites, transplant-related immunosuppressive drugs, cancer chemotherapeutic agents classified as severely immunosuppressive, or immunosuppressive or immunomodulatory biologic agents (e.g., B cell–depleting agents).

APPENDIX 3. HOME-BASED ACUTE COVID-19 RESEARCH PROTOCOL

Participants who test positive during the study will be referred for participation in a home-based acute COVID-19 research protocol. That protocol is outlined below.

Time since onset of symptoms	**DE**	**D3**	**D4**	**D5**	D6	D7	D8	D9	D10	D11	D12	D13	**D14**	D17	D19	D21	**D28**
In-person study visits	x	x	x	x									x				x
Baseline survey	x	x	x	x													
Follow-up survey								x					x				x
Clinical antibody	x	x	x	x													x
Nasal swab	x	x	x	x	x	x	x	x	x	x	x	x	x	x	x	x	x
Rapid Antigen Test	x	x	x	x	x	x	x	x	x	x	x	x	x	x	x	x	x

*DE, day of enrollment.

APPENDIX 4. PROTOCOL AMENDMENTS

Date	Version	Section	Changes
2023-MAR-01	1.0	N/A	N/A
2023-MAR-24	1.1	N/A	Addressed initial IRB stipulations
2023-APR-24	1.2	N/A	Addressed FDA suggestions
2023-JUN-24	1.3	N/A	Addressed FDA suggestions<br>Addressed Patient-Led Research Collaborative suggestions<br>Corrected various typos
2023-JUN-24	1.3	2.3.1	Clarified that participants will be screened for signs and symptoms of anemia and tested as outlined in the Schedule of Events (unchanged)
2023-JUN-24	1.3	2.3.1	Clarified that participants will be screened for signs and symptoms of anemia and tested as outlined in the Schedule of Events (unchanged)
2023-JUN-24	1.3	5.1	Added DSQ-PEM as outcome
2023-JUN-24	1.3	6.2	Clarified that by “index SARS-CoV-2 infection” we mean “SARS-CoV-2 infection to which Long COVID is attributed”<br>Deleted “with persistent viral shedding from nasopharynx” at suggestion of FDA
2023-JUN-24	1.3	6.3	Clarified exclusion related to other investigational products, specifically PET tracers which have a short half-life and no biological activity outside this window.<br>Clarified exclusion criteria related to current cardiovascular disease

			Clarified exclusion criteria related to prior ME/CFS and dysautonomia
2023-JUN-24	1.3	9.1.3	Clarified that medical history may include screening for related conditions such as ME/CFS, POTS, hypermobility syndromes, endometriosis, and mast cell activation syndrome.
2023-JUN-24	1.3	9.1.6	Added additional instruments that will be used to assess medical history and outcomes at recommendation of Patient Led Research Collaborative<br><br>Added additional detail about the approach to recording Long COVID Symptoms and other symptoms (AE Review) as adverse events.<br><br>Added clarification about neurocognitive testing.
2023-JUN-24	1.3	9.2.7	Clarified that quantifiable HIV refers to values >48 copies/mL
2023-JUN-24	1.3	10.1	Added clarification that the screening procedures may be conducted over time if need be to reduce participant burden<br><br>Added clarification about medical history at screening visit
2023-JUN-24	1.3	10.2	Added option to complete Screening and Baseline assessments over multiple visits if needed to reduce participant burden
2023-JUN-24	1.3	10.5	Corrected “D14” error to “D15” to match schedule of events
2023-AUG-18	1.4	5.1	Indicated that Dysautonomia Assessment could be COMPASS-31 or a similar scale. This is because there might be licensing issues related to COMPASS-31.

2023-AUG-18	1.4	6.2	Corrected criterion 8 to indicate “In otherwise stable health prior to Screen” rather than “D0” since eligibility for the protocol must be determined prior to D0. Additionally edited criteria 4, 5, 7, 8, 9, and 23 to anchor on planned D0, since eligibility needs to be determined prior to D0. Added in section 10.3 that prior to infusion, participants will be queried prior to infusion regarding any major health changes related to these criteria and any major changes to health will be discussed with a study clinician to confirm whether the infusion should proceed.
2023-AUG-18	1.4	9.1.4	Corrected discrepancy regarding who is allowed to conduct the screening physical examination, to make consistent with section 10.1. A qualified study clinician (physician, nurse, or physician assistant) may conduct this examination.
2023-AUG-18	1.4	9.1.6	Indicated that Dysautonomia Assessment could be COMPASS-31 or a similar scale. This is because there might be licensing issues related to COMPASS-31.
2023-AUG-18	1.4	10.3	Edited as per item 6.2 above to include confirmation of no major health changes prior to infusion.
2023-AUG-18	1.4	10.4	Added DSQ-PEM at the time of the phone follow-up.
2023-AUG-18	1.4	SOE	Indicated that Dysautonomia Assessment could be COMPASS-31 or a similar scale. This is because there might be licensing issues related to COMPASS-31.
2023-AUG-18	1.4	SOE	Indicated that the DSQ-PEM would be administered at the phone follow-up at 1M visit
2023-NOV-16	1.5	10.1	Specified that rescreening can occur.
2023-NOV-16	1.5	SOE	Removed Long COVID symptom assessment from the D1 phone follow-up. Note: The AE assessment and DSQ-PEM (post-exertional malaise) questionnaire still take place at this time point, but there is no need to ascertain Long COVID symptoms at this time point.

2023-NOV-16	1.5	SOE	Added DSQ-PEM to 2W assessment (SMC recommendation)
2023-NOV-16	1.5	SOE	Added PGIC scale at 3-, 6-, and 12-month timepoints.

CONSORT 2025 checklist of information to include when reporting a randomised trial*

Section / Topic	No	CONSORT 2025 checklist item description	Reported on page no.
**Title and abstract**			
Title and structured abstract	1a	Identification as a randomised trial	3
	1b	Structured summary of the trial design, methods, results, and conclusions	3
**Open science**			
Trial registration	2	Name of trial registry, identifying number (with URL) and date of registration	4
Protocol and statistical analysis plan	3	Where the trial protocol and statistical analysis plan can be accessed	22
Data sharing	4	Where and how the individual de-identified participant data (including data dictionary), statistical code and any other materials can be accessed	22
Funding and conflicts of interest	5a	Sources of funding and other support (e.g., supply of drugs), and role of funders in the design, conduct, analysis and reporting of the trial	27-28
	5b	Financial and other conflicts of interest of the manuscript authors	28
**Introduction**			
Background and rationale	6	Scientific background and rationale	4
Objectives	7	Specific objectives related to benefits and harms	4
**Methods**			
Patient and public involvement	8	Details of patient or public involvement in the design, conduct and reporting of the trial	15
Trial design	9	Description of trial design including type of trial (e.g., parallel group, crossover), allocation ratio, and framework (e.g., superiority, equivalence, non-inferiority, exploratory)	15
Changes to trial protocol	10	Important changes to the trial after it commenced including any outcomes or analyses that were not prespecified, with reason	N/A
Trial setting	11	Settings (e.g., community, hospital) and locations (e.g., countries, sites) where the trial was conducted	15
Eligibility criteria	12a	Eligibility criteria for participants	15, supplementary table 9

	12b	If applicable, eligibility criteria for sites and for individuals delivering the interventions (e.g., surgeons, physiotherapists)	N/A
Intervention and comparator	13	Intervention and comparator with sufficient details to allow replication. If relevant, where additional materials describing the intervention and comparator (e.g., intervention manual) can be accessed	16
Outcomes	14	Pre-specified primary and secondary outcomes, including the specific measurement variable (e.g., systolic blood pressure), analysis metric (e.g., change from baseline, final value, time to event), method of aggregation (e.g., median, proportion), and time point for each outcome	16-18, supplementary table 10
Harms	15	How harms were defined and assessed (e.g., systematically, non-systematically)	7
Sample size	16a	How sample size was determined, including all assumptions supporting the sample size calculation	13
	16b	Explanation of any interim analyses and stopping guidelines	N/A
Randomisation:			
Sequence generation	17a	Who generated the random allocation sequence and the method used	15
	17b	Type of randomisation and details of any restriction (e.g., stratification, blocking and block size)	15
Allocation concealment mechanism	18	Mechanism used to implement the random allocation sequence (e.g., central computer/telephone; sequentially numbered, opaque, sealed containers), describing any steps to conceal the sequence until interventions were assigned	15
Implementation	19	Whether the personnel who enrolled and those who assigned participants to the interventions had access to the random allocation sequence	15
Blinding	20a	Who was blinded after assignment to interventions (e.g., participants, care providers, outcome assessors, data analysts)	15
	20b	If blinded, how blinding was achieved and description of the similarity of interventions	9, 15
Statistical methods	21a	Statistical methods used to compare groups for primary and secondary outcomes, including harms	21-22
	21b	Definition of who is included in each analysis (e.g., all randomised participants), and in which group	21
	21c	How missing data were handled in the analysis	21
	21d	Methods for any additional analyses (e.g., subgroup and sensitivity analyses), distinguishing prespecified from post-hoc	21-22
**Results**			
Participant flow, including flow diagram	22a	For each group, the numbers of participants who were randomly assigned, received intended intervention, and were analysed for the primary outcome	6, figure 1
	22b	For each group, losses and exclusions after randomisation, together with reasons	9, figure 1 (per protocol analysis)

Recruitment	23a	Dates defining the periods of recruitment and follow-up for outcomes of benefits and harms	6
	23b	If relevant, why the trial ended or was stopped	N/A
Intervention and comparator delivery	24a	Intervention and comparator as they were actually administered (e.g., where appropriate, who delivered the intervention/comparator, how participants adhered, whether they were delivered as intended [fidelity])	6
	24b	Concomitant care received during the trial for each group	14
Baseline data	25	A table showing baseline demographic and clinical characteristics for each group	Table 1
Numbers analysed, outcomes and estimation	26	For each primary and secondary outcome, by group:<br>● the number of participants included in the analysis<br>● the number of participants with available data at the outcome time point<br>● result for each group, and the estimated effect size and its precision (such as 95% confidence interval)<br>● for binary outcomes, presentation of both absolute and relative effect size	6-8, figures 2-4
Harms	27	All harms or unintended events in each group	7, supplementary tables 2, 3
Ancillary analyses	28	Any other analyses performed, including subgroup and sensitivity analyses, distinguishing pre-specified from post-hoc	9-11
**Discussion**			
Interpretation	29	Interpretation consistent with results, balancing benefits and harms, and considering other relevant evidence	12-14
Limitations	30	Trial limitations, addressing sources of potential bias, imprecision, generalisability, and, if relevant, multiplicity of analyses	12-14

*We strongly recommend reading this statement in conjunction with the CONSORT 2025 Explanation and Elaboration and/or the CONSORT 2025 Expanded Checklist for important clarifications on all the items. We also recommend reading relevant CONSORT extensions. See www.consort-spirit.org.

Citation: Hopewell S, Chan AW, Collins GS, Hróbjartsson A, Moher D, Schulz KF, et al. CONSORT 2025 Statement: updated guideline for reporting randomised trials. BMJ. 2025; 388:e081123. https://dx.doi.org/10.1136/bmj-2024-081123.

© 2025 Hopewell et al. This is an Open Access article distributed under the terms of the Creative Commons Attribution License (https://creativecommons.org/licenses/by/4.0/), which permits unrestricted use, distribution, and reproduction in any medium, provided the original work is properly cited.
