# Supplementary material for: SARS-CoV-2-specific monoclonal antibody AER002 in Long COVID: an exploratory randomized phase 2a mechanistic trial
Source: Nat Commun. 2026 Sep 30;17:11205. doi: 10.1038/s41467-026-77925-y (PMC13720204; doi:10.1038/s41467-026-77925-y)
Supplement: Supplementary file 2 [file 41467_2026_77925_MOESM2_ESM.pdf]

nature portfolio

Corresponding author(s): Michael Peluso

Last updated by author(s): Jul 30, 2026

Reporting Summary

Nature Portfolio wishes to improve the reproducibility of the work that we publish. This form provides structure for consistency and transparency in reporting. For further information on Nature Portfolio policies, see our Editorial Policies and the Editorial Policy Checklist.

Statistics

For all statistical analyses, confirm that the following items are present in the figure legend, table legend, main text, or Methods section.

n/a	Confirmed	
☐	☒	The exact sample size (*n*) for each experimental group/condition, given as a discrete number and unit of measurement
☐	☒	A statement on whether measurements were taken from distinct samples or whether the same sample was measured repeatedly
☐	☒	The statistical test(s) used AND whether they are one- or two-sided<br>*Only common tests should be described solely by name; describe more complex techniques in the Methods section.*
☐	☒	A description of all covariates tested
☐	☒	A description of any assumptions or corrections, such as tests of normality and adjustment for multiple comparisons
☐	☒	A full description of the statistical parameters including central tendency (e.g. means) or other basic estimates (e.g. regression coefficient) AND variation (e.g. standard deviation) or associated estimates of uncertainty (e.g. confidence intervals)
☐	☒	For null hypothesis testing, the test statistic (e.g. *F*, *t*, *r*) with confidence intervals, effect sizes, degrees of freedom and *P* value noted<br>*Give P values as exact values whenever suitable.*
☐	☒	For Bayesian analysis, information on the choice of priors and Markov chain Monte Carlo settings
☐	☒	For hierarchical and complex designs, identification of the appropriate level for tests and full reporting of outcomes
☐	☒	Estimates of effect sizes (e.g. Cohen's *d*, Pearson's *r*), indicating how they were calculated

Our web collection on statistics for biologists contains articles on many of the points above.

Software and code

Policy information about availability of computer code

Data collection	No software used for data collection.
Data analysis	R (version 4.4.1, and version 4.5), clusterProfiler (version 4.16.0), NONMEM (ICON, plc, version 7.6), NPX™ Map (version 1.3.0), ngs2counts (version 5.1.0), emmeans(version 1.11.2). ggplot2 (version 3.5.2). nlme version (3.1). OsiriX DICOM viewer software package (Pixmeo).

For manuscripts utilizing custom algorithms or software that are central to the research but not yet described in published literature, software must be made available to editors and reviewers. We strongly encourage code deposition in a community repository (e.g. GitHub). See the Nature Portfolio guidelines for submitting code & software for further information.

Data

Policy information about availability of data

All manuscripts must include a data availability statement. This statement should provide the following information, where applicable:

- Accession codes, unique identifiers, or web links for publicly available datasets
- A description of any restrictions on data availability
- For clinical datasets or third party data, please ensure that the statement adheres to our policy

Data underlying the main figures are provided in the Supplementary Information and Source Data files. Additional deidentified participant-level data are available from the corresponding author upon reasonable request. Public release is restricted because the study consent and ethical approvals do not permit unrestricted sharing of participant-level data and because certain data elements may pose a risk of re-identification. Requests from qualified researchers for research consistent with the original consent should be submitted to the corresponding author and will be reviewed by the study investigators and relevant institutional authorities.

Complete requests will generally receive a response within six weeks. Approved access will be provided under a data use agreement for the duration of the approved project.

Research involving human participants, their data, or biological material

Policy information about studies with human participants or human data. See also policy information about sex, gender (identity/presentation), and sexual orientation and race, ethnicity and racism.

Reporting on sex and gender	Sex assigned at birth and gender were self-reported by participants at the initial screening visit. Care was taken to distinguish between the two in data reporting and to recruit a balanced cohort (56% female sex, 44% male sex). Sex- and gender-based analyses were not conducted due to the small sample size of the study.
Reporting on race, ethnicity, or other socially relevant groupings	Race and ethnicity was self-reported by participants at the initial screening visit. Participants were asked to choose from NIH race and ethnicity categories as required for NIH-funded research in the U.S.
Population characteristics	36 participants were enrolled and randomized to receive AER002 versus placebo in a 2:1 ratio. Median age was 42 years (interquartile range [IQR]: 32-53); 56% of participants were female sex; 75% were White, 14% Asian, 8% Hispanic/Latino, and 3% Black (Table 1). Pre-existing medical comorbidities included: lung problems (asthma, COPD, emphysema, or bronchitis; 17%), autoimmune disease (14%), hypertension (11%), diabetes (2.8%), and HIV (2.8%). Participants with a diagnosis of myalgic encephalomyelitis/chronic fatigue syndrome (ME/CFS) preceding their SARS-CoV-2 infection were excluded. Prior to infusion, 5.6% of participants had received a mast cell activation syndrome (MCAS) diagnosis and 8.3% met criteria for hypermobile Ehlers-Danlos Syndrome. Participants of female sex were also evaluated for pain symptoms related to endometriosis (5.6% score ≥60). All participants met the WHO criteria for Long COVID prior to enrollment.
Recruitment	Participants were recruited from the University of California, San Francisco (UCSF)-based Long-term Impact of Infection with Novel Coronavirus (LIINC) cohort (NCT04362150), which enrolls and prospectively measures adults in the post-acute phase of SARS-CoV-2 infection. All participants were required to enroll in LIINC prior to screening for this trial. Briefly, interested LIINC participants completed a screening visit at which their eligibility for this trial was assessed. The trial enrolled adults (≥18 years) with clinical evidence of Long COVID, defined as at least two moderate or severe symptoms that were new or worsened since the time of SARS-CoV-2 infection and were not known to be attributable to another cause, which had been present for at least 60 days following a test-confirmed SARS-CoV-2 infection that occurred in the period in which AER002 was expected to have neutralizing activity against circulating variants (i.e., prior to August 15, 2022). Our definition was based on the World Health Organization consensus definition; although not available when the study was planned, our definition is consistent with that which has since been put forth by the National Academies of Sciences, Engineering, and Medicine. We excluded those who had ever previously received a SARS-CoV-2-specific monoclonal antibody, had received COVID-19 convalescent plasma or a SARS-CoV-2 vaccine within the preceding 60 days, who had major surgery within the prior 6 months, had medical comorbidities including active cardiovascular disease (defined as recent myocardial infarction or stroke, or history of congestive heart failure or pulmonary arterial hypertension), viral hepatitis, uncontrolled HIV infection, coagulopathy, severe anemia, moderate or severe immunocompromise according to the 2023 CDC COVID-19 guidelines, and those with a history of anaphylaxis or similar significant allergic reaction. We also excluded those with a diagnosis of ME/CFS or dysautonomia that preceded their SARS-CoV-2 infection.
Ethics oversight	The study was approved by the University of California, San Francisco Institutional Review Board.

Note that full information on the approval of the study protocol must also be provided in the manuscript.

Field-specific reporting

Please select the one below that is the best fit for your research. If you are not sure, read the appropriate sections before making your selection.

[x] Life sciences [ ] Behavioural & social sciences [ ] Ecological, evolutionary & environmental sciences

For a reference copy of the document with all sections, see nature.com/documents/nr-reporting-summary-flat.pdf

Life sciences study design

All studies must disclose on these points even when the disclosure is negative.

Sample size	36 participants were recruited and enrolled. The sample-size reflects the study's exploratory, proof-of-concept, phase 2a design and primary emphasis on safety, tolerability, feasibility, pharmacokinetics, and mechanistic outcomes.<br><br>For PROMIS-29, assuming a standard deviation (SD) of the within-person difference of 10, the original planned sample size (n=30) had an 80% power to detect a difference of 11.2 on a two-sided 0.05 level test. For biomarkers, such as IL-6, the original planned sample size (n=30) had an 80% power to detect a difference from a mean value of 2.5 pg/mL to 1.1 times the within-person standard deviation.
Data exclusions	All participants completed the trial and were included in the main analysis. A per-protocol analysis was also conducted in which we excluded individuals with confirmed and suspected on-study reinfections before the primary endpoint (90 days). The results were consistent with the main analysis.
Replication	Reproducibility was promoted throughout the study via the use of validated measurement instruments and certified laboratories. Data monitoring was conducted on a frequent basis via an internal study monitor and an external Data and Safety Monitoring Board. Intention-to-treat and per-protocol analyses were conducted where applicable. Study protocol and statistical analysis plan are publicly available on ClinicalTrials.gov (NCT05877508).

Randomization	Participants were randomized (2:1) by pharmacy staff shortly before administration of the infusion of AER002 or placebo.
Blinding	outSMART-LC was double-blind. All participants and study personal were blinded until the last participant completed the 180-day timepoint; participants were then followed in an open-label manner for safety until the 360-day timepoint.

Reporting for specific materials, systems and methods

We require information from authors about some types of materials, experimental systems and methods used in many studies. Here, indicate whether each material, system or method listed is relevant to your study. If you are not sure if a list item applies to your research, read the appropriate section before selecting a response.

Materials & experimental systems

n/a	Involved in the study
☒	☐ Antibodies
☒	☐ Eukaryotic cell lines
☒	☐ Palaeontology and archaeology
☒	☐ Animals and other organisms
☐	☒ Clinical data
☒	☐ Dual use research of concern
☒	☐ Plants

Methods

n/a	Involved in the study
☒	☐ ChIP-seq
☒	☐ Flow cytometry
☒	☐ MRI-based neuroimaging

Clinical data

Policy information about clinical studies

All manuscripts should comply with the ICMJE guidelines for publication of clinical research and a completed CONSORT checklist must be included with all submissions.

Clinical trial registration	ClinicalTrials.gov ID: NCT05877508
Study protocol	The protocol and statistical analysis plan are available on clinicaltrials.gov (NCT05877508).
Data collection	Participants were enrolled from July 2023 to April 2024 at Zuckerberg San Francisco General Hospital in San Francisco, California. Data was collected through May 2025.
Outcomes	Investigators chose a wide diversity of patient-reported and objective outcomes used in previous Long COVID and ME/CFS clinical research. The PROMIS-29 Physical Health Summary Score was selected as the primary endpoint due to its well-validated, comprehensive nature and frequent use in research concerning Long COVID and other chronic health conditions.

Plants

Seed stocks	N/A
Novel plant genotypes	N/A
Authentication	N/A
