# Supplementary material for: SARS-CoV-2-specific monoclonal antibody AER002 in Long COVID: an exploratory randomized phase 2a mechanistic trial
Source: Nat Commun. 2026 Sep 30;17:11205. doi: 10.1038/s41467-026-77925-y (PMC13720204; doi:10.1038/s41467-026-77925-y)
Supplement: Supplementary file 3 [file 41467_2026_77925_MOESM3_ESM.pdf]

nature portfolio

Peer Review File

SARS-CoV-2-Specific Monoclonal Antibody AER002 in Long COVID: An Exploratory Randomized Phase 2a Mechanistic Trial

Corresponding Author: Dr Michael Peluso

This file contains all reviewer reports in order by version, followed by all author rebuttals in order by version.

Version 0:

Reviewer comments:

Reviewer #1

(Remarks to the Author)
The authors present a study evaluating the efficacy of the SARS-CoV-2–specific monoclonal antibody AER002 for the treatment of Long COVID. The manuscript is clearly written, and the discussion of the results is generally thorough. However, a major limitation is the small sample size, which is not adequately justified in the main manuscript.
Major: The clinical study protocol indicates that the trial was designed as an exploratory study with a primary focus on safety and tolerability. According to the protocol, the planned sample size (n = 30) provides 80% power to detect a difference of 11.2, assuming a standard deviation of the within-person difference of 10. This corresponds to a very large effect size requirement. For a subjective endpoint such as the PROMIS-29 score in patients with Long COVID, expecting such a large treatment effect appears unrealistic. Detecting smaller, yet clinically meaningful differences would have required a substantially larger sample size. Therefore the whole study seems severely underpowered.
A more targeted patient selection strategy (e.g. study inclusion based on a biomarker indicative of treatment response - such as positive tissue biopsies) - might have justified a smaller cohort. A biomarker might also have been a more realistic primary endpoint. However, this approach was not implemented, and only one subject showed a positive biopsy.
Given the current sample size and design, the manuscript would be more appropriately structured around safety or pharmacokinetics (already reported for healthy volunteers), rather than efficacy.
Further, the heterogeneous patient population (Broad Long COVID definition) likely diluted potential treatment effects and obscured subgroup signals.
The timing of intervention (median about 2 years post infection) may be too late if viral persistence is only relevant in earlier disease stages.
Minor: For improvement of the manuscript we would also suggest to move lines 118-123 from the background since these statements describe study results and should not be presented in the background section.

Reviewer #2

(Remarks to the Author)
The manuscript by Peluso et. al. describes a placebo controlled study in which a long acting antibody AER002 is used to treat Long-Covid. The antibody was well tolerated in the patient population, however AER002 did not improve Long-Covid symptoms in the active arm. Despite the outcome, the study was well designed and provides important information on the contribution of SARS-CoV-2 antibodies in the progression of Long-Covid. Specific comments below:

- The authors use an array to assess the concentration and type (S, S1, N) of anti-spike antibodies in the individuals enrolled in the study. While informative, it is necessary to understand the quality of the antibody response vs. variants that are likely to contribute to Long-Covid. The authors should test serum neutralizing antibody levels at baseline and post treatment against the XBB and JN.1 variants thought to be the causal infection.

- Baseline nAb appears to be more predictive of response in both study arms. Does this suggest immune response at the time of infection is more predictive of development of Long-Covid than antibodies delivered post treatment?

- The authors measure levels of AER002 over the course of the study, however it is not clear if this is a protective level of antibody, how was the dose selected?

- How does the population included in this study compare with the population in (PMID: 37944296) in which complete resolution was reported in a small number of patients?

- The responder AUC analysis should be removed. As the study shows no difference between AER002 and placebo arms, it is not clear how the AUC analysis should be interpreted.

Reviewer #3

(Remarks to the Author)
I co-reviewed this manuscript with one of the reviewers who provided the listed reports. This is part of the Nature Communications initiative to facilitate training in peer review and to provide appropriate recognition for Early Career Researchers who co-review manuscripts.

Reviewer #4

(Remarks to the Author)
The paper by Pelusso and collaborators reports a phase 2, placebo-controlled, double-blind, randomized clinical trial that evaluates the effect of a single infusion of monoclonal antibody AER002 on PROMIS-29 and physical health summary score at 90 days in 36 patients with Long COVID.
The study did not show a significant difference between treatment groups. However, a post-doc analysis found that participants with lower baseline SARS-CoV-2 antibody levels and higher exposure tended to respond more. This is a well-designed, clinical trial and although results were negative, it shed light on the use of monoclonal antibodies for the treatment of Long COVID. Below my comments:

1-Why was the primary outcome was measured at 90 days post-infusion?
2-The sample size is very small. Was the study powered to find differences on PROMIS-29 between treatment groups?
3-Why were they baseline differences in antibodies between placebo and AER002 treatment group?
4-The standing test should be reported not as "positive or negative". Changes in blood pressure and heart rate should be analyzed at different timepoints and differences in orthostatic intolerance (orthostatic time), orthostatic HR and BP changes should be reported separately
5- COMPASS-31 values are normal at baseline, which indicates that this cohort did not have autonomic symptoms.

Reviewer #5

(Remarks to the Author)
The paper describes a phase 2 randomized trial of a monoclonal antibody (AER002) in people with long COVID. The study appears to be well-designed and carried out. Although there appeared to be little or no significant positive effects of the intervention, the data are presented in such a way that much can be learned from the study. For example, it is nice to have estimated effects with 95% confidence intervals listed in Table 3. I have a few specific comments.

Specific comments:

1. (lines 199-206). For the results of the Patient Global Impression of Change (PGIC) scale, it would be nice to have an effect estimate with 95% confidence interval (as in the results in Table 3). Since this is an ordinal scale the statistical analysis plan said that you “may summarize effects quantifying the treatment effects using the proportional odds model” and give an estimate and a 2-sided 95% confidence interval. Something like that would be nice, because all you have is “not significant; Figure 2f”, which is not very informative. Figure 2f appears to favor the placebo arm, but I cannot tell from the graph what is the estimated effect and its confidence interval.

2. (Table 3 and Supplemental Table 4) These two tables have the same captions, yet are giving slightly different results. Please add to the captions to allow readers to differentiate between the tables without going to the text description within the paper.

3. (Supplemental Figure 1) This Figure is beautiful, but after looking at it for a long time, I still am having a hard time figuring out if there is a hint of a signal or not. Some of the participants have their stripe moving up or down between periods but ending up in the same category despite that. Consider using a different representation of that data.

4. (Figure 5, lines 256-262) The description of the baseline antibody measurements seems out of place. You are describing a difference in the randomized groups at baseline. In effect you are describing an “unlucky” randomization, because there are “statistically significant” effects that are known to be caused by randomization. To me, this paragraph and Figure 5 belong in the Cohort description section, since Figure 5 is taking on a role similar to Tables 1 and 2; it is describing the

population at baseline and how the randomization worked out.

5. (lines 413-420) When you test for differences between responders and non-responders with respect to baseline anti-S, anti-S21, and anti-RBD antibodies, I think you should mention the results of Figure 5 (there are “significant” baseline differences in baseline antibody between the treatment arms). (Although this is properly mentioned in the discussion (lines 499-502), if the editors allow it, it would be a kindness to the reader to remind them here.) Because of Figure 5, the within-group logistic regression models (described in lines 416-418) are more important. Can you give those specific results (e.g., estimated odds and confidence intervals) as a supplement? This is especially important because you mention it again in the discussion as a result that may inform future work (lines 471-472).

6. (line 422) Please define AUC here. Later in lines 914-915 you define it as “area under the concentration-time curve from noncompartmental analysis”. Is this AUC standardized per day? The units are “mg*day/L” so it appears this is average area under the curve of concentration per day. Maybe that is standard terminology for pharmacokinetics, but for the non-specialist please describe it for the reader when you first use it. (If the editors agree.)

7. (Ref. 51) The author is incorrect in the reference. The author is John D. Storey.

Open Access This Peer Review File is licensed under a Creative Commons Attribution 4.0 International License, which permits use, sharing, adaptation, distribution and reproduction in any medium or format, as long as you give appropriate credit to the original author(s) and the source, provide a link to the Creative Commons license, and indicate if changes were made.
In cases where reviewers are anonymous, credit should be given to 'Anonymous Referee' and the source.
The images or other third party material in this Peer Review File are included in the article’s Creative Commons license, unless indicated otherwise in a credit line to the material. If material is not included in the article’s Creative Commons license and your intended use is not permitted by statutory regulation or exceeds the permitted use, you will need to obtain permission directly from the copyright holder.
To view a copy of this license, visit https://creativecommons.org/licenses/by/4.0/

RESPONSE TO REVIEWERS

We are grateful for the thorough and thoughtful feedback provided by the reviewers and editor, which has substantially strengthened this manuscript. We hope that the revisions outlined below address the reviewers' concerns, and we would welcome the opportunity to provide any additional information the editors may find helpful. Line references refer to the clean resubmission file and not the highlighted version demonstrating changes since previous submission.

<u>Reviewer 1</u>:

The clinical study protocol indicates that the trial was designed as an exploratory study with a primary focus on safety and tolerability. According to the protocol, the planned sample size (n = 30) provides 80% power to detect a difference of 11.2, assuming a standard deviation of the within-person difference of 10. This corresponds to a very large effect size requirement. For a subjective endpoint such as the PROMIS-29 score in patients with Long COVID, expecting such a large treatment effect appears unrealistic. Detecting smaller, yet clinically meaningful differences would have required a substantially larger sample size. Therefore the whole study seems severely underpowered.

Response: We agree with the reviewer that the study was not powered to detect small or moderate differences in the PROMIS-29 Physical Health Summary Score and that a substantially larger sample would have been required to assess clinically meaningful efficacy with adequate precision. The sample-size calculation reflected the exploratory phase 2a design and the study's primary emphasis on safety, tolerability, feasibility, pharmacokinetics, and mechanistic outcomes.

At the time the study was designed, there were few randomized trials of therapeutic interventions for Long COVID and no prior randomized trial of a SARS-CoV-2-specific monoclonal antibody in this clinical population (see "Where are the Long COVID Trials?," Lancet Editorial Board, *Lancet Infectious Diseases* 2023). There was also skepticism among key stakeholders including funders and industry partners that trials for Long COVID could recruit participants or be conducted with rigor. At the time, we and others argued that small, rigorously conducted proof-of-concept trials were needed to provide foundational safety and biological data needed to determine whether, and how, an intervention should be advanced into a larger efficacy trial (see Davis et al *Nat Rev Microbiol* 2023, McCorkell and Peluso *Nature* 2023, Peluso and Deeks *Cell* 2024). Such larger trials are now being planned, including trials using currently available and next-generation monoclonal antibodies that are built on the principles established by this study – specifically our approach in assessing a combination of patient-reported, objective, and biomarker-based outcomes, and dosing strategies influenced by our exploratory pharmacokinetic modeling. Our approach in the reported trial is consistent with expert recommendations emphasizing the value of mechanistically informative experimental-medicine studies in Long COVID (see Proal et al *Lancet Infectious Diseases* 2025). Because these next-generation trials are actively being designed now, we believe timely publication of our full approach and findings will directly inform their design and conduct – underscoring the manuscript's value to the field today, not merely its historical rationale.

To address this comment, we carefully considered how the results were presented and have revised the manuscript to more clearly describe the study as an exploratory, proof-of-concept trial (lines 1, 54, 107, 115-118, 427, 511, 599-601); to acknowledge that it was underpowered to detect smaller, potentially clinically meaningful treatment effects (lines 504-505, 511-513, 599-601); and to avoid overinterpreting the nonsignificant clinical findings as definitive (lines 434-436, 476-477). We have also emphasized that the clinical and mechanistic findings should be considered hypothesis-generating and used to inform the design, participant selection, dosing, and sample-size assumptions of future trials (lines 115-118, 434-436, 473-477, 556-562). We further emphasize the challenges related to the use of the instruments we employed here –

common measures in Long COVID trials – and the need for the development of validated instruments that are sensitive to meaningful clinical change for this condition (lines 543-549).

A more targeted patient selection strategy (e.g. study inclusion based on a biomarker indicative of treatment response - such as positive tissue biopsies) - might have justified a smaller cohort. A biomarker might also have been a more realistic primary endpoint. However, this approach was not implemented, and only one subject showed a positive biopsy.

Response: We agree that biomarker-enriched enrollment and use of a validated biomarker as a primary or co-primary endpoint could have increased the biological specificity and efficiency of this proof-of-concept trial. However, no validated biomarker of SARS-CoV-2 persistence or predictor of response to viral-targeting therapy was available when the study was designed, and no such biomarker is currently established for clinical-trial eligibility in Long COVID. This remains a major challenge for the field to address.

Rectosigmoid biopsy was included as an optional exploratory procedure rather than a screening tool. Although detection of viral RNA in tissue may provide evidence of persistence, the sensitivity of a negative rectosigmoid biopsy is unknown. Viral persistence may be focal, may occur in other gastrointestinal regions or extraintestinal tissues, and may not be consistently detected using a single tissue sample or assay. Requiring a positive biopsy would therefore have risked excluding participants with biologically relevant persistence on the basis of an unvalidated and potentially insensitive measure. In addition, an invasive tissue procedure would not have been practical as a universal screening strategy and is unlikely to have been approved by the regulatory agencies providing oversight of the trial. Indeed, entire research programs now exist to try to develop and validate methodologies for assessing tissue persistence of SARS-Cov-2.

In our exploratory analyses, only one participant had detectable SARS-CoV-2 RNA in colorectal tissue at baseline, which underscores both the limitations of the available assay and the possibility that few enrolled participants had detectable persistence in this tissue compartment. We have revised the manuscript to more explicitly acknowledge that the absence of biomarker-based enrichment may have reduced the likelihood of detecting a treatment effect and to emphasize that future trials should incorporate validated, and preferably minimally invasive, biomarkers of viral persistence when such measures become available. However, there is broad consensus among investigators, clinicians, and patient advocates in this field that we cannot wait for such a biomarker before pursuing interventional studies – indeed analyses of biospecimens from such exploratory studies may ultimately be needed to identify such a marker.

To address this comment, we have further elaborated on our prior comment acknowledging the lack of biomarker-based enrollment as a limitation of the study in the Discussion and Limitations section (lines 443-445, 517-525). This concept is now emphasized throughout the paper, underscoring the importance of identifying such biomarkers for the field.

Given the current sample size and design, the manuscript would be more appropriately structured around safety or pharmacokinetics (already reported for healthy volunteers), rather than efficacy.

Response: We agree that the sample size precludes definitive conclusions regarding clinical efficacy. That said, we do not believe the manuscript should be structured solely around safety or pharmacokinetics. There is a long history across multiple fields, including HIV cure research, oncology, immunology, and experimental medicine, of small, randomized, proof-of-concept trials designed to assess not only safety, but also mechanistic outcomes. Such studies are intended to test biological hypotheses, generate preliminary estimates of treatment effect, identify potential correlates or modifiers of response, and inform the design of larger confirmatory trials.

The present study was conceived in this framework. Safety and tolerability were central objectives, but the clinical, pharmacokinetic, imaging, tissue-based, and immune analyses were integral to exploring whether AER002 produced evidence of biological or clinical activity in people with Long COVID.

To address this comment, we have revised the manuscript to more clearly characterize the trial as exploratory and hypothesis-generating and to avoid overinterpreting nonsignificant findings (lines 1, 54, 107, 115-118, 427, 434-436, 473-477, 504-505, 511, 556-558). We have retained the prespecified clinical and mechanistic outcomes because we believe they remain central to the scientific rationale and translational contribution of the study, while making clear throughout that these findings are hypothesis-generating rather than confirmatory. We have also added a more direct comment in the Methods to address this concern (lines 599-601).

Further, the heterogeneous patient population (Broad Long COVID definition) likely diluted potential treatment effects and obscured subgroup signals.

Response: We agree that clinical and biological heterogeneity may have reduced the ability to detect a treatment effect. The World Health Organization consensus definition used in our study was the accepted definition at the time that the study was conducted, since that time, the National Academies of Sciences, Engineering, and Medicine definition has become standard; both definitions are broad. Although all participants met the accepted case definition for Long COVID laid forth by the WHO, the condition encompasses multiple symptom phenotypes and potentially distinct underlying mechanisms. A therapy directed at SARS-CoV-2 persistence – or any therapy for that matter – would therefore not necessarily be expected to benefit all individuals meeting a broad clinical definition.

We adapted the WHO definition by requiring several additional components: (1) test-confirmed SARS-CoV-2 infection (the WHO definition and the NASEM definition both accommodate suspected cases); (2) requiring at least two Long COVID related symptoms from different domains; (3) requiring symptoms to be at least moderately debilitating. While we considered limiting the population screened to a specific Long COVID phenotype to minimize heterogeneity, we chose not to do so because there were no preliminary data available to guide which clinical symptoms might be most strongly associated with viral persistence and because many key stakeholders including regulators, clinicians, researchers, and people with lived experience were generally not supportive of that approach at the time when the study was conducted. Other trials have taken a similar approach (see Geng et al JAMA Int Med 2024, Sawano et al Lancet Infect Diseases 2023). Now that an initial set of trials targeting viral persistence in broadly defined Long COVID has been reported, there is emerging consensus that future trials should employ more narrow case definitions (if small or moderately sized) or enroll adequate numbers of individuals to allow subgroup comparisons between phenotypes (if large).

However, we also note that our study population had a high frequency of common Long COVID symptoms (92% with fatigue, 100% with neurocognitive symptoms, 81% with cardiopulmonary symptoms, 86 with post-exertional malaise; see Results); therefore although it is broadly true that Long COVID is a heterogeneous condition, a high proportion of our participants had typical Long COVID symptoms. This suggests that our findings are likely to generalize to the broader Long COVID population encountered in clinical practice, supporting the relevance of this study to a general medical and scientific readership interested in Long COVID.

Validated biomarkers or clinical phenotypes capable of identifying individuals most likely to have persistence-driven disease are simply not available. We therefore used broad clinical eligibility criteria appropriate for an initial proof-of-concept study. This approach improved generalizability but may have diluted a treatment effect confined to a biologically defined subgroup and limited our ability to identify subgroup-specific signals in a small cohort.

To address this comment, we have revised the Discussion and Limitations to more explicitly identify clinical and biological heterogeneity as a limitation (lines 517-520) and to emphasize that future studies should incorporate biomarker enrichment, phenotype-based stratification, or both when validated selection strategies become available (lines 558-562).

The timing of intervention (median about 2 years post infection) may be too late if viral persistence is only relevant in earlier disease stages.

Response: As the reviewer notes, participants had experienced Long COVID for a median of approximately two years, and it remains possible that SARS-CoV-2 persistence is more biologically or therapeutically relevant earlier in the disease course. Over time, downstream processes such as chronic immune dysregulation, autoimmunity, vascular injury, or metabolic dysfunction may become increasingly self-sustaining and less responsive to an intervention directed solely at the virus.

At the same time, our focus on individuals infected earlier in the pandemic was clinically informed and clinically relevant. Many people with the most severe and persistent forms of Long COVID developed illness following infections during the early, pre-vaccine phases of the pandemic and remain in need of treatment. Moreover, epidemiologic studies suggest that the risk of developing Long COVID following a new infection has declined across successive pandemic eras, particularly among vaccinated individuals, although it has not been eliminated. Thus, restricting enrollment to more recent infections would have excluded an important patient population with substantial and ongoing disease burden.

Enrollment closer to the Long COVID-triggering infection was also constrained by the variant-specific activity of AER002. Eligibility was limited to participants whose qualifying infection occurred before August 15, 2022, when the antibody was expected to retain neutralizing activity against circulating variants (reflecting the rationale that AER002 needed to retain activity against the variant responsible for each participant's initial infection). By the time the trial began enrollment in Summer 2023, this necessarily selected for individuals farther from their initial infection. Where appropriate, future trials may choose to stratify the enrollment based on era of infection or duration of Long COVID, or to restrict enrollment to individuals who have been ill for a certain amount of time.

To address this comment, we have clarified this issue in the Discussion and now emphasize that future trials should evaluate interventions across different disease durations, stratify analyses by time since infection, and use agents with activity against contemporary as well as historical variants (lines 529-537).

For improvement of the manuscript we would also suggest to move lines 118-123 from the background since these statements describe study results and should not be presented in the background section.

Response: To address this comment, we have removed them from the Background and incorporated the relevant content into the opening paragraph of the Discussion (lines 429-434).

Reviewer 2:

The authors use an array to assess the concentration and type (S, S1, N) of anti-spike antibodies in the individuals enrolled in the study. While informative, it is necessary to understand the quality of the antibody response vs. variants that are likely to contribute to Long-Covid. The authors should test serum neutralizing antibody levels at baseline and post treatment against the XBB and JN.1 variants thought to be the causal infection.

Response: We agree that functional characterization of the antibody response can provide information beyond measurements of binding antibody concentration. However, XBB and JN.1 were not the variants associated with the Long COVID-triggering infections in this study. Eligibility required the qualifying infection to have occurred before August 15, 2022, when earlier ancestral, Delta, and Omicron-lineage variants were circulating. XBB and JN.1 emerged later and therefore would not represent the presumed causal variants for the participants' initial Long COVID illness.

In addition, interpretation of post-treatment neutralization assays would be complicated by the presence of infused AER002, such that measured neutralizing activity would reflect both endogenous antibodies and the study drug. AER002 also had limited or absent activity against later variants, including those in the XBB and JN.1 lineages, making these assays poorly aligned with the biological question addressed by the trial.

We agree that neutralization assays against participant-relevant infecting variants could be informative, particularly for understanding the quality of the baseline endogenous immune response. However, infecting variants were not definitively identified for most participants, and the serum volume and scope of the current analyses did not permit a comprehensive variant-specific neutralization study.

To address this comment, we have added this as a limitation and clarified that the measured anti-S, anti-S1, anti-RBD, and anti-N antibodies represent binding rather than neutralizing antibody responses (lines 539-541).

Baseline nAb appears to be more predictive of response in both study arms. Does this suggest immune response at the time of infection is more predictive of development of Long-Covid than antibodies delivered post treatment?

Response: The baseline measurements in this study quantified binding antibodies to spike, S1, RBD, and nucleocapsid rather than neutralizing antibody activity. We therefore cannot determine whether baseline neutralizing capacity predicted response.

We also do not believe these findings establish that the immune response at the time of infection was more predictive of Long COVID development. Antibody levels were measured a median of approximately two years after the Long COVID-triggering infection and were influenced by subsequent vaccination, reinfection, antibody waning, and individual variation in humoral immunity. In addition, all enrolled participants had already developed Long COVID years prior to participation in this trial, so the study was not designed to evaluate predictors of disease development.

Importantly, this analysis points to a potentially actionable prognostic biomarker: the association between higher baseline binding antibody levels and improvement in both treatment groups suggests that these measurements may identify individuals with Long COVID who are more likely to improve over the subsequent 3-6 months, independent of treatment. In this sense, baseline antibody levels may represent a prognostic rather than treatment-predictive biomarker. Conversely, the exploratory association between greater AER002 exposure and improvement among participants with lower baseline antibody levels raises the hypothesis that passive antibody administration may be more relevant in individuals with comparatively weaker endogenous humoral responses. Given the small sample size, baseline imbalance between treatment groups, multiple exploratory analyses, and absence of neutralization measurements, these findings should be interpreted cautiously and require prospective validation.

To address this comment, we have revised the Discussion to reflect these considerations (lines 467-477).

The authors measure levels of AER002 over the course of the study, however it is not clear if this is a protective level of antibody, how was the dose selected?

Response: The 1200 mg intravenous dose was selected based on the preceding phase 1 dose-escalation study in healthy adults, in which AER002 was evaluated across multiple dose levels (see Moullan et al Infect Dis Ther 2024). The 1200 mg dose was safe and well-tolerated and produced prolonged systemic exposure consistent with the antibody's extended half-life. These data supported selection of the highest previously studied single-agent dose to maximize the magnitude and duration of exposure in this initial proof-of-concept study.

There is, however, no established serum concentration of AER002, or of any SARS-CoV-2-specific monoclonal antibody, known to be protective or therapeutically active against presumed viral persistence in Long COVID. Exposure thresholds derived from prevention or treatment of acute infection may not apply to a chronic condition in which the amount, anatomical location, replication state, and accessibility of persistent viral material are uncertain. The pharmacokinetic analyses in this study were therefore intended to characterize achieved exposure and explore exposure-response relationships rather than determine whether participants exceeded a validated protective threshold.

To address this comment, we have added the rationale for dose selection to the Methods (lines 613-617) and clarified in the Discussion that the therapeutic exposure required to affect a putative tissue reservoir is unknown (lines 475-477).

How does the population included in this study compare with the population in (PMID: 37944296) in which complete resolution was reported in a small number of patients?

Response: PMID: 37944296 is an uncontrolled case series describing three highly selected individuals with severe Long COVID following pre-Delta infection who received casirivimab/imdevimab 5–18 months after their initial illness. Importantly, treatment in these cases was administered during a subsequent SARS-CoV-2 infection, so the observed improvement could have reflected effects on the new infection, the immune response to reinfection, the response to an established reservoir, or some combination of these.

In contrast, our trial enrolled a broader population and administered a different monoclonal antibody outside the setting of known acute infection, at a median of approximately two years after the Long COVID-triggering infection. The apparent difference in outcomes may therefore reflect differences in participant selection, timing, infecting variants, treatment context, and antibody characteristics. Because the prior report was an uncontrolled series of three responders, it cannot estimate the frequency or causal basis of benefit.

To address this comment, we have added a sentence to the Discussion (lines 461-465).

The responder AUC analysis should be removed. As the study shows no difference between AER002 and placebo arms, it is not clear how the AUC analysis should be interpreted.

Response: We appreciate the reviewer's concern, but do not believe the exposure-response analysis should be removed. The absence of a statistically significant difference between randomized treatment groups does not preclude a potentially informative relationship between drug exposure and response within the AER002 arm. Participants receiving the same dose exhibited substantial interindividual variability in AER002 exposure, and an exposure-response analysis addresses a different question from the intention-to-treat comparison: whether the magnitude and duration of achieved drug exposure may have influenced the likelihood of response.

This analysis is particularly relevant for an early-phase mechanistic trial because it may inform dose selection and dosing frequency in subsequent studies. The observed association was confined to participants with lower baseline anti-S, anti-S1, and anti-RBD antibody levels, providing a biologically plausible hypothesis that both host immune phenotype and adequate drug exposure may be important determinants of response. Notably,

this exposure-response signal was concentrated among participants with comparatively weaker endogenous humoral responses at baseline – precisely the subgroup for whom passive antibody administration may be mechanistically most relevant – suggesting that benefit tracked with biological need rather than simply mirroring a pre-existing advantage. Baseline antibody levels were, if anything, somewhat higher in the placebo arm at enrollment, a chance imbalance from randomization; we view this as reinforcing rather than undermining the plausibility of the finding, since improvement within the AER002 arm was concentrated among participants who started from a weaker antibody position than their placebo counterparts.

We agree that the small sample size, exploratory subgrouping, multiple comparisons, and the absence of an overall treatment effect all limit the strength of the inferences that can be drawn from this analysis.

To address this comment, we have therefore revised the manuscript to describe this analysis explicitly as exploratory and hypothesis-generating, to avoid causal interpretation, and to emphasize that the finding requires prospective validation (lines 432-436, 473-477). We have presented these results in an exploratory frame rather than as a primary or confirmatory result, and we hope the reviewer will agree that, presented this way, it retains value for informing the design of future monoclonal antibody trials.

Reviewer 3:

I co-reviewed this manuscript with one of the reviewers who provided the listed reports. This is part of the Nature Communications initiative to facilitate training in peer review and to provide appropriate recognition for Early Career Researchers who co-review manuscripts.

Response: Thank you.

Reviewer 4:

Why was the primary outcome was measured at 90 days post-infusion?

Response: Day 90 was selected to allow sufficient time for a long-acting monoclonal antibody to affect a putative persistent viral reservoir and for any downstream changes in immune activation or symptoms to become clinically apparent, although this timing reflects clinical judgment rather than definitive pharmacodynamic data. An earlier endpoint might have been too soon to capture these effects, whereas a substantially later primary endpoint would have increased the opportunity for reinfection, vaccination, medication changes, and natural fluctuations in Long COVID symptoms to confound interpretation. AER002 was expected to maintain substantial exposure over this interval because of its extended half-life. Clinical outcomes were also assessed at Days 30, 180, and 360 to evaluate the timing and durability of any treatment effect.

To address this comment, we have added this rationale to the Methods (lines 604-607).

The sample size is very small. Was the study powered to find differences on PROMIS-29 between treatment groups?

Response: As discussed in our response to Reviewer 1, the study was powered to detect a large between-group difference in the PROMIS-29 Physical Health Summary Score. The prespecified sample-size calculation indicated that 30 participants would provide 80% power to detect a difference of 11.2 points, assuming a standard deviation of 10 for the within-person change. The trial was therefore not powered to detect smaller, potentially clinically meaningful differences.

As described above, this reflects the exploratory phase 2a design and the study's primary emphasis on safety, tolerability, pharmacokinetics, and mechanistic outcomes.

To address this comment, we have clarified the sample-size calculation and this limitation in the Discussion and Methods (lines 511-513, 599-601).

Why were they baseline differences in antibodies between placebo and AER002 treatment group?

Response: The baseline differences in anti-S, anti-S1, and anti-RBD antibody levels most likely reflect chance imbalance resulting from randomization in a small study (see also response to Reviewer 5 below). Randomization was not stratified by baseline SARS-CoV-2 antibody levels, and these measurements were not available at the time of treatment allocation. There is therefore no reason to believe that the imbalance resulted from anything other than random chance.

We agree that this imbalance is important for interpretation because higher baseline antibody levels were also associated with subsequent improvement in both treatment groups. The higher antibody levels in the placebo arm may therefore have contributed to the greater frequency of reported improvement in that group and reduced the apparent between-group treatment difference.

To address this comment, we have clarified in the Results and Discussion that these baseline differences arose by chance and represent a potential confounder of the exploratory response analyses (lines 159-166, 398-399, 467-470).

The standing test should be reported not as "positive or negative". Changes in blood pressure and heart rate should be analyzed at different timepoints and differences in orthostatic intolerance (orthostatic time), orthostatic HR and BP changes should be reported separately.

Response: In the manuscript, we have summarized the results of the Active Stand Test according to the definition used by the NIH RECOVER Initiative: a decline of >20 mmHg in systolic or >10 mmHg in diastolic blood pressure, or an increase in heart rate of >30 beats per minute on two consecutive measurements (lines 743-745; see also Naschitz et al *Postgrad Med J* 2007). Therefore, we believe that the overall summary reporting in the manuscript is appropriate.

However, in response to the reviewer's comment, we also examined the individual time-based changes in the various parameters included in the test (systolic blood pressure, diastolic blood pressure, and heart rate) at the time points assessed (1 minute, 3 minutes, 5 minutes, and 10 minutes) relative to the baseline supine measurements at the D90 primary endpoint.

We did not observe meaningful differences between groups. For systolic blood pressure at D90, the mean difference between the treatment and placebo groups was between -3 and +6 mmHg over the course of the 10-minute test. For diastolic blood pressure at D90, the mean difference between the treatment and placebo groups was between +1 and +4 mmHg over the course of the 10-minute test. For heart rate at D90, the mean difference between the treatment and placebo groups was between +4 and +6 beats per minute over the course of the 10-minute test.

To address this comment, we added a sentence to the results stating that we assessed these parameters but did not observe meaningful changes within groups or differences between groups (lines 249-250).

COMPASS-31 values are normal at baseline, which indicates that this cohort did not have autonomic symptoms.

Response: We agree that baseline COMPASS-31 scores in this cohort were relatively low compared with those reported in populations selected for clinically established autonomic disorders. Our interpretation is that

the baseline scores indicate that autonomic symptoms were not a dominant feature of this cohort overall, rather than that participants had no autonomic symptoms.

This finding is consistent with the broad Long COVID eligibility criteria and the fact that participants were not selected for dysautonomia. It also clearly limits the opportunity to detect treatment-related improvement in this domain. We have revised the Results to clarify that baseline autonomic symptom burden was relatively low and that the autonomic analyses should be interpreted in this context (lines 155-156).

Reviewer 5:

(lines 199-206). For the results of the Patient Global Impression of Change (PGIC) scale, it would be nice to have an effect estimate with 95% confidence interval (as in the results in Table 3). Since this is an ordinal scale the statistical analysis plan said that you "may summarize effects quantifying the treatment effects using the proportional odds model" and give an estimate and a 2-sided 95% confidence interval. Something like that would be nice, because all you have is "not significant; Figure 2f", which is not very informative. Figure 2f appears to favor the placebo arm, but I cannot tell from the graph what is the estimated effect and its confidence interval.

Response: We agree that an effect estimate with a confidence interval provides a more informative summary than reporting statistical significance alone. We therefore fit a proportional odds model with robust Huber–White sandwich variance estimators. The estimated odds ratio was 0.26 (95% CI, 0.06–1.12; p=0.07). We have added this information in the Results (line 218-219).

(Table 3 and Supplemental Table 4) These two tables have the same captions, yet are giving slightly different results. Please add to the captions to allow readers to differentiate between the tables without going to the text description within the paper.

Response: The two tables summarize models with different follow-up windows. Table 3 includes visit terms for Day 30, where measured, Day 90, and Day 180, whereas Supplemental Table 4 additionally includes the D360 (unblinded) visit. Although this distinction was noted in the original footnote to Supplemental Table 4, we agree that this might be confusing to readers. To reduce confusion and simplify reporting, we have removed the repeated display of the D30–D180 measurements (which represent redundant information) from Supplemental Table 4, which now reports only the D360 results (Supplemental File lines 13-15).

(Supplemental Figure 1) This Figure is beautiful, but after looking at it for a long time, I still am having a hard time figuring out if there is a hint of a signal or not. Some of the participants have their stripe moving up or down between periods but ending up in the same category despite that. Consider using a different representation of that data.

Response: Thanks for this recommendation. We agree that the original figure was difficult to interpret. We have simplified Supplemental Figure 1 to display transitions between two clinically interpretable categories, PEM and no PEM, from baseline to Day 90, with flows colored by treatment group. This revised representation makes the direction and frequency of the minimal within-participant changes easier to assess while preserving the individual-level longitudinal structure of the data (Supplemental File lines 43-45).

(Figure 5, lines 256-262) The description of the baseline antibody measurements seems out of place. You are describing a difference in the randomized groups at baseline. In effect you are describing an "unlucky" randomization, because there are "statistically significant" effects that are known to be caused by randomization. To me, this paragraph and Figure 5 belong in the Cohort description section, since Figure 5 is taking on a role similar to Tables 1 and 2; it is describing the population at baseline and how the randomization worked out.

Response: Thanks for this recommendation. We agree that the baseline antibody measurements are more appropriately presented as part of the cohort description. We have moved the corresponding paragraph and Figure 5 (now Figure 2) to the Cohort description section (lines 159-166), where they are presented alongside the other baseline characteristics. We have also clarified that the between-group differences most likely reflect chance imbalance from randomization in this small study (lines 159-166, 398-399, 467-470).

In doing this, we recognized that several other descriptions of performance on other patient-reported and objective instruments should be moved to the baseline characterization section. We have now done this for all primary and secondary outcome measures (lines 149-159). We note that we left the baseline descriptions related to exploratory optional outcome measures (because they are not reflective of the entire cohort) in the corresponding sections.

(lines 413-420) When you test for differences between responders and non-responders with respect to baseline anti-S, anti-S21, and anti-RBD antibodies, I think you should mention the results of Figure 5 (there are "significant" baseline differences in baseline antibody between the treatment arms). (Although this is properly mentioned in the discussion (lines 499-502), if the editors allow it, it would be a kindness to the reader to remind them here.) Because of Figure 5, the within-group logistic regression models (described in lines 416-418) are more important. Can you give those specific results (e.g., estimated odds and confidence intervals) as a supplement? This is especially important because you mention it again in the discussion as a result that may inform future work (lines 471-472).

Response: Thank you for this recommendation – we have added a reminder in the later section of the Results that these antibody levels were higher in the placebo arm at baseline, consistent with a chance imbalance from randomization in this small study (lines 398-399).

Because of this imbalance, we also agree that treatment-specific analyses are more informative than analyses combining both groups. We have therefore added the estimated odds ratios and 95% confidence intervals from the within-arm logistic regression models to Supplemental Table 8 (lines 405-406, Supplemental File lines 31-34). These models evaluate the association between higher baseline antibody levels and subsequent response separately in the AER002 and placebo groups. Given the small sample size and limited number of responders, these estimates remain imprecise and are presented as exploratory.

*(line 422) Please define AUC here. Later in lines 914-915 you define it as "area under the concentration-time curve from noncompartmental analysis". Is this AUC standardized per day? The units are "mg*day/L" so it appears this is average area under the curve of concentration per day. Maybe that is standard terminology for pharmacokinetics, but for the non-specialist please describe it for the reader when you first use it. (If the editors agree.)*

Response: We agree that AUC should be defined when it is first introduced and described adequately for non-PK-specialists. The units "mg*day/L" are standard PK units for AUC and represent the total exposure following passive antibody infusion. However, because the duration of pharmacokinetic sampling varied across participants, we calculated AUC extrapolated to infinity (AUCinf) rather than AUC to the last measured time point, which allows total exposure to be compared consistently across participants despite differences in individual follow-up duration. This is now reflected in the Results text (lines 409-411).

(Ref. 51) The author is incorrect in the reference. The author is John D. Storey.

Response: The reference manager had reversed the first and last names. We have corrected the author name John D. Storey (line 1142).
